# Single-molecule readout of reversible nanoswitches enables continuous monitoring of low biomarker concentrations

**DOI:** 10.1038/s41467-026-71690-8

**Published:** 2026-04-18

**Authors:** Chris Vu, Selina A. J. Janssen, Arthur M. de Jong, Menno W. J. Prins

**Affiliations:** 1 Department of Biomedical Engineering, Eindhoven University of Technology, Eindhoven, The Netherlands; 2 Institute for Complex Molecular Systems (ICMS), Eindhoven University of Technology, Eindhoven, The Netherlands; 3 Department of Applied Physics and Science Education, Eindhoven University of Technology, Eindhoven, The Netherlands; 4 Helia Biomonitoring, Eindhoven, The Netherlands

**Keywords:** Biosensors, Analytical chemistry, Single-molecule biophysics, Computational biophysics, Assay systems

## Abstract

Continuous sensing technologies are expected to change how dynamic bioprocesses will be monitored and controlled in the future. However, a fundamental challenge in the field of biomolecular sensing is that low concentrations invariably cause slow sensor responses. In this paper, we explain how continuous sensors can be developed for quantifying very low biomarker concentrations with fast sensor response times. The measurement concept is based on combining reversible affinity-based nanoswitches with single-molecule readout. A generalizable rate-based simulation model was developed and validated on experimental data of a sandwich-based nanoswitch. The results show how the nanoswitch design and the sensing acquisition parameters control the amplitude and stochastic variations of the sensor response, and how these affect the precision of the concentration determination. We show that the measurement precision is limited by the counting statistics of molecular sandwich events. Using realistic design parameters, we predict limits-of-quantification in the low picomolar range within measurement timescales of minutes. These results pave the way for the development of intrinsically reversible nanoswitch sensors that can access unexplored concentration-time spaces of dynamic biosystems.

## Abstract (web summary)

The continuous monitoring of low-concentration biomarkers requires the development of innovative sensing methods. Here, the authors describe a reversible nanoswitch sensing approach with single-molecule readout, that enables minute-scale quantification of biomolecules at low-picomolar concentrations.

## Introduction

Continuous biosensing technologies have the potential to enable revolutionary monitoring-and-control strategies in healthcare^1–3^, industrial bioprocessing^4–6^, and environmental safety^7^. As a proven example, the management of diabetes has been transformed by continuous glucose sensing, now widely used for the monitoring of patients and control of their treatments^8–10^. Similarly, significant impacts are expected when the continuous monitoring of inflammatory proteins will become possible, in order to guide therapeutic interventions in severely ill patients^2^, and when proteins and nucleic-acids can be monitored in real time to achieve optimal control in industrial bioprocesses^4,6^. However, for these applications, substances need to be measured that have concentrations much lower than glucose (micromolar to picomolar, rather than millimolar), and cannot be measured using the enzymatic-electrochemical principles that underlie present-day continuous glucose sensors.

To monitor molecules at low concentrations, affinity-based nanoswitch sensors are being designed and investigated. These are nano-engineered constructs with distinct configurations that are controlled by reversible affinity interactions. Examples are conformationally switching aptamers^11^, conformational probes with fluorescence readout^12,13^, electrically modulated dsDNA molecules^14–16^, DNA origami-based switches^17,18^, and molecularly tethered particles^19,20^. These nanoswitch sensors have enabled the continuous monitoring of ssDNA^19^, small molecules^11–13,20–22^ (drugs, hormones, metabolites, neurotransmitters), and proteins^15,23,24^.

The majority of nanoswitch designs have focused on detecting analytes in micromolar and nanomolar concentration ranges, using binder molecules with relatively weak affinities and fast dissociation properties (*k*_off_) to achieve rapid sensor reversibility. For measuring analytes at very low concentrations (picomolar), a common viewpoint is that binder molecules are required with very high affinities, in order to effectively capture the analyte molecules from solution^15,25,26^. However, high-affinity binders are disadvantageous for continuous monitoring because the sensors relax very slowly towards equilibrium, caused by the strong retainment of analyte molecules by the binders. Various approaches have been proposed to improve the sensor kinetics, including pre-equilibrium measurements^25^, limited-volume sensing^27^, and active regeneration strategies^15,28^, in all cases resting on the notion that high-affinity binders are necessary to measure low-concentration analytes. Recently, we demonstrated the continuous monitoring of sub-nanomolar protein concentrations in a nanoswitch sensor with intrinsically reversible binder molecules, in other words, with reversibility solely based on passive dissociation^24^. This challenges the notion that high-affinity binders would be essential. The approach raises fundamental questions: can mathematical models be developed to understand and quantitatively describe how continuous nanoswitch-based biosensors operate? Can their analytical performance limits be predicted and will it be feasible to develop sensors that can continuously monitor even lower protein concentrations using intrinsically reversible binder molecules?

In this paper, we develop a rate-based simulation model to study the analytical performance of intrinsically reversible nanoswitch sensors. Using the model, we demonstrate that reversible, low-affinity interactions can be used to achieve precise, continuous monitoring of low-concentration biomolecules by using nanoswitches with single-molecule readout. Single-molecule readout resolves individual nanoswitches and their state-switching properties^29–34^, which is distinct from commonly used ensemble-averaged readout methodologies that accumulate signals from many nanoswitches without resolving individual nanoswitches or individual molecular interactions. We describe the operating principle of reversible nanoswitches and show how the accessible concentration range of the sensors can be engineered by varying the design parameters. The inherent variabilities arising from the stochastic nature of single-molecular events are clarified by Monte Carlo simulations. We assess the operating ranges of different sensor designs by calculating the limits where the analyte can be quantified with a concentration imprecision of 10%. With realistic design parameters, we predict that single-molecule readout of intrinsically reversible nanoswitches will allow the continuous monitoring of biomolecules at low-picomolar concentrations with response times on the order of 10 min.

## Results

**Fig. 1:**
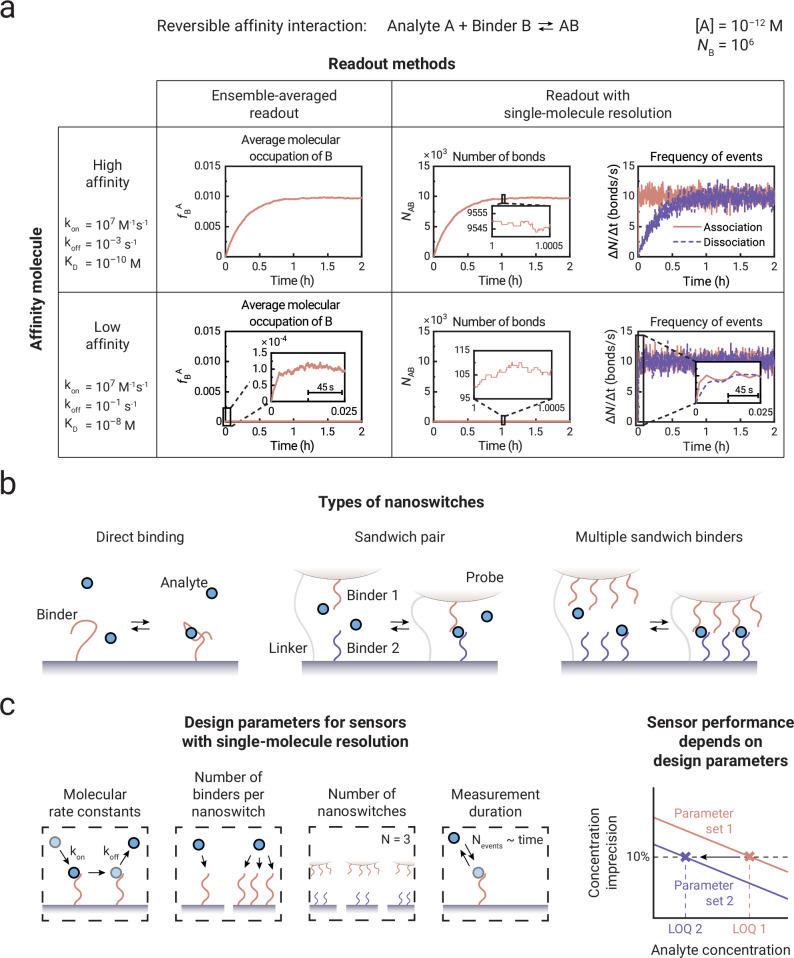
Affinity-based nanoswitch sensors for continuous monitoring. **a** Simulated kinetic response curves of sensors with ensemble-averaged readout and with single-molecule readout, for binders with strong and weak affinities. The sensors are modelled as *N*_B_ individual binder molecules B that capture analyte molecules A from solution, to form molecular complexes AB according to a Langmuir adsorption process. The kinetic rate constants are indicated in the panel. The plotted signal parameter of the ensemble-averaged sensor is *f*_B_^A^, the fractional occupancy of B by A. The sensor with single-molecule resolution is able to detect the formation of individual molecular bonds. The plotted signal parameters are the number of analyte-binder complexes (*N*_AB_) and the frequency of association and dissociation events (∆N/∆t). These are calculated using an integration time of 10 s. **b** Different types of affinity-based nanoswitches. A direct-binding nanoswitch uses a single binder molecule for analyte capture (left). A sandwich-pair nanoswitch uses a pair of binder molecules to capture the analyte molecule (middle). A nanoswitch with multiple sandwich binders is able to form multiple sandwich bonds (right). **c** Parameters for tuning the sensitivity of sandwich-type nanoswitch sensors with single-molecule resolution. The panel on the right sketches sensor precision profiles, i.e., the imprecision of the concentration measurement as a function of the analyte concentration. The sensitivity of a sensor is quantified as the limit-of-quantification (LOQ), i.e., the concentration at which the analyte concentration is determined with a measurement imprecision of 10%.

Figure 1 explains the rationale behind the concept of biomolecular sensing using low-affinity interactions combined with single-molecule readout. To focus on the basic concept, a simple model is used that includes only the capture of analyte molecules: analyte molecules A bind to binder molecules B, forming complexes AB. Using a Monte Carlo simulation, the binding of analyte molecules to binder molecules was simulated as a function of time, for a sensor with *N*_B_ binder molecules (*N*_B_ = 10^6^) that were exposed, starting at time *t* = 0, to a constant analyte concentration [A] = 1 pM. The simulation treats only the analyte binding process; it neglects any mass transport effects^35^ and it neglects the transduction of analyte binding into measurable signals^36^. Simulation details are given in Supplementary Note 1.

The simulation was performed for high-affinity and for low-affinity binders, having equal *k*_on_ values (10^7^ M^−1^s^−1^) and different *k*_off_ values (10^−3^ s^−1^ and 10^−1^ s^−1^, respectively). In all cases, the analyte concentration is orders of magnitude lower than the equilibrium dissociation constant of the affinity interactions ([A] « *K*_D_), which implies that the fractional occupancy of the binder molecules is always much smaller than unity, so most of the binder molecules have not captured any analyte molecule.

Figure 1a shows in a 2 × 2 matrix the simulated time-dependent responses of different sensor designs: sensors with ensemble-averaged readout (left column), sensors having readout with single-molecule resolution (right column), sensors with high-affinity binders (top row), and sensors with low-affinity binders (bottom row). The top-left curve represents the most commonly used sensor design for measuring low-concentration biomolecules: using high-affinity binders and ensemble-averaged readout. Ensemble-averaged readout methods record the average fractional occupancy of binder molecules by analyte molecules (*f*_B_^A^ = *N*_AB_/*N*_B_). The curve of the fractional occupancy as a function of time approaches equilibrium with a characteristic timescale of 1/*k*_off_ = 10^3^ s. The equilibrium fractional occupancy equals 0.01, determined by the magnitude of the analyte concentration with respect to the *K*_D_ of the interaction (*f*_B_^A^ = [A]/([A] + *K*_D_) = 0.01).

The bottom-left curve shows a sensor with low-affinity binders and ensemble-averaged readout. The sensor responds fast (1/*k*_off_ = 10^1^ s), but in equilibrium it reaches a very low fractional occupancy due to the low affinity of the binder molecules (*f*_B_^A^ = [A]/([A] + *K*_D_) = 10^−4^). The low fractional occupancy makes this design disadvantageous for measuring low-concentration analytes.

The responses with single-molecule readout are shown in the column on the right. Sensors with single-molecule resolution are comprised of many individual transducers that generate a signal upon each binding event, i.e., they are able to detect if and when individual AB complexes appear and disappear in the sensor. This means that the sensor can count the number of AB complexes and—more importantly—is able to record the frequency of appearance (association events) and of disappearance (dissociation events) of AB complexes. This is in contrast to ensemble-averaged readout methods, which have signal-integration strategies that cannot capture the discrete binding of individual analyte molecules.

The time dependencies of the main parameters are shown in the graph: the number of AB complexes (*N*_AB_) and the frequency of events (Δ*N*/Δt) for both association and dissociation. The *N*_AB_ curves of the single-molecule sensors have the same overall shape as the response curves of the sensors with ensemble-averaged readout, with one important difference: the curves of single-molecule sensors reveal individual steps, corresponding to the moments when individual AB complexes are formed or fall apart. The frequency-of-events data is shown on the right, for the association and the dissociation events. Initially, from *t* = 0 onwards, only association events are present, and after a certain time delay, also dissociation events appear. The delay of the dissociation signal with respect to the association depends on the dissociation rate constant of the binder molecules: for the high-affinity binders, the delay time equals 1/*k*_off_ = 10^3^ s, whereas for the low-affinity binders, the delay time equals 1/*k*_off_ = 10^1^ s.

An interesting property of the sensor with single-molecule resolution is that the frequency of association events responds instantaneously to the change of analyte concentration, independent of the dissociation rate constant of the analyte-binder interaction (note that mass transport, physical transduction, and signal processing will still cause non-zero response times in a practical biosensor). When the binder molecules are exposed to an analyte concentration of 1 pM, the frequency of association events becomes equal to *k*_on_ ∙ *N*_B_ ∙ [A] = 10 s^−1^. The value of this association rate is equal for the high-affinity and the low-affinity binders, because the binder molecules have equal *k*_on_ values (10^7^ M^−1^s^−1^). The dissociation rate constants of the binders are very different (*k*_off_ = 10^−3^ s^−1^ for the high-affinity binders; *k*_off_ = 10^−1^ s^−1^ for the low-affinity binders), but this difference does not affect the frequency of association events. This means that binder molecules having high *k*_off_’s, i.e., binder molecules with low affinities, can be suited for measuring low-concentration analytes. This is in stark contrast with the classical notion that low-concentration analytes can only be measured with high-affinity binder molecules^15,25,26^.

Another characteristic of binder molecules with low affinities is that they have a favorable impact on the mass transport requirements of the sensor. Operating in a regime where [A] « *K*_D_, the fractional occupancy *f*_B_^A^ scales inversely with the *K*_D_, so with low-affinity binders (high *k*_off_, high *K*_D_), the equilibrium number of AB complexes in the sensor is low. Therefore, when the analyte concentration changes, only very little net exchange of molecules is needed between the fluid and the sensing surface to allow the reaction to reach equilibrium. This loosens the size and transport requirements of the sensor, such as the required fluid volume and the required time for mass transport (see Supplementary Note 3.1).

In practice, an upper limit for the dissociation rate constant of the binder molecules is given by the integration time required for single-molecule detection. Readout with single-molecule resolution requires that molecular AB complexes remain bound during a sufficiently long time to allow the detection system to recognize and reliably resolve the individual AB complexes. The integration time depends on the physical single-molecule detection principle used in the sensor. The integration time defines the required bound state lifetime of the molecules and therefore sets an upper limit for the dissociation rate constant of the binder molecules.

In summary, continuous monitoring sensors with single-molecule resolution have different properties than sensors with ensemble-averaged readout. In case of simple molecular binding (A + B$\rightleftarrows$AB), the main single-molecule readout parameter is the frequency of association events, which responds instantaneously to changes in the analyte concentration, not limited by the dissociation rate constant of the binder molecules. When binder molecules with high *k*_off_ are used, the reaction equilibrates on short timescales and mass transport requirements are low. Ideally, binder molecules are used with high *k*_on_ and high *k*_off_, where the latter is limited by the integration time of the single-molecule detection principle used in the sensor.

To assess the analytical performance of nanoswitch sensors with single-molecule readout, we need to quantify the achievable precision of concentration measurements. Single-molecule sensors operate as a Poisson process with time-distributed digital events, in which the relative variabilities are expected to decrease with increasing number statistics. In the subsequent sections, we will discuss nanoswitch designs in relation to single-molecule detection, study dose-response characteristics for different sensor design parameters, and evaluate the precision and range of concentration measurements that can realistically be achieved with continuous nanoswitch sensors with single-molecule readout.

### Operating principles of nanoswitches

Reversible nanoswitches are constructs that include on the one hand a binding principle to capture analyte molecules and on the other hand a transduction principle to translate the binding into a physically observable signal via a switching event (Fig. 1b). For example, a nanoswitch can be a redox-labelled oligonucleotide that changes its redox current properties upon binding of an analyte molecule^11,15^, or a fluorescently labelled oligonucleotide that changes its fluorescence properties^13^, or a particle that changes its motion properties^19,20^. Nanoswitch designs can involve different binding configurations, e.g., single binding to a single analyte molecule (single binder, see Fig. 1b left panel), double binding to a single analyte molecule (binder 1 and binder 2, forming a sandwich pair, mid panel), or multiple binding to multiple analyte molecules (multiple sandwich pairs, right panel). Sandwich-type nanoswitches employ binders on two sides, generating a detectable signal when at least one full sandwich complex is formed with an analyte molecule. Sandwich nanoswitches have been demonstrated with transduction based on Förster resonance energy transfer^18,37^, redox current^17^, and particle motion^19,20^. In this work, we focus on sandwich-type nanoswitch sensors with multiple binders, for several reasons: (i) molecular sandwich formation is a flexible and generic principle for signal generation, (ii) the twofold affinity binding to analyte molecules gives opportunities to achieve high specificity and high sensitivity, and (iii) the ability to tune the number of binders in the nanoswitch gives opportunities to improve the analytical performance of the sensor. We will study several design parameters of sandwich-based nanoswitch sensors and determine their influence on the analytical performance of the sensors (Fig. 1c).

**Fig. 2:**
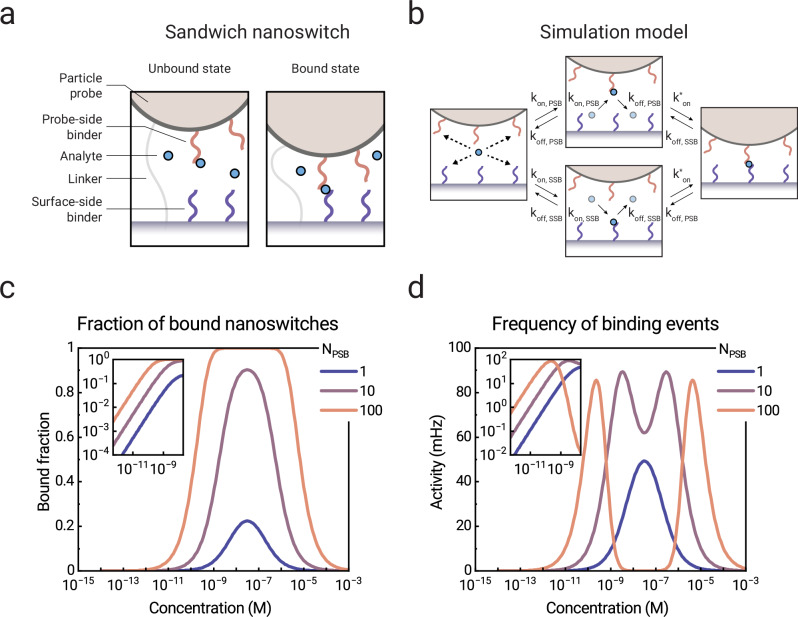
Rate-based nanoswitch model (RNM) of reversible sandwich nanoswitches with multiple binder molecules. **a** Sketch of a sandwich nano-switch with a particle probe. The particle probe is tethered to the surface via a flexible linker molecule. Both particle and surface are functionalized with affinity binders that can reversibly interact with the analyte in solution. The particle undergoes a change from a state with high motional freedom (unbound) to a state with low motional freedom (bound) upon the formation of a sandwich bond. The molecular bonds between analyte molecules and binder molecules are short-lived, causing the particle to switch between bound and unbound states. **b** Reaction pathways in the RNM simulation. Probe-side binders (PSB) and surface-side binders (SSB) interact independently with the analyte in solution, according to binder-specific kinetic rate constants. Upon binding of an analyte molecule, a sandwich bond can be formed according to an effective association rate k^*^_on_. The sandwich bond can break according to the dissociation rate constants of both binder molecules. **c** Response of the sandwich-based nanoswitch, plotted as the probability that the nanoswitch is in the bound state (bound fraction) as a function of the analyte concentration, for different numbers of binders on the probe (*N*_PSB_). The bound fraction was calculated analytically, as described in Supplementary Note 4. The inset shows the low-concentration data plotted on log–log scales. **d** Response of the sandwich nano-switch, plotted as the switching activity (the frequency of switching events between bound and unbound states of the nanoswitch) as a function of the analyte concentration, for different numbers of binders on the probe (*N*_PSB_). The activity was calculated analytically, as described in Supplementary Note 4. The inset shows the low-concentration data plotted on log–log scales.

To investigate the analytical performance, we developed a rate-based nanoswitch model (RNM) that could be solved analytically and was also implemented in a Monte Carlo simulation, see Fig. 2a, b. The model is designed to be independent of the transduction mechanism to detect the state of the nanoswitch and is therefore applicable to nanoswitches with various detection methods, such as using redox probes, fluorescent probes, or particles. In this study, simulation results were compared to experimental results based on Biosensing by Particle Motion (BPM), where particles are the probes that reflect by their motion properties if molecular sandwich bonds are formed in the nano-switches, i.e., sandwich bonds formed between particle and surface^19^. The binder molecules on the probe-side of the nanoswitch are called the probe-side binders (PSB), and on the other side, the surface-side binders (SSB). In absence of a sandwich bond, a nano-switch is in its unbound state (open state). Sandwich formation causes the nanoswitch to enter its bound state (closed state), with a reduced motional freedom of the particle. The motion properties of many particles are continuously tracked by video microscopy. Switching events between bound and unbound states are recognized and analyzed, resulting in a state-switching readout parameter that relates to the analyte concentration in solution. More details on the BPM sensing methodology can be found in Supplementary Note 2.

The RNM treats a nanoswitch as two parts that interact with each other, each part functionalized with a discrete number of binder molecules (*N*_PSB_ and *N*_SSB_), see Fig. 2b. Binder molecules capture analyte molecules from solution and also interact with analyte molecules bound on the opposite side. Capture from solution is described with association and dissociation rate constants *k*_on_ (M^−1^s^−1^) and *k*_off_ (s^−1^), respectively. When an analyte molecule is captured on one side, it allows for the formation of a sandwich bond by interacting with binders on the opposite side. For simplicity, the intra-nanoswitch sandwich bond formation rates are assumed to be equal for both sides, having the same effective association rate constant k^*^_on_ (s^−1^)^34,38^. A sandwich bond falls apart if one of the two bonds dissociates, according to their respective dissociation rate constants.

In the present model, all molecular interactions are assumed to be independent: interactions are either possible or not possible, and the rates are fixed. This is a simplification, because molecular interactions can in principle, influence each other. For example, when a first sandwich bond is formed in a nanoswitch (monovalent bond), it may positively or negatively affect the formation of subsequent sandwich bonds (cooperative and anti-cooperative effects, in case of multivalent bonds). However, the magnitudes of these effects are difficult to predict and tested model descriptions are not yet available. Furthermore, this paper aims to estimate limits of quantification, which means that the simulations focus mainly on conditions with low analyte concentrations, where the behavior of nanoswitches is dominated by monovalent rather than multivalent binding. The present model includes monovalent as well as multivalent nanoswitch states, but the used rates do not yet include potential cooperative and anti-cooperative effects. More information on this topic can be found in Supplementary Note 4.7.

To explore the dynamic behavior of sandwich-type nano-switches, we modelled the sensors using the parameter values listed in Table 1. Because *k*_on_ values of macromolecular affinity reactions lie typically in the range of 10^5^–10^7^ M^−1^s^−1^ ^39,40^, we assumed *k*_on_ = 10^6^ M^−1^s^−1^. *K*_D_ values of commercially available antibodies used in ELISA assays have equilibrium dissociation constants *K*_D_ typically in the nanomolar range or lower^41,42^, corresponding to *k*_off_ values in the order of 10^−3^ s^−1^ or less (*K*_D_ = *k*_off_/*k*_on_). For low-affinity binders, we assumed *k*_off_ values in the range of 10^−2^–10^−1^ s^−1^ ^23^, corresponding to mean lifetimes of 10–100 s, suitable for several single-molecule readout principles^43^ including BPM^19^. Sandwich-type biosensors typically target two distinct epitopes on the analyte molecule, using two distinct binder molecules with different affinities; this is reflected in Table 1 by the different *k*_off_ values of the probe-side and SSB. The intra-nanoswitch sandwich formation rate k^*^_on_ has not yet been accurately studied in literature; we based a first estimate on experimental BPM data (see Supplementary Note 3.2). The number of sandwich-forming binder molecules in nanoswitches can vary from a few up to several hundreds^17,18,44^. The standard number used in the simulations was 10 binders on each side of the nanoswitch. The standard parameter values are summarized in Table 1.

**Table 1:** Standard parameter values used in the RNM simulation

Parameter	Value	Description
*N*_PSB_	10	Number of probe-side binders
*N*_SSB_	10	Number of surface-side binders
*k*_on,PSB_	10^6^ M^−1^s^−1^	Association rate constant of probe-side binders
*k*_off,PSB_	10^−2^ s^−1^	Dissociation rate constant of probe-side binders
*k*_on,SSB_	10^6^ M^−1^s^−1^	Association rate constant of surface-side binders
*k*_off,SSB_	10^−1^ s^−1^	Dissociation rate constant of surface-side binders
k^*^_on_	5 ∙ 10^−3^ s^−1^	Intra-nanoswitch sandwich bond formation rate

Calculations were performed with the values given in this Table, unless explicitly stated otherwise.

### Nanoswitch responses for different design parameters

The RNM simulation describes a nanoswitch as a system that distributes over state fractions *F*_*i*_, where index $i$ represents the number of sandwich bonds present in the nanoswitch. *F*_0_ is the unbound nano-switch state fraction and *F*_1_, …, *F*_*n*_ are bound nanoswitch state fractions, with $n$ the maximum number of sandwich bonds formed in the nanoswitch. The transitions between state fractions are governed by state-dependent association and dissociation rates:

1 $${F}_{0}\begin{array}{c}{k}_{a}^{\left(0\right)}\\ \rightleftarrows \\ {k}_{d}\end{array}{F}_{1}\begin{array}{c}{k}_{a}^{\left(1\right)}\\ \rightleftarrows \\ {2\cdot k}_{d}\end{array}{F}_{2}\begin{array}{c}{k}_{a}^{\left(2\right)}\\ \rightleftarrows \\ {3\cdot k}_{d}\end{array}\ldots \begin{array}{c}{k}_{a}^{\left(n-1\right)}\\ \rightleftarrows \\ {n\cdot k}_{d}\end{array}{F}_{n}$$

The forward rates *k*_a_^(i)^ describe the transitions from states *F*_*i*_ to *F*_*i*+1_. The forward rates are modelled as products of the number of single-bound analyte molecules on one side, the number of free binder molecules on the other side, and the intra-nanoswitch sandwich formation rate k^*^_on_. The numbers of bound analyte molecules and free binder molecules are determined by the fractional occupancies multiplied by the number of binders, with subtraction of the number of sandwich bonds present. The reverse rates are modelled as the respective numbers of sandwich bonds multiplied by the rate of sandwich bond dissociation (*k*_d_ = *k*_off,PSB_ + *k*_off,SSB_). This scheme translates into a set of ordinary differential equations with the following generalized form:

2 $$\frac{d{F}_{i}}{{dt}}={k}_{d}\cdot \left({\left(i+1\right){\cdot F}_{i+1}-i\cdot F}_{i}\right)+{k}_{a}^{\left(i-1\right)}{\cdot F}_{i-1}-{k}_{a}^{\left(i\right)}{\cdot F}_{i}.$$

The underlying assumptions are discussed in more detail in Supplementary Note 3. The equations can be solved analytically for arbitrary $n$ value, for a steady-state condition with a constant analyte concentration in solution. A full derivation of the solution can be found in Supplementary Note 4.

The derived analytical expressions are used to calculate the properties of nanoswitches with multiple sandwich binders. The bound fraction parameter is the time fraction that a single nanoswitch is in a bound state, which is equivalent to the fraction of nanoswitches that are in a bound state at a given moment in time^45^:

3 $${Bound}\,{fraction}={\sum}_{i=1}^{n}{F}_{i}=1-{F}_{0}$$

Figure 2c shows the bound fraction as a function of the analyte concentration, for *N*_SSB_ = 10 and *N*_PSB_ ranging from 1 to 100. At low analyte concentrations (C « *k*_D_), the fractional occupancies of binder molecules are low, so sandwich bonds are rarely formed, and therefore the bound fraction is much smaller than unity. In this regime, the bound fraction scales linearly with the analyte concentration, as visualized in the inset with double-log axes. The bound fraction increases with increasing *N*_PSB_; thus for detecting low analyte concentrations, it is advantageous to provide a nanoswitch sensor with a high number of binder molecules.

As the analyte concentration increases, the probability of sandwich bond formation rises, leading to more nanoswitches being in the bound state. The maximum bound fraction is reached when the analyte concentration equals the geometric mean of the *K*_D_’s of the affinity binders ($\sqrt{{K}_{D,{PSB}}\cdot {K}_{D,{SSB}}}$ = 32 nM), independent of the number of binder molecules in the nanoswitch. For even higher analyte concentrations, the bound fraction decreases because binders in the nanoswitch become saturated with analyte, reducing the likelihood of sandwich bond formation, known in a sandwich assay as the high-dose hook effect.

A readout parameter that quantifies the switching rate of the nanoswitch is the switching activity^19^, defined as the frequency of transitions between unbound and bound nanoswitch states:

4 $${Activity}={k}_{a}^{\left(0\right)}\cdot {F}_{0}+{k}_{d}\cdot {F}_{1}$$

Figure 2d shows how the switching activity depends on the analyte concentration, using the same nanoswitch parameters as in Fig. 2c. For a nanoswitch with a single probe-side binder (*N*_PSB_ = 1, blue curve), the curve shape is very similar to the bound fraction: the switching activity increases with analyte concentration up to the geometric mean (32 nM) and then decreases due to the high-dose hook effect. The curves for higher *N*_PSB_ (10 and 100) have larger signal magnitudes because more analyte molecules are captured, but also show a distinct downward inflection around the geometric mean. Two peaks are now observed in the simulated dose-response curves: a first peak at low concentrations and a second peak at higher concentrations. The origin of these peaks can be understood from the fractional occupancy of the binders in the nanoswitch. As the analyte concentration increases from zero, the activity signal initially increases because of the formation of monovalent bonds between particle and surface (left side of the first peak), and subsequently decreases when multivalent bonds dominate the response (right side of the first peak). When the analyte concentration increases further, both probe-side and surface-side binders become increasingly occupied by analyte. The occupation on both sides reduces the probability that molecular sandwich bonds are formed between particle and surface, causing a transition from multivalent binding to monovalent binding, and therefore an increase of the activity signal (left side of the second peak). As the analyte concentration further increases, binders on both sides become fully saturated with analyte, causing a complete loss of sandwich bonds and a decrease of the activity signal to zero (right side of the second peak). More information about the peaks and transitions can be found in Supplementary Note 4.4.

**Fig. 3:**
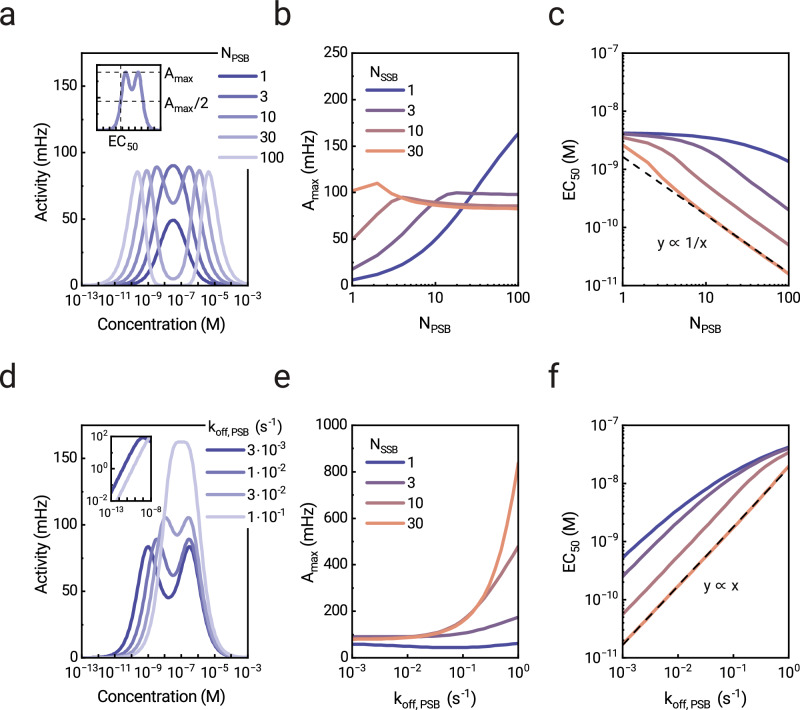
Response characteristics of sandwich nanoswitches with varying numbers of binder molecules and dissociation rate constants. **a** Activity-versus-concentration curves are characterized by two parameters: A_max_ and EC_50_. A_max_ indicates the maximum response of the sensor. EC_50_ corresponds to the lowest concentration at which the signal reaches half of A_max_. **b** Dependence of A_max_ on *N*_PSB_, plotted for different numbers of binders on the surface (N_SSB_). **c** Dependence of EC_50_ on *N*_PSB_. The line colors relate to N_SSB_ values indicated in the legend of (**b**). The EC_50_ is shown to scale with the reciprocal of *N*_PSB_ (black dashed line). **d** Activity-versus-concentration curves for different values of the dissociation rate constant of the probe-side binder *k*_off,PSB_. The inset shows response curves on log–log scales, for *k*_off,SSB_ = 3 ∙ 10^−3^ s^-1^ (dark blue) and *k*_off,SSB_ = 1 ∙ 10^−1^ s^−1^ (light blue). **e** Dependence of A_max_ on *k*_off,PSB_, for different values of N_SSB_. *k*_off,SSB_ was constant at 10^−1^ s^−1^. **f** Dependence of EC_50_ on *k*_off,PSB_. The line colors relate to N_SSB_ values indicated in the legend of (**e**). The EC_50_ is shown to scale linearly with *k*_off,PSB_ (black dashed line). *k*_off,SSB_ was constant at 10^−1^ s^−1^.

The nanoswitch response properties are further explored in Fig. 3 for two main design parameters: the number of binder molecules and the magnitude of dissociation rate constants. Figure 3a shows that an increase of *N*_PSB_ increases the width of the response curves and changes the depth of the inflections, but hardly changes the maximum value of the signals. The maximum value corresponds to a condition where the effective association rate of the probe to the surface is equal to the dissociation rate (see Supplementary Note 4.4), which also marks the transition from monovalent to multivalent binding in the nanoswitch (see Supplementary Fig. 5). From these response curves, the maximum switching activity signal (A_max_) and the effective concentration at which the signal equals 50% of the maximum signal (EC_50_) can be quantified, as indicated in the inset. Figure 3b, c show the values of A_max_ and EC_50_ for nanoswitches with both *N*_PSB_ and *N*_SSB_ varied. For a nanoswitch with only a single binder on the surface (*N*_SSB_ = 1), A_max_ increases with increasing *N*_PSB_. For higher *N*_SSB_ values, A_max_ increases with increasing *N*_PSB_ up until a saturation level, which is the maximum attainable activity where monovalent binding dominates the nanoswitch response. Figure 3c shows that the EC_50_ decreases with increasing numbers of probe-side as well as surface-side binders, both with an inverse relationship (1/*N*). This behavior arises because increasing the number of binders linearly increases the probability that an analyte molecule is captured within a nanoswitch, thereby increasing the number of switching events. This demonstrates that varying the number of binders is a convenient method to tune the sensitivity range of the nanoswitch sensor. Experimentally, the inverse scaling of the EC_50_ with the number of binders was investigated in a sandwich-type BPM sensor for monitoring single-stranded DNA (see Supplementary Note 4.6). The EC_50_ was observed to decrease with an increasing incubation concentration of PSB, in agreement with the behavior seen in the simulations (Supplementary Fig. 9).

Figure 3d shows how the sensor response depends on the dissociation rate constant of the probe-side binder (*k*_off, PSB_), which is the binder molecule with a slower dissociation compared to the surface-side binder (*k*_off, SSB_ = 0.1 s^−1^). An increase of its dissociation rate constant causes the overall response curve to shift toward higher concentrations, due to the reduced affinity of the binder. Also, the downward inflection disappears due to dominance of monovalent binding compared to multivalent binding. Figure 3e, f show how A_max_ and EC_50_ depend on *k*_off, PSB_, calculated for different values of *N*_SSB_. For *N*_SSB_ = 1, the results show that the maximum signal changes little with different affinities of the probe-side binder. The reason is that the response curves shift to higher concentrations (higher EC_50_) without substantially changing their shapes. When both dissociation rate constants are equal (0.1 s^−1^), the least number of switches is obtained per bound analyte, resulting in the minimum of A_max_. Nanoswitches with *N*_SSB_ > 1 show very different behaviors, caused by the possibility to form multivalent bonds between probe and surface. A steep increase of A_max_ is observed when the *k*_off, PSB_ is increased (see Fig. 3e, for *N*_SSB_ > 1), as this causes the nanoswitch to be dominated by monovalent rather than multivalent bonds. This highlights the importance of tuning the number and the affinity of the binder molecules in the nanoswitch.

To summarize, the results of this Section show how the signals of reversible nanoswitches and the accessible concentration range can be tuned by changing design parameters such as the number of binder molecules per nanoswitch and their rate constants. Next, we will investigate how the analytical properties of sensors with single-molecule resolution depend on the signal acquisition parameters.

### How the stochastics of single-molecular events limit the sensor precision

A practical nanoswitch biosensor with single-molecule readout consists of a large number of individual nanoswitches, of which the digital switching properties are continuously monitored at the single-nanoswitch level. The signals of the individual nanoswitches are different because they have intrinsic heterogeneities. A fundamental source of variability is the stochastic nature of molecular (un)binding events, causing different nanoswitches to have different signals as a function of time. A source of inter-nanoswitch variabilities is the nano-fabrication process that can result in a spread in the number of binder molecules per nanoswitch.

In this Section, we address the question how the concentration measurement uncertainty of a nanoswitch sensor is influenced by heterogeneities related to (1) the stochastics of molecular binding and switching processes in individual nanoswitches as well as (2) stochastics in the numbers of binder molecules per nanoswitch (see Supplementary Note 4.8). We developed a Monte Carlo simulation model that describes the molecular binding and unbinding events as independent Poisson processes with exponentially distributed lifetimes, described by the rate constants shown in Fig. 2b. Furthermore, the numbers of binders per nanoswitch were sampled from Poisson distributions with means equal to *N*_PSB_ and *N*_SSB_, reflecting random incorporations of binder molecules in a nanoswitch^34^. A full description of the model is given in Supplementary Note 5.

**Fig. 4:**
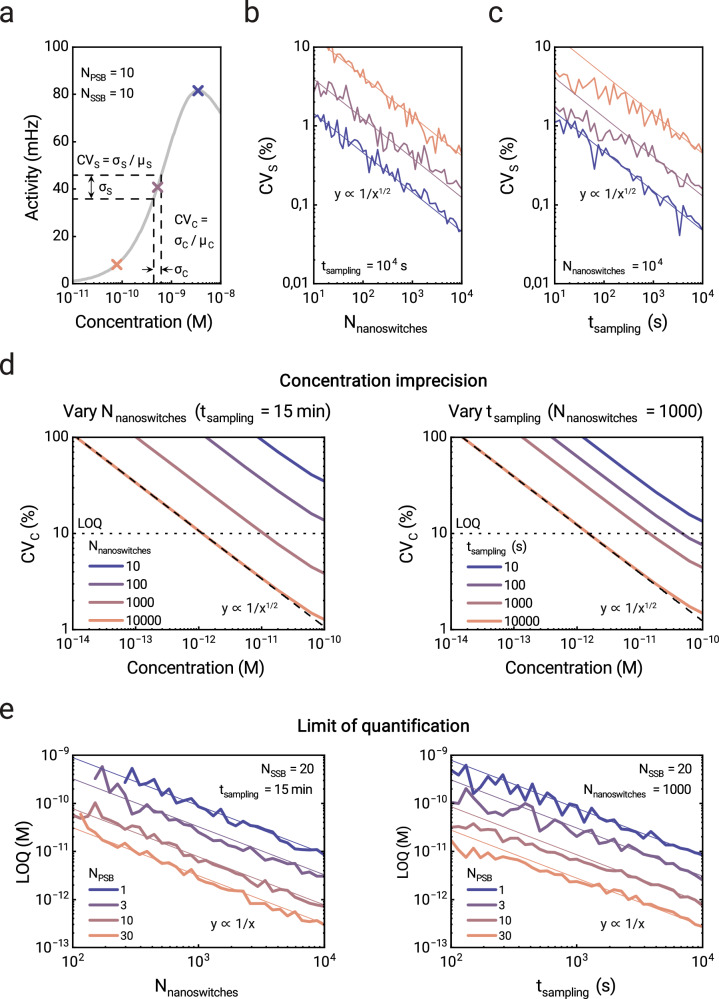
Analytical performance of nanoswitch sensors for different design parameters. **a** Method to calculate the concentration imprecision of a nanoswitch sensor. The grey curve represents the dose-response curve, constructed using the mean of the sampling distribution as a function of analyte concentration. The coefficient of variation of the sensor signal (CV_S_) is obtained by calculating the standard deviation of the sampling distribution (σ_S_) divided by the mean of the sampling distribution (µ_S_). The coefficient of variation of the concentration determination (CV_C_ = σ_C_/µ_C_) is calculated using CV_S_ and the slope of the fit of the dose-response curve (dS/dC, see Supplementary Note 6.1). The three crosses indicate a low concentration, the EC_50_, and the concentration at A_max_, which are further investigated in (**b** and **c**). **b** Simulated signal imprecision (CV_S_) as a function of the number of nanoswitches (*N*_nanoswitches_). The colors of the curves correspond to the crosses in (**a**). The lines are guides to the eye. **c** Simulated signal imprecision (CV_S_) as function of the sampling time. The colors of the curves correspond to the crosses in (**a**). **d** Simulated concentration imprecision (CV_C_) as function of the analyte concentration, calculated for different numbers of nanoswitches (left) and signal sampling times (right). **e** Simulated limit-of-quantification as a function of different numbers of nanoswitches (left) and signal sampling times (right), plotted for different *N*_PSB_.

The imprecision of the analyte concentration measurement is quantified by its coefficient of variation (CV_C_), which is the ratio between the standard deviation *σ*_C_ and the arithmetic mean *μ*_C_ of the sampling distribution of the concentration: CV_C_ = *σ*_C_/*μ*_C_. The relative concentration imprecision CV_C_ is related to the relative imprecision of the sensor signal (CV_S_) via the calibration curve of the sensor, as indicated in Fig. 4a. In that way, a measured sensor signal and its variability translate into a measured analyte concentration and its concentration variability. This is explained in more detail in Supplementary Note 6.1.

Theoretically, the imprecision of the underlying Poisson processes should approach zero as more and more switching events are counted, by either increasing the number of nanoswitches (*N*_nanoswitches_) or the total sampling time of the measurement signal (*t*_sampling_). The total sampling time is the measurement time window used to count switching events and calculate the switching activity. The scaling relationships are investigated in Fig. 4b, c, for nano-switch sensors with identical properties. The plotted curves represent simulation results for three analyte concentrations: well below the EC_50_ (70 pM, orange), the EC_50_ concentration (700 pM, purple), and the concentration of maximum signal (3 nM, blue). The sensor signal represents the average of all nanoswitches. Each simulation was repeated 15 times to provide a precise estimation of the sampling distribution and to calculate *σ*_S_ and *μ*_S_, the standard deviation and mean of the sensor signal, respectively. The scatter in the CV_S_ data relates to the imprecisions in the standard deviation and mean values themselves (see Supplementary Note 6.2). Figure 4b shows that the signal imprecision CV_S_ decreases with increasing analyte concentration, caused by the increased number of switches. The CV_S_ scales inversely with the square root of the number of nano-switches ($\propto$1/$\sqrt{{N}_{{nanoswitches}}}$) and with the total signal sampling time ($\propto$1/$\sqrt{{t}_{{sampling}}}$), in accordance with Poisson statistics. For low analyte concentrations, the data deviates from a 1/$\sqrt{{t}_{{sampling}}}$ behavior, in the regime where *t*_sampling_ is smaller than the slowest dissociation time (1/*k*_off, PSB_ = 100 s). In this regime, *σ*_S_ is dominated by rare binding events, leading to an underestimation of the variability^46^; more information is given in Supplementary Note 6.3.

To validate the simulation results, we analyzed experimental data of a sandwich-type BPM sensor for monitoring single-stranded DNA (see Supplementary Note 6.4). Switching signals were measured for an analyte concentration near the EC_50_ (750 pM). Measurements were performed 10 times with a sampling time of 15 min per measurement, yielding distributions of activity values similar to the simulated data. When the experimental data was corrected for time-dependent drift, the signal imprecision of the BPM sensor follows the scaling laws expected from a Poisson-limited sensor (see Supplementary Figs. 19 and 21).

In summary, we explored in this Section how stochastic variations in nanoswitches cause fluctuations in the measurement signals. In the following Section, we will discuss how this translates into a concentration measurement uncertainty and how the variabilities affect the quantification limits of nanoswitch sensors with single-molecule resolution.

### Achievable sensitivity of reversible nanoswitches with single-molecule resolution

Using the signal imprecision CV_S_ discussed in the previous Section, we can now study how the concentration imprecision CV_C_ depends on the design and signal acquisition parameters of a nanoswitch sensor. Figure 4d shows the concentration measurement imprecision as a function of the analyte concentration, the number of nanoswitches, and the signal sampling time, for nanoswitches with on average 10 binders on the probe side and 10 binders on the surface side. The CV_C_ was calculated as indicated in Fig. 4a, using *σ*_S_ derived from fitted $\sqrt{x}$ lines as described in Supplementary Note 6.5.

The curves in Fig. 4d scale with concentration as 1/$\sqrt{C}$, in agreement with Poisson statistics, with a slight upward bending for concentrations approaching the EC_50_ (see Supplementary Note 6.1, Eq. (6.6)). In the left panel, the lines are spaced evenly apart, reflecting the scaling with 1/$\sqrt{{N}_{{nanoswitches}}}$ that was also observed in Fig. 4b. In the right panel, the lines are not spaced evenly apart, caused by the bivariate exponential waiting time, as explained in Supplementary Note 6.3.

The limit-of-quantification (LOQ) of a sensor is the analyte concentration where the CV_C_ equals 10%, i.e., the condition where the analyte concentration can be measured with an imprecision of 10%. This analytical performance level is marked by the dotted horizontal lines in Fig. 4d. The points where the dotted lines cross the CV_C_ curves represent the LOQ concentration values for the corresponding values of *N*_nanoswitches_ and *t*_sampling_. The resulting LOQ values are plotted in Fig. 4e for different numbers of nanoswitches, signal sampling times, and different numbers of binder molecules (*N*_SSB_ = 20, *N*_PSB_ ranges between 1 and 30). The results show that the LOQ scales inversely with all three parameters: the number of nanoswitches, the sampling time, and the number of binder molecules. These are all very convenient parameters for engineering nanoswitch sensors toward LOQ values that are desired for applications. Further details on the scaling laws of the LOQ are given in Supplementary Note 5.2 and Box 1 in Supplementary Note 7.2.

The results of Fig. 4e can be used to estimate the achievable LOQ of a continuous sandwich-type nanoswitch biosensor with intrinsically reversible binder molecules, using realistic parameters for the sensor design and signal acquisition. A realistic number of nanoswitches is 10^4^, because BPM sensors have been demonstrated already with more than 10,000 particles^36^. The signal sampling time *t*_sampling_ is a factor that influences the time delay of a continuous sensor^36^. The time delay is the difference between the timestamp at which a biomarker concentration is probed in a bio-system, and the delayed instant when the biomarker concentration is reported by the biosensor. For a practical sensor application, the delay time should be short enough to be acceptable for the intended application. More precisely, the delay should be short with respect to the acceptable time difference between a characteristic status change of the bio-system (e.g., a biomarker crossing a threshold concentration) and the intended consequential action (e.g., a change of a control parameter applied to the bio-system). As an example, an important medical application is the monitoring of patients who are at risk of and are treated for acute systemic inflammation^1,2^. For monitoring-and-control of these patients, sensing time delays on the order of 30 min are expected to be acceptable^1,2^. In industrial bioprocessing, decisions on process steps are typically taken on timescales of less than 1 h^23,24^, so time delays on the order of 15 min are expected to be acceptable for many industrial monitoring-and-control applications.

Estimations are also needed of the number of binder molecules on the probe side and on the surface side of the nanoswitch. Using a tethered BPM sensor as a reference, the numbers can be estimated from the interaction area of a spherical particle tethered to a flat surface, the areal binder densities on both sides, and their mutual accessibilities. The interaction area can be calculated from geometric considerations regarding the particle radius and the tether length (around 6.1 ∙ 10^4^ nm^2^)^47^. In the BPM experiments of this paper, the binder molecules were coupled non-covalently to biofunctionalized surfaces. The surface was coated with a polymer layer (PLL-g-PEG) functionalized with single-stranded DNA (ssDNA), to which ssDNA-functionalized binder molecules were hybridized, with an areal density of 10^4^ molecules per µm^2^ ^48^. The particles were coated with streptavidin to which biotin-functionalized binder molecules were attached, with a typical areal density of 10^3^–10^5^ molecules per µm^2^ ^49^. Finally, the ability of binder molecules to effectively contribute to the formation of molecular sandwiches between particle and surface depends on many aspects: the molecular orientations of the binders, molecular crowding effects, surface roughness^50,51^, the length and mechanical properties of the linker molecule^20,47^, etc. These combined aspects have not yet been studied in great detail; we assume that the mutual accessibility can range between a few percent and a few tens of percents^50,51^. Given these numbers, we assume that 10–30 is a realistic estimation for the numbers of sandwich-forming binder molecules in a tethered BPM nanoswitch.

The orange-colored curves in the left panel of Fig. 4e represents a sensor with 10^4^ probes, a signal sampling time of 15 min, having on average 20 binders on the probe-side and surface-side, with dissociation rates of *k*_off, PSB_ = 10^−2^ s^−1^ (*K*_D, PSB_ = 10 nM) and *k*_off, SSB_ = 10^−1^ s^−1^ (*K*_D, SSB_ = 100 nM) (see Table 1). The simulation data in Fig. 4e show that with these input parameters, nanoswitch sensors can be expected with limits of quantification of around one picomolar. This leads to the conclusion that intrinsically reversible sandwich-based nanoswitch sensors, using readout with single-molecule resolution, will allow the measurement of biomolecular concentrations in the low picomolar concentration range, with a concentration measurement precision of 10% and a delay time of around 15 min.

## Discussion

Monitoring-and-control strategies applied to dynamic biosystems will require continuous biomolecular sensors that are both sensitive and fast. Here, we investigated the sensitivity and speed characteristics of reversible nanoswitch sensors with single-molecule readout. We focused on sandwich-type nanoswitch sensors with multiple binders and developed a rate-based model that could be solved analytically as well as implemented in a Monte Carlo simulation. Results were compared to experimental data of particle-based nanoswitches with single-molecule resolution.

We studied how the sensor response characteristics and the concentration measurement precision depend on molecular design parameters (kinetic rates, numbers of binders molecules) and signal acquisition parameters (number of nanoswitches, signal sampling time). The results show that the concentration measurement precision is limited by the counting statistics of molecular sandwich association and dissociation events. Using realistic design parameters, we predict that limits-of-quantification in the low picomolar range can be achieved with signal sampling times in the range of minutes.

Reversible nanoswitch sensors enable continuous monitoring without requiring any wash steps or renewal of affinity reagents, making the sensing concepts suited for long-term operation. In literature, reversible nanoswitch sensors have mostly been combined with ensemble-averaged electrochemical^11^ and fluorescence readouts^12,13^, showing sensitivities in the micromolar to nanomolar ranges. To measure even lower concentrations, the general consensus is that high-affinity interactions are required to capture and retain a sufficient number of analyte molecules in the sensor in order to create measurable signals^15,25,26^. However, high-affinity interactions cause long equilibration times and slow sensor responses, which hinder the use for continuous monitoring. Active dissociation strategies are being explored^15,28^, but these require extra steps that may introduce sources of variability in the sensing process. In this paper, we have challenged the notion that high-affinity binders are essential. By combining intrinsically reversible binders with a single-molecule readout, it becomes possible to monitor low-concentration analytes with fast response times and high concentration measurement precisions. The principles have been illustrated in this paper using simulations, supported by experimental results of a particle-based nanoswitch. We foresee that this can become a generalizable approach for realizing continuous biomolecular monitoring with single-molecule detection methods^18,43,52^, provided that single-molecule switching events are continuously measurable with sufficient signal-to-noise ratio over long durations.

The theoretical framework developed in this paper describes a nanoswitch sensor as a system consisting of two interacting parts that have discrete numbers of binder molecules, with molecular interaction rates according to Poisson point processes with exponentially distributed waiting times. In literature, biosensors have been modelled using analytically solved Langmuir models^25^, stochastic Markov chain models^53^, thermodynamic binding models^54^, Langevin equation-based models^55^, and molecular dynamics^15^, for example. We have previously developed models of particle-based sensors using analytical Langmuir-type equations^19^, Brownian dynamics simulations^38,45^, and Monte Carlo models^20,34,56^, for elucidating specific biophysical aspects of particle-based biosensing. The RNM described in this paper has been designed to enable calculations of the concentration measurement imprecision. With the analytical solutions and Monte Carlo implementation, the model can be used to answer questions about the influence of sensor design parameters and acquisition parameters on the analytical performance of the sensors. We expect that the developed modelling framework has the potential to become broadly useful for interpreting measurement data, for directing experimental approaches, including experiments to extract parameter values, and for designing new nanoswitch-based continuous sensors.

To improve the simulation model, further studies may be directed toward including geometrical effects, as these can influence analyte capture rates, sandwich formation rates, and multivalent binding dynamics. In its current form, the RNM simulation neglects potential cooperative and anti-cooperative effects by assuming that all molecular interactions are independent. Cooperative effects may enhance effective sandwich formation rates, as the formation of an initial bond brings the probe and surface into closer proximity. Conversely, anti-cooperative effects could reduce effective formation rates due to decreased translational and rotational freedom of the probe after the first bond forms, particularly when binders are not optimally aligned or oriented. Experimental studies and simulations (Brownian or molecular dynamics) would be valuable to quantify the magnitudes of these effects, followed by implementation of the effects in the rate-based model, for example, by including a dependence of rate parameters (k^*^_on_, *k*_on_, or *k*_off_) on the number of sandwich bonds formed.

It will also be important to include non-specific interactions in the model. In a preliminary approach, we have added non-specific interactions between probe and surface as a two-state Poisson process that operates in parallel to the specific interactions, see Supplementary Note 7. The results show that suppressing BPM background signals to approximately 2 mHz can already enable LOQs in the picomolar range using the standard parameters in this paper, providing a clear target for experimental strategies to suppress non-specific interactions, such as the development of low-fouling and blocking layers.

We have presented a methodology to understand and predict analytical performance parameters of intrinsically reversible nanoswitch sensors measured with single-molecule resolution. We predict that quantification limits of around one picomolar are achievable with response times of minutes. We believe that these results will accelerate the development of continuous biomolecular sensors with high sensitivity and high speed, to the benefit of fundamental biological research and for monitoring-and-control applications in healthcare, industrial bioprocessing, and environmental safety.

## Methods

### Materials

Custom-made cyclic olefin copolymer (COC) cartridges containing a flow chamber with a volume of 20 μL and a chamber height of 250 μm were produced by Axxicon using injection molding. These cartridges were designed to be compatible with the custom-built automated setup used in the experiments. All oligonucleotide sequences were purchased from Integrated DNA Technologies; the specific sequences used are listed in Table 2.

**Table 2:** DNA sequences used in this work

	Sequence
**dsDNA tether**	5′ DBCO – GGT TAG CAG CCT GTT TCA AAA CCT GGG GGT GAG TGT CAC GCC AAT TCA GCG CAT CGT TCT GTC GGG AGA GAA TGG TCT GAA AAT CGA TAT CCA CGT CAT TAT CCC GTA CGA AGG TCT TTC TGG TGA TCA GAT GGG GCA GAT AGA AAA AAT ATT CAA AGT GGT GTA CCC AGT AGA CGA TCA TCA CTT CAA GGT TAT ACT GCA CTA TGG CAC CCT CGT TAT CG 3′ 3′ CCA ATC GTC GGA CAA AGT TTT GGA CCC CCA CTC ACA GTG CGG TTA AGT CGC GTA GCA AGA CAG CCC TCT CTT ACC AGA CTT TTA GCT ATA GGT GCA GTA ATA GGG CAT GCT TCC AGA AAG ACC ACT AGT CTA CCC CGT CTA TCT TTT TTA TAA GTT TCA CCA CAT GGG TCA TCT GCT AGT AGT GAA GTT CCA ATA TGA CGT GAT ACC GTG GGA GCA ATA GC – Biotin 5′
**Particle-side binder (PSB)**	5′ TCA CGG TAC GAT TTT TTT TTT – Biotin 3′
**Surface-side binder (SSB)**	5′ GCA GTC ACG TTC TCG AAT GCA ACA TTA TTA C 3′
**DBCO-docking ssDNA**	5′ CGA TTC GAG AAC GTG ACT GCT TTT T – DBCO 3′
**Target 22nt**	5′ TTG TAC CGT GAG TAA TAA TCC G 3′
**Biotin-polyT**	5′ TTT TTT TTT TTT TTT T – Biotin 3′

Phosphate-buffered saline (PBS) tablets, sodium chloride (NaCl), Tween-20, and bovine serum albumin (BSA) were obtained from Sigma-Aldrich. Streptavidin-coated Dynabeads® MyOne™ C1 (1 μm diameter, 10 mg/mL; ThermoFisher Scientific) were used as the particles. These beads consist of iron oxide cores surrounded by a polystyrene shell functionalized with streptavidin. The high-salt (HS) buffer used throughout the experiments consisted of PBS buffer supplemented with 0.5 M NaCl.

Methoxy polyethylene glycol-biotin (mPEG-biotin, molecular weight 1 kDa) was ordered at Nanocs (USA). For surface functionalization, poly(L-lysine)-grafted-poly(ethylene glycol) (PLL(20)-g[3.5]-PEG(2); SuSoS), referred to as PLL-g-PEG, and azide-functionalized PLL-g-PEG (PLL(15)-g[5]-PEG(2)-N3; Nanosoft Biotechnology LLC), referred to as PLL-g-PEG-N3, were used.

### Cartridge preparation

#### Surface functionalization

The COC cartridges were rinsed twice with Milli-Q water and subsequently cleaned by sonication in Milli-Q for 15 min. After cleaning, the cartridges were rinsed again twice and dried using a nitrogen stream. They were then exposed to UV ozone for 30 min using a UV Ozone Cleaner (Novascan). Immediately following UV ozone treatment, the flow chamber of each cartridge was sealed with an optically clear adhesive film (Thermo Fisher Scientific), and 25 µL of a polymer mixture was injected. This mixture consisted of 0.45 mg/mL PLL-g-PEG and 0.05 mg/mL PLL-g-PEG-N3, prepared in ultrapure Milli-Q water as described by Lin et al.^57^.

The cartridges were incubated with the polymer solution for 3 h at room temperature (RT) in a humidity chamber. Following incubation, the solution was extracted to remove unbound polymer. 25 µL of a DNA mixture was then added, containing 0.5 nM DBCO-dsDNA-biotin tether molecules (221 bp dsDNA) and 3 μM DBCO-ssDNA (DBCO-docking ssDNA hybridized to SSB), diluted in HS buffer. The DBCO-ssDNA was prepared by pre-hybridizing DBCO-docking ssDNA to the SSB at a 1:4 molar ratio (DBCO-docking ssDNA:SSB). This mixture was incubated for at least 2.5 h at RT on a rotary mixer. The inlet and outlet of the flow chamber were sealed after the addition of the DNA mixture, and the cartridges were stored in a humidity camber for a minimum of one week and up to four months.

#### Particle functionalization

4 µL of Streptavidin-coated Dynabeads MyOne C1 were incubated with 2.5 μL of PSB at concentrations of 1, 2.5, 5, or 10 µM, along with 10 μL PBS buffer, for 45 min at RT on a rotary mixer. To achieve a final ssDNA concentration of 10 µM on the particles, biotin-polyT was added together with 10 μL PBS buffer in volumes of 2.5 μL at concentrations of 9, 7.5, and 5 µM to the beads previously incubated with 1, 2.5, and 5 µM PSB, respectively. Following a 30-min incubation at RT on the rotary mixer, the particles were washed twice with 400 μL PBST (PBS buffer containing 0.05% Tween-20), resuspended in 300 μL HS buffer, and briefly sonicated for 10 s in a sonication bath (Branson 2800).

#### Cartridge activation

A prepared cartridge was rinsed with 200 μL HS buffer, after which 40 μL of freshly prepared particles were manually added using a pipette. The cartridge was then placed into a custom-built automated setup, and the particles were incubated for 10 min to allow binding to the biotin moiety on the substrate-side dsDNA tether. From this point onward, all fluid handling steps were performed automatically using a syringe pump at a flow rate of 100 μL/min with a flushing volume of 200 μL. Following particle tethering, residual biotin-binding sites were blocked by adding 100 μM 1 kDa biotin-mPEG. The biotin-PEG solution was added twice, with incubation times of 5 and 10 min, respectively. The cartridge was subsequently washed with HS buffer, and the bound fraction was continuously monitored to assess the activation process.

After the final HS buffer wash, the cartridge was ready for ssDNA sample (target 22 nt) measurements. All samples were diluted in HS buffer with 0.1% BSA. Each sample was flushed through the flow chamber at a flow rate of 100 μL/min for 2 min. In experiments involving multiple concentration series, each concentration (0, 16, 32, 63, 125, 250, 500 pM, 1 nM) was measured for 10 min. In dose-response measurements, each concentration (10, 50, 100, 250, 500, 750 pM, 1, 5, 10, 50, 100 nM) was measured once for 10 min. In the experiment involving a single concentration (750 pM), the measurement was repeated ten times, each lasting 15 min.

### Automated setup, data acquisition, and data processing

A Laboratory Programmable Syringe Pump (LSPone) and a 12-port rotary valve (AMF) were used to transport samples through the system. PTFE tubing (BL-PTFE-1608−20) and connectors (CIL-XP-245X) from Darwin Microfluidics were used to connect the pump and valve to the cartridge. A custom-made aluminum holder was used for holding the cartridge, with O-rings ensuring watertight connections between the cartridge inlet and outlet channels and the tubings. Between each sample, an air barrier was introduced to minimize cross-contamination. A Y-junction was placed before the cartridge entrance to divert air barriers to waste, preventing air from entering the cartridge.

Particle tracking within the flow chamber was performed using a custom-built optical setup consisting of a 10× DIN achromatic finite international standard objective (Edmund Optics), a 3 mm green LED (12 V), and a 3.2 MP camera (Flir BFS-U3-32S4M-C, software Spinnaker SDK version 2.4.0.114), providing a field of view of 0.71 × 0.53 mm with a pixel size of 0.345 µm. Autofocus was achieved using a miniature linear actuator (Zaber T-LA13A). The entire system, including pumps, valves, and microscope, was controlled via MATLAB.

Measurements were performed at a frame rate of 30 Hz after the sample was flushed through the flow chamber, ensuring data acquisition in the absence of flow. For experiments involving a single concentration, the sample was flushed in once. Real-time particle tracking was performed using software described by Bergkamp et al.^36^.

Post-processing to determine switching activity, bound fraction, and particle state lifetimes was done using a custom-made MATLAB script. The MATLAB code used to generate and analyze the simulated data in this study is available in the Code Ocean repository^58^: 10.24433/CO.7423379.v1. The equations for the analytical solution of the RNM model are provided in Supplementary Note 4.3. Pseudocode for the Monte Carlo simulation is provided in Supplementary Note 5.2.

### Statistics and reproducibility

The Monte Carlo simulations were performed 15 times for each condition to provide an estimate of the sampling distribution, from which imprecision and LOQ values were determined. The number of repeated simulation runs (*N* = 15) was determined based on a trade-off between simulation time and uncertainty on the measurement precision (determined to be ~19%, see Supplementary Note 6.2).

In the experiments, particles showing confined motion patterns in the first blank sample were excluded (minor motion amplitude ≤ 50 nm and symmetry ≤ 0.8), see Visser et al.^19,59^. The numbers of tracked particles in experiments were on the order of several hundreds, see for example Supplementary Fig. 19B. This gives signal variations (CVs) on the order of 1–10% (see Supplementary Fig. 19B), in agreement with simulations (see Fig. 4b, orange and purple lines). The variations are low enough for the scientific work of this paper.

The experiments were not randomized. The Investigators were not blinded to allocation during experiments and outcome assessment.

### Reporting summary

Further information on research design is available in the Nature Portfolio Reporting Summary linked to this article.

## Supplementary information

Supplementary Information

Reporting Summary

Transparent Peer Review file

## Source data

Source Data

## Data Availability

Datasets generated in this study are available in the Code Ocean repository^58^: 10.24433/CO.7423379.v1. Source data are provided with this paper.

## Code availability

The MATLAB code used to generate and analyze the simulated data in this study is available in the Code Ocean repository^58^: 10.24433/CO.7423379.v1.
