# Supplementary material for: Single-molecule readout of reversible nanoswitches enables continuous monitoring of low biomarker concentrations
Source: Nat Commun. 2026 Apr 18;17:5412. doi: 10.1038/s41467-026-71690-8 (PMC13280497; doi:10.1038/s41467-026-71690-8)
Supplement: Supplementary file 1 — Supplementary Information [file 41467_2026_71690_MOESM1_ESM.pdf]

Supplementary Information

Single-molecule readout of reversible nanoswitches enables continuous monitoring of low biomarker concentrations

Chris Vu[1,2], Selina A. J. Janssen[1,2], Arthur M. de Jong[2,3] & Menno W. J. Prins[1-4]

[1]Department of Biomedical Engineering, Eindhoven University of Technology, The Netherlands.
[2]Institute of Complex Molecular Systems (ICMS), Eindhoven University of Technology, The Netherlands.
[3]Department of Applied Physics and Science Education, Eindhoven University of Technology, The Netherlands.
[4]Helia Biomonitoring, The Netherlands.

Contents

Supplementary Notes 3
1. Readout methods in affinity-based biosensing 3
1.1. Reaction kinetics 3
1.2. Simulating a direct binding sensor with different readouts 3
2. Biosensing by Particle Motion 6
2.1. Sensing principles 6
2.2. Continuous monitoring of ssDNA using a sandwich BPM sensor 7
3. Model assumptions 9
3.1. Constant analyte concentration 9
3.2. Estimation of intra-nanoswitch sandwich formation rate k^{*}_{on} 10
4. Rate-based nanoswitch model – Analytical Approach 13
4.1. Model definition 13
4.2. Model for a nanoswitch with three binders 14
4.3. Generalized model for n states 14
4.4. Behavior of reversible nanoswitches 15
4.5. Response of reversible sandwich nanoswitches in limiting regimes 18
4.6. Comparison to experimental data 19
4.7. Cooperativity and anti-cooperativity 21
4.8. Sensor response with fixed and Poisson-distributed numbers of binders per particle 22
5. Rate-based nanoswitch model – Monte-Carlo implementation 23
5.1. Model description 23
5.2. Simulation steps 23
5.3. Simulation output 24
6. Sensor imprecision 27
6.1. Expression for the concentration imprecision 27
6.2. Uncertainty on the measurement imprecision 27
6.3. Time dependencies in two-step reactions 29
6.4. Analysis of signal imprecision (CV_S) in BPM 31
6.5. Determination of LOQs 34
7. Nanoswitch including non-specific interactions 36
7.1. Modelling non-specific binding in reversible nanoswitches 36
7.2. Measurement imprecision and LOQ 37
Supplementary References 41

Supplementary Notes

1. Readout methods in affinity-based biosensing

Figure 1 of the main text illustrates the concepts of ensemble-based sensing versus single-molecule sensing using a Monte Carlo simulation. This Supplementary Note explains the reaction equations that underlie the model, the setup of the Monte Carlo simulation, and the sensor readout parameters shown in Fig. 1.

1.1. Reaction kinetics

We describe a biosensor that uses direct binding to quantify analyte molecules in solution. The direct binding involves a reversible bimolecular reaction, where analyte A reversibly interacts with affinity binder B to form complex AB:

$$A + B \rightleftarrows AB. \tag{1.1}$$

The formation of complex AB is a second-order reaction. The rate of the reaction is determined by the molecular association rate constant k_{on}, with units $M^{-1}s^{-1}$, and the concentrations of the two reactants:

$$k_{A+B\to AB} = k_{on}[A][B]. \tag{1.2}$$

The dissociation reaction only involves the complex AB and the rate is proportional to the molecular dissociation rate constant k_{off} with unit s^{-1}. This is a first-order reaction with reaction rate:

$$k_{AB\to A+B} = k_{off}[AB]. \tag{1.3}$$

The affinity of a molecular interaction is characterized by the equilibrium dissociation constant K_D with unit M, and is defined as the ratio between the dissociation and association rate constants, which is equivalent to the ratio between the reactants and reaction product in equilibrium:

$$K_D = \frac{k_{off}}{k_{on}} = \frac{[A][B]}{[AB]}. \tag{1.4}$$

In case of analyte excess, the characteristic time required for the reaction to reach equilibrium τ_{eq} is governed by the rate of equilibration k_{eq} with unit s^{-1}:

$$\tau_{eq} = \frac{1}{k_{eq}} = \frac{1}{k_{on}[A] + k_{off}} \tag{1.5}$$

1.2. Simulating a direct binding sensor with different readouts

The simulation of Fig. 1 explores how two affinity binders with different K_D values respond to a step exposure to an analyte concentration of 1 pM. Two affinity binders are compared: a strong binder with a K_D of 10^{-10} M and a weaker binder with a K_D of 10^{-8} M. The association rates of both binders are 10^7 $M^{-1}s^{-1}$, so the differences in affinity are determined by differences in k_{off} only.

The sensor contains N_B binder molecules that can all individually interact with analyte molecules in solution. The analyte is assumed to be in excess, such that its concentration is assumed

constant throughout the measurement and depletion due to binding events is considered negligible. This assumption is discussed in more detail in Supplementary Note 3.

The association and dissociation of individual analyte-binder pairs is a stochastic process where each event occurs independently of the others. This can be modelled as a Poisson process, characterized by exponentially distributed waiting times between events. The mean binding waiting time $\tau_{binding}$, the mean time it takes for an affinity binder to bind an analyte molecule, is:

$$\tau_{binding} = \frac{1}{k_{on}[A]}. \quad (1.6)$$

The mean time that an analyte molecule stays bound to the affinity binder is the mean unbinding waiting time $\tau_{unbinding}$ and is the reciprocal of the dissociation rate constant:

$$\tau_{unbinding} = \frac{1}{k_{off}}. \quad (1.7)$$

In the Monte Carlo model, waiting times are continuously sampled from these two distributions to construct binding traces. In the ensemble-based readout, these can be summed and normalized by N_B to obtain the average occupation of affinity binders B by analyte molecules A. In the equilibrium condition, this gives:

$$f_B^A = \frac{N_{AB}}{N_B} = \frac{[AB]}{[B]_{total}} = \frac{[A]}{[A] + K_D}. \quad (1.8)$$

f_B^A follows from equation (1.4) after substituting [B], since the total concentration of binders is given by $[B]_{total}$ = [B] + [AB]. For the strong affinity binder, this gives $f_B^A = 10^{-12}/10^{-10} = 0.01$, while for the weak affinity binder, $f_B^A = 10^{-12}/10^{-8} = 10^{-4}$, in agreement with the simulation results.

In the single-molecule readout, all individual binding and unbinding events of all N_B binders are detected. The total number of events results in a frequency of events F_{events}:

$$F_{events} = \frac{1}{t}\sum_{i=1}^{N_B} N_{events}(i) \quad (1.9)$$

The upper limit of F_{events} is determined by the association rate process. Upon exposure of the sensor to analyte molecules, the average frequency of binding events per binder is $k_{on}[A] = 10^{-5}$ s^{-1}. Multiplying this frequency with N_B gives a total binding frequency of 10 bonds per second. The frequency is equal for both affinity binders, since their association rate constants are equal. Importantly, the time-to-equilibrium τ_{eq} is substantially smaller for the weak affinity binder than for the strong binder: 10 s versus 16 min, respectively. All model parameter values are listed in Supplementary Table 1.

Supplementary Table 1 | Input and output parameter values used in Fig. 1.

	Parameter	Value	Description
**Sensor characteristics**	$[A]$	10^{-12} M	Analyte concentration
	N_B	10^6	Number of affinity binders
	$t_{sampling}$	10 s	Sampling time to calculate binding rates
**High affinity binder**	k_{on}	10^7 $M^{-1}s^{-1}$	Association rate constant
	k_{off}	10^{-3} s^{-1}	Dissociation rate constant
	K_D	10^{-10} M	Equilibrium dissociation constant
	$\tau_{binding} = 1/k_{on}[A]$	10^5 s	Mean binding waiting time
	$\tau_{unbinding} = 1/k_{off}$	10^3 s	Mean unbinding waiting time
	$\tau_{eq} = 1/(k_{on}[A]+k_{off})$	10^3 s	Characteristic time-to-equilibrium
	$f_B^A = [A]/([A]+K_D)$	10^{-2}	Average molecular occupancy of B by A
	$F_{events} = k_{on}[A]N_B$	10 s^{-1}	Initial frequency of events
**Low affinity binder**	k_{on}	10^7 $M^{-1}s^{-1}$	Association rate constant
	k_{off}	10^{-1} s^{-1}	Dissociation rate constant
	K_D	10^{-8} M	Equilibrium dissociation constant
	$\tau_{binding} = 1/k_{on}[A]$	10^5 s	Mean binding waiting time
	$\tau_{unbinding} = 1/k_{off}$	10 s	Mean unbinding waiting time
	$\tau_{eq} = 1/(k_{on}[A]+k_{off})$	10 s	Characteristic time-to-equilibrium
	$f_B^A = [A]/([A]+K_D)$	10^{-4}	Average molecular occupancy of B by A
	$F_{events} = k_{on}[A]N_B$	10 s^{-1}	Initial frequency of events

2. Biosensing by Particle Motion

2.1. Sensing principles

In this paper, the modelling results were experimentally validated with data from Biosensing by Particle Motion (BPM) experiments[1,2]. BPM is a biosensing platform that relies on detecting state changes of mobile particles. The design of a tethered-BPM sensor is illustrated in Supplementary Figure 1A. Particles are molecularly tethered to the surface via a flexible double-stranded DNA tether, allowing the particles to freely move within the confinements of the tether. In the sandwich variant of the BPM sensor, both particle and surface are functionalized with affinity binders that transiently bind to analyte molecules in solution. The binding of an analyte molecule to both affinity binders leads to the formation of a sandwich bond that decreases the mobility of the particle, i.e., the particle transitions from the unbound state to a bound state. The changes in the particle motion are recorded using widefield video microscopy. Since the interactions between binders and analyte molecules are short-lived, the particles switch continuously between the unbound state and the bound state in the presence of analyte. At low analyte concentrations, very few sandwich bonds can be formed and the particle will mostly be in the unbound state (see Supplementary Figure 1B).

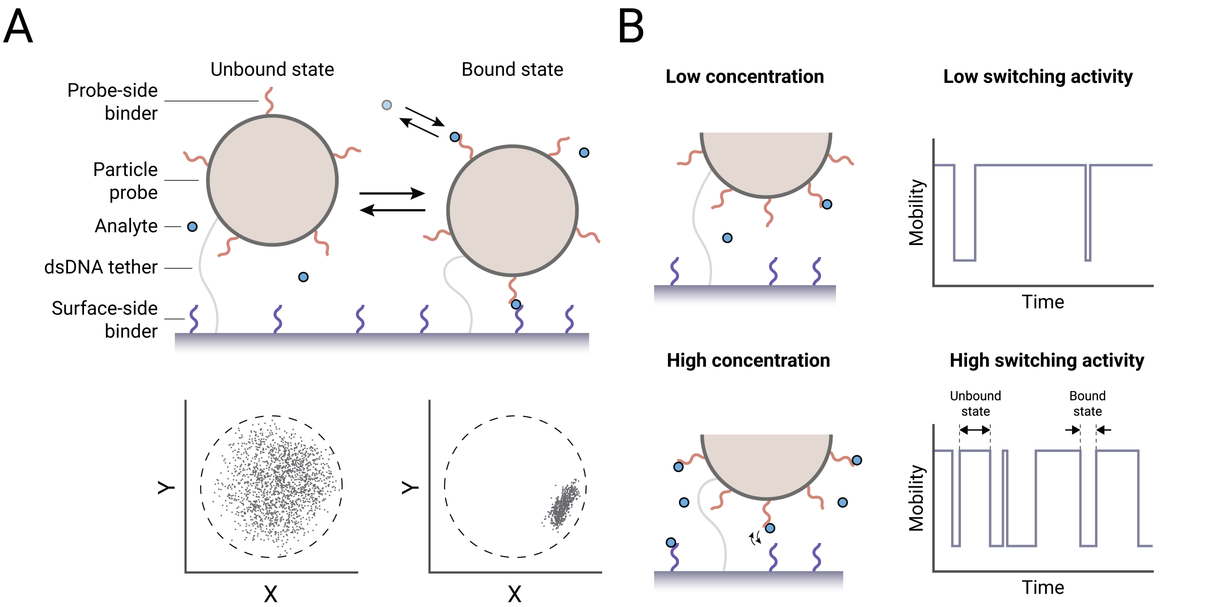

Supplementary Figure 1 | Measurement principle of Biosensing by Particle Motion (BPM) for sandwich-based detection of molecules. (A) Top: Micrometer-sized particles are tethered to the surface via a dsDNA tether. The particle and the surface are functionalized with affinity binders that can both reversibly bind to the analyte present in solution. The formation of a sandwich bond between particle and surface limits the Brownian motion of the particle. Bottom: examples of motion patterns of a particle in the unbound state and bound state. These are time-aggregated projections of the center of the particle in the xy-plane. (B) Sketch of signals in the BPM sensor. The transitions between states are digital signals obtained by tracking the mobility of the particle over time. Low analyte concentrations lead to infrequent switching events, high analyte concentrations lead to many switching events.

Supplementary Figure 2 shows how single-molecule response parameters can be extracted from a BPM experiment. Supplementary Figure 2A shows a section of a field-of-view in a BPM experiment. Hundreds to thousands of particles are localized and tracked simultaneously using previously described particle-tracking software[3]. The x- and y-positions are recorded over time for each identified particle. An example for a single particle is shown in Supplementary Figure 2B. Each change in the motion characteristics over time is recognized as an (un)binding event (red

lines). The switching activity of a single particle corresponds to the average number of (un)binding events per unit time. The period between events defines a binding state, which is classified as an unbound or bound state by comparing the calculated diffusion coefficient values with a threshold value. The bound fraction of a particle is determined by taking the ratio between the total amount of time a particle spends in the bound state and the total measurement time.

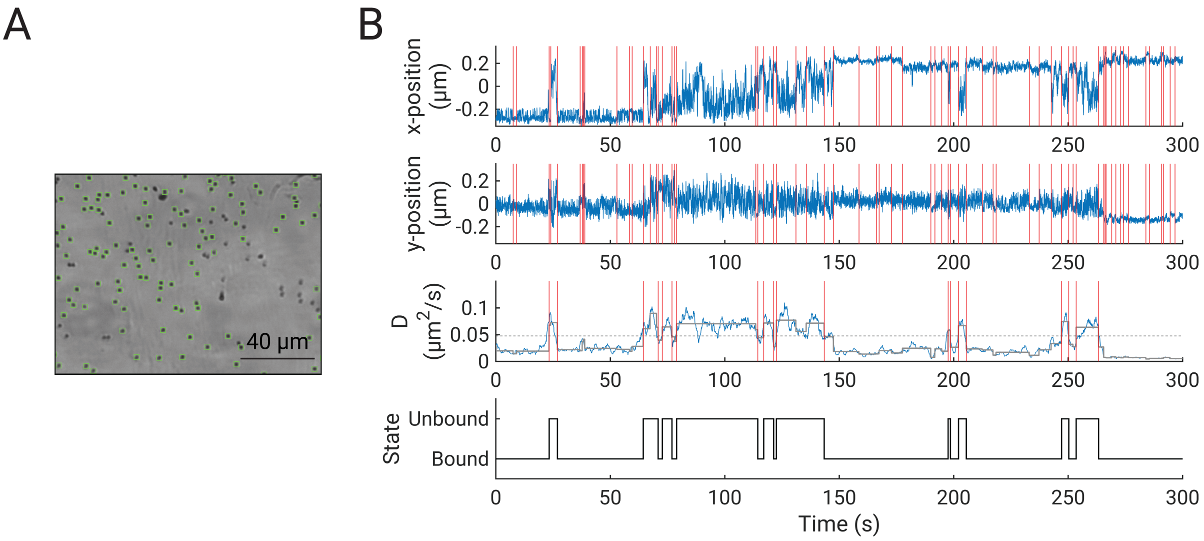

Supplementary Figure 2 | BPM signal processing. (A) Particle identification. Green squares indicate particles that are identified by the particle tracking software, previously reported by Bergkamp at al.[3] (B) Particle tracking and diffusivity-based state classification. The top two panels show the x- and the y-position of a particle over time. The xy-position data is used to determine change points (red lines), that correspond to changes in the motion trajectory of the particle induced by (un)binding events. The total number of change points normalized by the measurement duration is the switching activity of a particle. The xy-positional data is also used to calculate the diffusion coefficient of the particle over time, shown in the third panel (blue line). Particle states are defined by the previously determined change points, and the diffusion coefficient per state corresponds to the median of the distribution of diffusion coefficient values (grey line). The dashed line corresponds to the diffusion coefficient threshold for the classification of unbound (median higher than the threshold) and bound states (median lower than the threshold). Change points between two similar states have been removed; the remainder of change points is used for the calculation of the state-classified activity, used in Supplementary Note 4.6. The fully classified particle state trace is shown in the bottom panel.

2.2. Continuous monitoring of ssDNA using a sandwich BPM sensor

The model system used in this paper is a BPM sandwich sensor designed for the continuous monitoring of a single-stranded oligonucleotide (ssDNA) molecule (22 nt). The sensor used probe-side and surface-side ssDNA binders that have a 9 nt and an 8 nt base pair overlap with the analyte molecule, respectively. Further details can be found in the Materials & Methods section, Supplementary Note 8.

Supplementary Figure 3 shows an experiment where six concentration series of ssDNA were applied to the BPM sensor. The samples were consecutively flowed into the measurement chamber in an increasing fashion from 15 pM to 1 nM, after which the sensor reverted back to low signal values after measuring three blank samples. Over a period of 17 hours, a slight increase of signal is observed, which we attribute to non-specific interactions between particle and sensing surface.

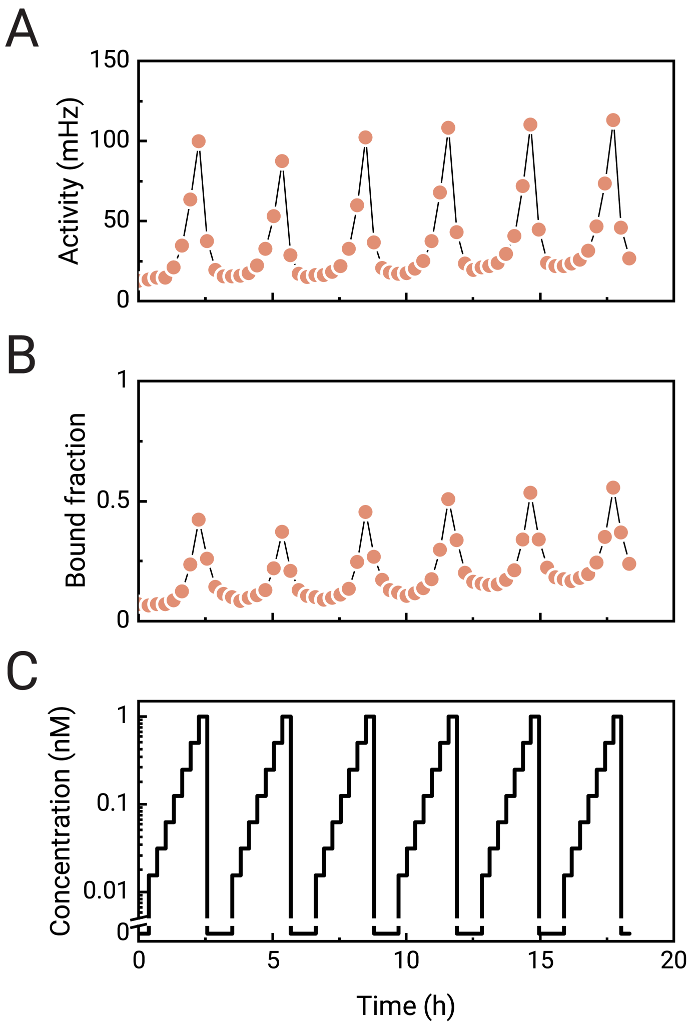

Supplementary Figure 3 | Continuous monitoring of ssDNA using a sandwich BPM sensor. (A) Activity signal over time. (B) Bound fraction signal over time. (C) Applied concentration over time. Source data are provided as a Source Data file.

3. Model assumptions

The rate-based nanoswitch model (RNM) described in the body text applies to an analyte-excess condition where the analyte concentration is constant. Furthermore, transport effects were neglected. Finally, the simulations were performed with an assumed value for the intra-nanoswitch sandwich bond formation rate. In this Supplementary Note, these assumptions are discussed and sensor designs are proposed that satisfy the assumptions.

3.1. Constant analyte concentration

In case of analyte excess, the analyte arrival events can be modelled as Poisson point processes with $\tau_{unbound}$ = 1/(k_{on}[A]) and the equilibrium time-averaged fractional occupancy of individual binders as Langmuir adsorption processes with f_B^A = [A]/([A]+K_D).

To investigate if the constant-analyte assumption is valid, the number of analyte molecules captured by the sensing surface needs to be compared with the number of analyte molecules present in the sensing volume. The sensing volume refers to the total volume of analyte-containing medium from which analyte molecules have a significant probability to reach and interact with the sensing surface on the time scale of the measurement.

Assume a number of nanoswitches on a sensing surface. The binding of analyte molecules to the sensing surface is given by a first-order ODE:

$$\frac{dN_{AB}}{dt} = k_{on}[A](N_B^0 - N_{AB}) - k_{off}N_{AB}, \tag{3.1}$$

where N_{AB} is the number of analyte-binder complexes, [A] the analyte concentration and N_B^0 the total number of binder molecules on the sensing surface. In the equilibrium condition (dN_{AB}/dt = 0), this gives:

$$N_{AB} = N_B^0 \frac{[A]}{[A] + K_D} \tag{3.2}$$

Assume a sensing volume with area A_s and layer height h_s. The number of analyte molecules present in this sensing volume equals:

$$N_A^{A_s.h_s} = [A] \cdot A_s \cdot h_s. \tag{3.3}$$

To obey the analyte-excess condition, $N_A^{A_s.h_s}$ should be much larger than N_{AB}. This gives:

$$N_A^{A_s.h_s} \gg N_{AB} \iff h_s \gg \frac{N_B^0}{A_S} \frac{1}{[A] + K_D} = \frac{\sigma_B}{[A] + K_D} \cong \frac{\sigma_B}{K_D}\ , \tag{3.4}$$

where $\sigma_B = N_B^0/A_s$ is the surface density of binder molecules and where we applied the condition [A] << K_D. This equation shows that the required height of the sensing volume is inversely proportional to K_D, i.e., h_s is small for biosensors based on low-affinity binders.

Using the parameter values listed in Supplementary Table 2 ($\sigma_B = 6 \cdot 10^5$ mm^{-2} = $6 \cdot 10^{11}$ m^{-2}; $K_D = 10^{-8}$ M = $6 \cdot 10^{18}$ m^{-3}), we find a sensing volume height h_s >> 0.1 μm. This means that the analyte-excess condition is met if analyte molecules from a distance larger than 0.1 μm are able to reach and interact with the sensing surface on the time scale of the measurement.

Assume a sensor with a sensing volume with limited height *H*. If analyte molecules are captured by the sensing surface, then the analyte concentration in the sensing volume decreases. A

relevant magnitude of H can be defined by the acceptable reduction of analyte volume concentration caused by analyte capture on the sensing surface. For example, if analyte volume concentration deviations of 1% are acceptable, then the sensing volume height should be $H > 100 \cdot h_s = 10$ µm.

Finally, we need to determine if molecular transport is sufficient in the sensing volume, i.e., if the analyte molecules within the sensing volume have a significant probability to reach and interact with the sensing surface on the timescale of the measurement. This is fulfilled if the time required for the analyte molecules in the bulk fluid to reach the sensing surface is smaller than the characteristic time-to-equilibrium of the surface reaction.

Assume a sensor with only diffusive transport and no advective transport. The characteristic diffusion time is given by $\tau_D = H^2/D$, where D is the analyte diffusivity. With $H = 10$ µm and $D = 10^{-10}$ m^2/s (see Supplementary Table 2), we find $\tau_D = 1$ s. The characteristic time-to-equilibrium of the surface reaction, in the condition $[A] << K_D$, is $\tau_{eq} = 1/k_{off} = 100$ s (equation (1.5)). Since $\tau_D << \tau_{eq}$, we conclude that diffusive analyte transport is fast with respect to the reaction time, thus the time-to-equilibrium of the sensor is determined by the surface reaction. If a sensor is designed with larger H, e.g., for reducing concentration deviations below 1%, then the influence of diffusive transport on the time-to-equilibrium of the sensor will increase.

To summarize, a sensor with the parameter values listed in Supplementary Table 2 and a sensing volume height of 10 µm operates in the analyte-excess regime with deviations of the analyte volume concentration of 1% or less, and has a time-to-equilibrium that is determined by the surface reaction and not by diffusive transport.

Supplementary Table 2 | Sensor design with sandwich nanoswitches, used to estimate the minimum sensing-volume height for obeying the analyte-excess condition.

Parameter	Value	Description
A_s	1 mm × 1 mm	Sensor surface area
$N_{nanoswitches}$	10.000	Number of nanoswitches
$N_{binders}$	30	Number of binders on each side of a nanoswitch
$\sigma_B = 2\, N_{binders} \cdot N_{nanoswitches} / A_s$	$6 \cdot 10^5$ mm^{-2}	Binder surface density (probe-side plus surface-side)
K_D	10^{-8} M	Equilibrium dissociation constant of the probe-side binder
$D = k_B T/6\pi\eta R_h$	10^{-10} m^2/s*	Diffusion coefficient

*Estimation based on a protein with a hydrodynamic radius R_h of ~2–3 nm (e.g., interleukin-6, ~21 kDa) in an aqueous solution at room temperature[4].

3.2. Estimation of intra-nanoswitch sandwich formation rate k^*_{on}

The values for the kinetic constants used in this study are summarized in Table 1 of the main text. While values for molecular rate constants (k_{on} and k_{off}) are well established, we are not aware of studies on values for intra-nanoswitch sandwich formation rates k^*_{on} . Parameter k^*_{on} is expected to depend on the affinities of the binder molecules and their encounter rates. The encounter rate depends on the distance and mutual accessibilities of the two binders, with one binder provided with an analyte molecule. For a particle-based nanoswitch, the distance and accessibility will depend on the tether length, the tether flexibility, the positions of the binder molecules on particle and surface, the particle size, and the particle surface roughness.

In this Supplementary Note, we use data from BPM experiments to estimate a value for k^{*}_{on}. BPM experiments provide distributions of particle unbound-state and bound-state lifetimes, which reflect the effective association rate and dissociation rates of the particle-based nanoswitches. By analyzing the unbound-state lifetime distributions, we attempt to give an estimation of k^{*}_{on} in a particle-based nanoswitch.

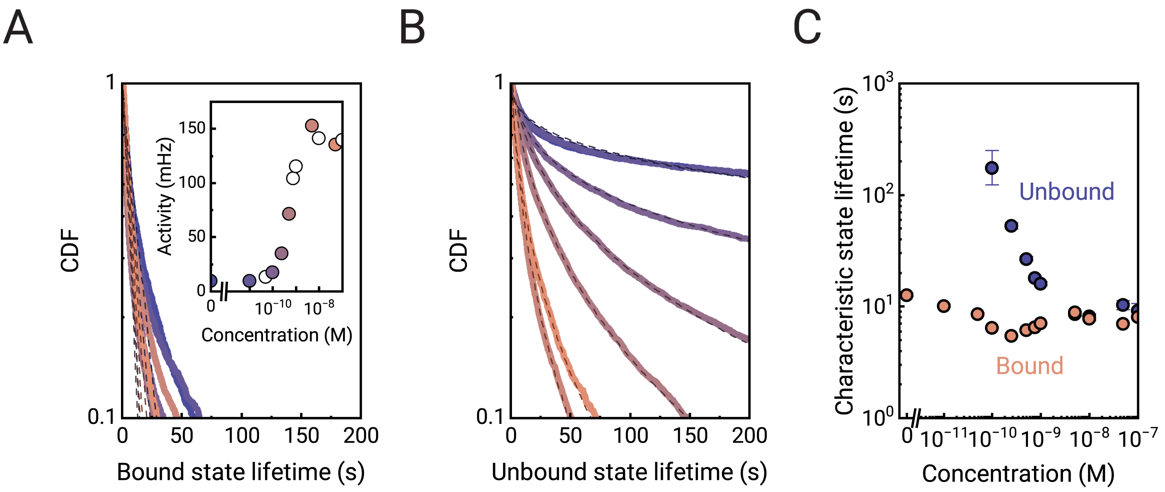

Supplementary Figure 4 | Characteristic state lifetimes in a BPM experiment. (A) Bound-state lifetime cumulative distribution functions (CDFs) measured in a continuous BPM sensor for sandwich-based ssDNA detection. Particles were incubated with 10 µM probe-side binders (see Materials and Methods Supplementary Note). The CDFs were fitted with function CDF = $e^{-t/\tau}$, with t the bound-state lifetime and τ the characteristic bound-state lifetime. The colors indicate the ssDNA concentration in solution, as shown in the inset. The inset shows the measured activity values as a function of the ssDNA concentration that was injected into the flow cell. (B) Unbound-state lifetime CDFs measured on the same sensor as in panel A. The CDFs were fitted with a multiexponential function as described by Lubken et al.[5]. The colors indicate the ssDNA concentration in solution, as shown in the inset in panel A. (C) Fitted characteristic bound-state lifetimes (orange) and unbound-state lifetimes (blue) as a function of ssDNA concentration. Error bars represent the 95% confidence interval of the fit. Characteristic unbound-state lifetime below 0.1 nM could not be reliably extracted due to strong right-censoring of long-lived states, and are therefore not reported. Source data are provided as a Source Data file.

Measurements were performed in a continuous BPM sensor for the sandwich-based detection of a single-stranded oligonucleotide (ssDNA) molecule with a length of 22 nt. The sensor employs probe-side and surface-side ssDNA binders that have a 9 nt and a 8 nt base pair overlap with the analyte molecule, respectively. Measurements consisted of adding different concentrations of the ssDNA analyte into the BPM flow cell and tracking particle mobilities for 10 minutes. The lifetimes were obtained by classifying each binding state using a custom-made MATLAB script, see Supplementary Fig. 2. Further experimental details can be found in the Materials and Methods (Supplementary Note 8).

Supplementary Figure 4A shows the cumulative distribution function (CDF) of the bound-state lifetime for selected concentrations. To limit the effect of long-lived non-specifically bound states in the analysis, only bound states were analyzed having well-defined start and end timepoints, i.e., left- or right-censored state lifetimes were not selected. The curves show single-exponential behaviors that do not depend on analyte concentration, with small deviations at the lowest and the highest ssDNA concentrations. At low concentrations, the deviations can be explained by the low numbers of bound states, causing false-positive events and non-specific interactions to appear in the data. At high concentrations, multivalently bound states appear, causing some longer bound-state lifetimes. This means that at high concentrations, the reversibility of the sensor can be affected, since longer relaxation times are required before multivalently-bound particles revert back to their unbound states.

The CDFs of the unbound-state lifetimes are shown in Supplementary Figure 4B. A significant fraction of particles was unbound during the entire measurement, therefore all unbound-state lifetimes were taken into account, including censored lifetimes. The CDFs appear multi-exponential, which can be attributed to heterogeneities in the association kinetics between particles (e.g., binder densities and accessibilities)[5–7]. The data shows that the unbound-state lifetimes decrease for higher concentrations, due to an increase in the binding frequency of the particles in the presence of analyte.

Estimates of the characteristic state lifetimes can be obtained by fitting the curves with exponential functions, see Supplementary Figure 4C. Bound-state lifetime CDFs were fitted with an single-exponential curve, while unbound-state lifetime CDFs were fitted with a multi-exponential curve that takes into account the heterogeneity in the characteristic unbound-state lifetime per particle[5]. Short characteristic bound-state lifetimes are observed for all concentrations (~5 to 10 s, below and above the observed EC_{50}, respectively). In the analyte concentration range between 0.1 and 1 nM, an increasing trend of the lifetimes as function of the concentration can be seen. In this range, high bound-state statistics are collected, e.g., $N_{[A] = 0.1\ nM}$ = 1,899 bound states, $N_{[A] = 0.25\ nM}$ = 4,294 bound states, and $N_{[A] = 0.5\ nM}$ = 7,687 bound states. The distributions are dominated by specific single-molecular binding events with a characteristic lifetime in the order of a few seconds.

Regarding the unbound-state lifetimes, the state statistics are low at low analyte concentrations, giving unreliable characteristic state lifetimes. Values for $\tau_{unbound}$ in the order of $10^1 - 10^2$ s are measured at concentrations below the EC_{50} of 0.25 nM. Since the fractional occupancies are low, such lifetimes are likely generated by nanoswitches that have captured at most one analyte.

If a nanoswitch has captured one analyte, then the observed particle unbound-state lifetime $\tau_{unbound}$ is maximal and equal to $1/(k^*_{on} \cdot N_{binders})$, with $N_{binders}$ the number of binders opposite to the analyte. This leads to the estimation: $k^*_{on} \sim 1/(N_{binders} \cdot \tau_{unbound}^{max})$. We do not yet have reliable numbers for $N_{binders}$ and $\tau_{unbound}^{max}$, but we estimate $\tau_{unbound}^{max}$ to be in the range 10^1–10^2 and $N_{binders}$ on the order of tens of molecules. Therefore, as a starting point, we use the value $k^*_{on} = 5 \cdot 10^{-3}\ s^{-1}$ in the simulations of this paper.

In a previous study[5], a sandwich-based ssDNA BPM sensor was studied with only a single surface-side binder incorporated in the dsDNA tether (N_{SSB} = 1). Smooth silica particles were used, instead of the less smooth polystyrene particles used in the experiments of the present paper. The PSBs were designed with a higher affinity to the target than the single SSB, so at low analyte concentrations the analyte is presumably bound to the particle. Therefore, $\tau_{unbound}^{max} \sim 1/(N_{SSB} \cdot k^*_{on}) = 1/k^*_{on}$. The observed values for $\tau_{unbound}^{max}$ were in the range $10^2 - 10^3$ s, so the estimated $k^*_{on} = 10^{-2} - 10^{-3}\ s^{-1}$. This range includes the k^*_{on} value used in the simulations of this paper.

4. Rate-based nanoswitch model – Analytical Approach

4.1. Model definition

The rate-based nanoswitch model (RNM) describes a nanoswitch as a system with two parts that each have a number of binder molecules: N_{PSB} on the probe side and N_{SSB} on the surface side. These molecules can interact with analyte molecules with concentration [A] in solution according to their kinetic rate parameters: $k_{off,PSB}$ and $k_{on,PSB}$ (with equilibrium dissociation constant $K_{D,PSB} = k_{off,PSB} / k_{on,PSB}$) for probe-side binders, and $k_{off,SSB}$ and $k_{on,SSB}$ (with equilibrium dissociation constant $K_{D,SSB} = k_{off,SSB} / k_{on,SSB}$) for surface-side binders.

When an analyte molecule is bound to a binder molecule, then subsequently a sandwich bond can be formed with a binder molecule on the opposite side, according to an intra-nanoswitch sandwich formation rate k^*_{on}, see Fig. 2b.

We are interested to calculate the probability that a probe is in a state with i sandwich bonds, expressed by the fraction parameter F_i, see equation (1) in the body text. The maximum number of sandwich bonds n in a given nanoswitch is determined by the minimum of N_{PSB} and N_{SSB}.

The RNM is based on expressions for the kinetic rate parameters for transitions between different states. The sandwich bond can be broken via the dissociation of either binder, so the dissociation rate of a single sandwich bond k_d is determined by the sum of dissociation rates:

$$k_d = k_{off,PSB} + k_{off,SSB}. \tag{4.1}$$

The nanoswitch forward rate $k_a^{(i)}$ describes the transition rate between F_i and F_{i+1}. The forward rate is determined by the number of bound analyte molecules that can interact with free binders on the opposite side and the intra-nanoswitch sandwich bond formation rate k^*_{on}. The number of bound analyte molecules follows from the fractional occupancy f of each binder. In the assumptions of analyte-excess and equilibrium, the fractional occupancy is described by the Langmuir isotherm:

$$f = \frac{[A]}{[A] + K_D}, \tag{4.2}$$

The number of available binders decreases with the number of sandwich bonds formed, so i bonds are subtracted from the number of binders. $k_a^{(i)}$ is therefore a state-dependent parameter that has lower values for higher-order states:

$$\begin{aligned} k_a^{(i)} &= k^*_{on} \cdot f_{PSB} \cdot (N_{PSB} - i) \cdot (1 - f_{SSB}) \cdot (N_{SSB} - i) \\ &+ k^*_{on} \cdot (1 - f_{PSB}) \cdot (N_{PSB} - i) \cdot f_{SSB} \cdot (N_{SSB} - i) \quad \Leftrightarrow \end{aligned} \tag{4.3}$$

$$k_a^{(i)} = k^*_{on} \cdot (N_{PSB} - i) \cdot (N_{SSB} - i) \cdot (f_{PSB} + f_{SSB} - 2 \cdot f_{PSB} \cdot f_{SSB}). \tag{4.4}$$

In this equation, all sandwich-forming interactions are assumed to be independent with equal sandwich bond formation rates k^*_{on}. The expression neglects potential cooperative and anti-cooperative effects. An example of a potential cooperative effect is that a first sandwich bond brings probe and surface in close proximity, which might cause an additional sandwich bond to form with higher probability. An example of a potential anti-cooperative effect is that the presence of a first sandwich bond hinders the translational and rotational freedom of the probe with respect to the surface, which might cause an additional sandwich bond to form with lower

probability. In the present model, both effects are neglected; further studies are needed to quantify the magnitudes of potential cooperative and anti-cooperative effects.

4.2. Model for a nanoswitch with three binders

For a nanoswitch with min(N_{PSB}, N_{SSB})=3, we can formulate the following reaction scheme:

$$F_0 \underset{k_d}{\overset{k_a^{(0)}}{\rightleftarrows}} F_1 \underset{2 \cdot k_d}{\overset{k_a^{(1)}}{\rightleftarrows}} F_2 \underset{3 \cdot k_d}{\overset{k_a^{(2)}}{\rightleftarrows}} F_3. \tag{4.5}$$

The dissociation pathways of sandwich bonds are assumed to be independent and to have equal rates, therefore the nanoswitch reverse rate of state F_i is equal to $i \cdot k_d$. This leads to the following set of ordinary differential equations (ODEs):

$$\frac{dF_0}{dt} = k_d \cdot F_1 - k_a^{(0)} \cdot F_0, \tag{4.6}$$

$$\frac{dF_1}{dt} = k_d \cdot (2F_2 - F_1) + k_a^{(0)} \cdot F_0 - k_a^{(1)} \cdot F_1, \tag{4.7}$$

$$\frac{dF_2}{dt} = k_d \cdot (3F_3 - 2F_2) + k_a^{(1)} \cdot F_1 - k_a^{(2)} \cdot F_2, \tag{4.8}$$

$$\frac{dF_3}{dt} = -3k_d F_3 + k_a^{(2)} \cdot F_2. \tag{4.9}$$

Since the nanoswitch distributes over the four states, the sum of probabilities is equal to unity:

$$F_0 + F_1 + F_2 + F_3 = 1. \tag{4.10}$$

In equilibrium, all time derivatives are zero and the set of equations can be analytically solved:

$$F_1 = \frac{k_a^{(0)}}{k_d} \cdot F_0, \tag{4.11}$$

$$F_2 = \frac{k_a^{(1)} k_a^{(0)}}{2k_d^2} \cdot F_0, \tag{4.12}$$

$$F_3 = \frac{k_a^{(2)} k_a^{(1)} k_a^{(0)}}{6k_d^3} \cdot F_0. \tag{4.13}$$

$$F_0 = \left(1 + \frac{k_a^{(0)}}{k_d} + \frac{k_a^{(1)} k_a^{(0)}}{2k_d^2} + \frac{k_a^{(2)} k_a^{(1)} k_a^{(0)}}{6k_d^3}\right)^{-1}. \tag{4.14}$$

4.3. Generalized model for n states

The model can be generalized to an arbitrary maximum number of states *n*, limited by the minimum number of binders of N_{PSB} and N_{SSB}:

$$F_0 \underset{k_d}{\overset{k_a^{(0)}}{\rightleftarrows}} F_1 \underset{2 \cdot k_d}{\overset{k_a^{(1)}}{\rightleftarrows}} F_2 \underset{3 \cdot k_d}{\overset{k_a^{(2)}}{\rightleftarrows}} \dots \underset{n \cdot k_d}{\overset{k_a^{(n-1)}}{\rightleftarrows}} F_n. \tag{4.15}$$

This gives the following generalized ODE for state *i*:

$$\frac{dF_i}{dt} = k_d \cdot ((i+1) \cdot F_{i+1} - i \cdot F_i) + k_a^{(i-1)} \cdot F_{i-1} - k_a^{(i)} \cdot F_i, \tag{4.16}$$

and:

$$\sum_{i=0}^{n} F_i = 1. \tag{4.17}$$

This provides the following expressions at equilibrium:

$$F_0 = \left(\sum_{i=0}^{n} \frac{1}{i! \cdot k_d^i} \cdot \prod_{j=0}^{i-1} k_a^{(j)} \right)^{-1} \tag{4.18}$$

and

$$F_i = F_0 \cdot \frac{1}{i! \cdot k_d^i} \cdot \prod_{j=0}^{i-1} k_a^{(j)}. \tag{4.19}$$

4.4. Behavior of reversible nanoswitches

Supplementary Figure 5 shows nanoswitch responses calculated with the equations of the previous Supplementary Note and the parameters in Table 1, using three values for k^*_{on}. The switching activity is plotted on the left y-axis (blue), which corresponds to transitions between the unbound and monovalent state. The temporal fractions of the states, in other words the average fraction of time a nanoswitch spends in a state (unbound, single bond, multiple bonds), are plotted on the right axis (orange lines). The multivalent bound state is defined as the sum of all higher-order fractions, i.e., $F_{2\to n} = \sum_{i=2}^{n} F_i$. The bound fraction reported in the main text corresponds to the total fraction across all bound states.

The graphs show how the measured switching events are influenced by the intra-nanoswitch kinetics. We first focus on the responses calculated with $k^*_{on} = 5 \cdot 10^{-4}$ s^{-1} (left panel). All output parameters show bell-shaped curves as a function of analyte concentration, with maximum or minimum values when the analyte concentration equals the geometric mean [A] = $\sqrt{K_{D,PSB} \cdot K_{D,SSB}}$. In this condition, the ratio of fractional occupancies f_{PSB}/f_{SSB} is approximately $\sqrt{K_{D,SSB}/K_{D,PSB}}$. Above the geometric mean, the unbound fraction increases and the bound fractions decrease due to the high-dose hook effect, caused by increasing occupations of both binder molecules by analyte molecules. These results show that the intra-nanoswitch sandwich formation rate is not high enough to drive the nanoswitch into states dominated by multivalent bonds.

Higher values for k^*_{on} increase the probability that sandwich bonds are formed in the nanoswitch. With $k^*_{on} = 5 \cdot 10^{-3}$ s^{-1}, the lowest unbound fraction value of the nanoswitch is about 0.1. Here, the nanoswitch is dominated by multivalent bonds rather than monovalent bonds. Consequently, a decrease in switching activity is observed, since the switching activity is the sum of the forward and reverse transitions between unbound and monovalent bound states. The maximal switching activity A_{max} occurs when the monovalent fraction reaches its highest value, which is the point where the switching activity changes from being association-limited to dissociation-limited. The second peak arises because probe- and surface-side binders get further saturated, so less

binders can participate in sandwich formation. This means that less multivalent bonds can be formed, increasing the probability that the nanoswitch is in a monovalent or unbound state, which leads to an increase in the switching activity. The nanoswitch with the highest k^*_{on} value (k^*_{on} = $5 \cdot 10^{-2}$ s^{-1}) shows responses at the lowest analyte concentrations. However, at the geometric mean the sensor is completely dominated by multivalent bonds and the switching activity approaches zero.

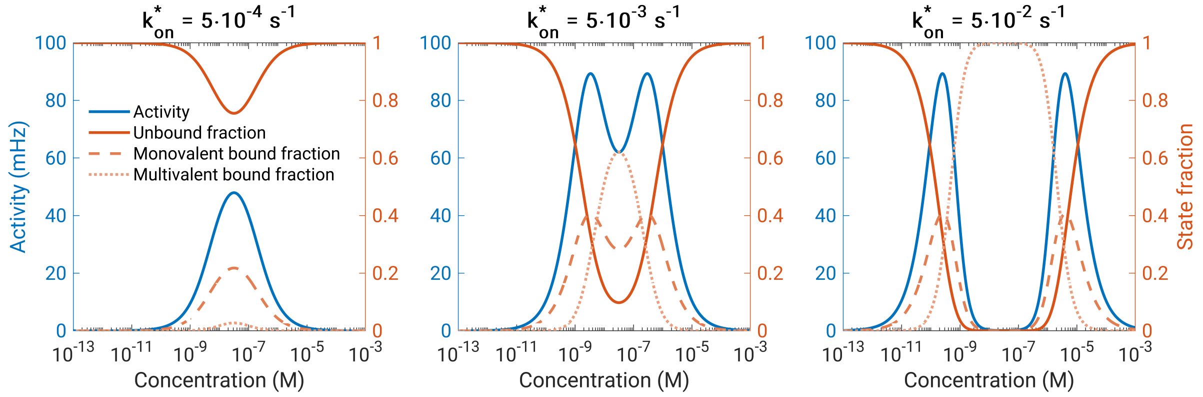

Supplementary Figure 5 | Behavior of reversible sandwich-based nanoswitches. Activity and state fractions as a function of analyte concentration, calculated for the parameters listed in Table 1, using three values for k^*_{on}, as shown at the top of the panels. The left axes indicate the activity (blue line), the right axes indicate the temporal fraction that nanoswitches spend in the unbound (no bonds, solid orange line), monovalently-bound (one bond, dashed orange line) and multivalently-bound state (two or more bonds, dotted orange line). Source data are provided as a Source Data file.

Supplementary Figure 6 shows the distribution of bonds formed, or the occupancies of states F_i, at the concentration when A_{max} is reached, for different numbers of PSB. The distribution of state occupancies changes with different numbers of binder molecules: increasing N_{PSB} redistributes the occupancies to higher-order states. The distribution of states behaves Poissonian, which becomes more clear at higher numbers of binder molecules. Moreover, the mean of the distribution tends to 1 for any set of nanoswitch parameters, meaning that – even at concentrations higher than the EC50 of the sensor – monovalent binding dominates the nanoswitch response, and the number of higher-order binding states remains low.

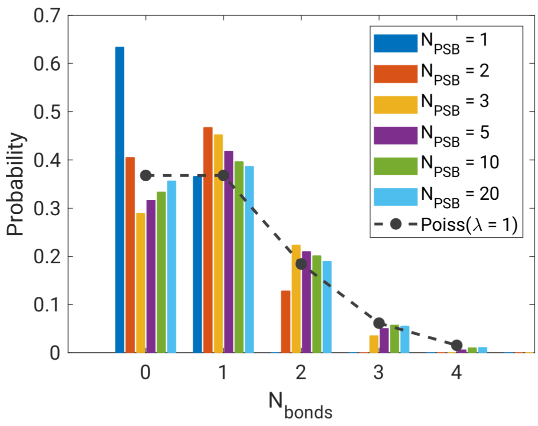

Supplementary Figure 6 | Distribution of nanoswitch states at A_{max} (N_{SSB} = 20). Responses were calculated with the kinetic constants listed in Table 1. The fractional states F_i correspond to the number of bonds N_{bonds} that were formed between probe and surface. The dashed line corresponds to the probability density function of a Poisson distribution with a rate λ = 1. Source data are provided as a Source Data file.

Figure 3 in the main text shows the activity signal for sandwich nanoswitches with varying numbers of binder molecules and dissociation rate constants. Supplementary Figure 7 shows the bound fraction signal for different molecular design parameters. Supplementary Figure 7A shows how the BF_{max} and EC_{50} parameters are defined. The left panel of Supplementary Figure 7D shows that for higher numbers of binders, the bound fraction for all curves eventually reaches 1, i.e., the rate of sandwich bond formation is high enough that nanoswitches are permanently bound. The right panel shows the bound fraction EC_{50} for different N_{PSB}, showing the same reciprocal scaling shown in Fig. 3c.

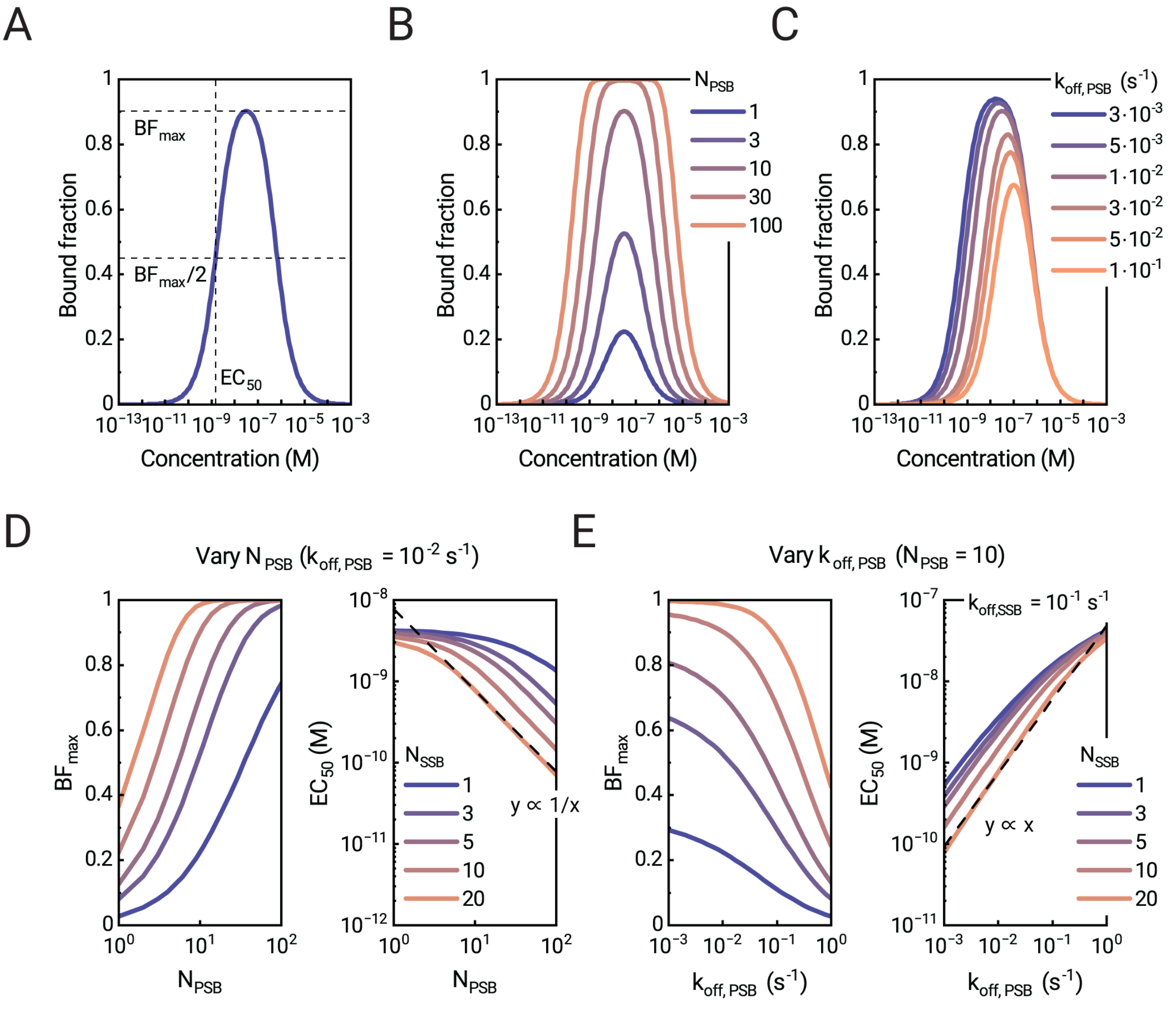

Supplementary Figure 7 | Thermodynamic response of reversible sandwich nanoswitches. (A) Deriving characteristic response parameters of the sensor response. BF_{max} indicates the maximum response of the sensor, while the EC_{50} corresponds to the concentration at which half of BF_{max} is reached. (B) Effect of the number of probe-side binders N_{PSB} on the sensor response. (C) Effect of $k_{off,PSB}$ on the sensor response. (D) Effect of N_{PSB} on the characteristic response parameters A_{max} (left) and EC_{50}. The EC_{50} is shown to scale with the reciprocal of N_{PSB} (black dashed line). (E) Effect of the probe-side dissociation rate $k_{off,PSB}$ on the sensor response. The EC_{50} is shown to linearly scale with $k_{off,PSB}$ (black dashed line). Source data are provided as a Source Data file.

The activity and bound fraction EC_{50} values are identical when no multivalency is possible, here shown for N_{SSB} = 1. However, when more than one bond can be formed in the nanoswitch, the calculated EC_{50} values are lower for the activity values, because the switching activity is proportional to the monovalent bound state F_1 and not to the total bound state. In monovalent

sandwiches F_0 and F_1 are directly coupled to each other because these are the only two possible states in the system, and thus the switching activity and bound fraction behave similarly. In a system where more than one bond is possible, the two states are decoupled since the total particle population is now distributed over additional higher-order states. As higher-order states increasingly dominate the state occupancies at high concentrations, the monovalent fraction peaks and decreases at a lower concentration than the geometric mean of the K_D values. Since the switching activity is directly proportional to F_1, it also peaks earlier under multivalent conditions.

4.5. Response of reversible sandwich nanoswitches in limiting regimes

This Supplementary Note discusses several limiting cases of the nanoswitches, to illustrate how the system behaves under extreme parameter values.

Supplementary Figure 8A shows the calculated response for very high numbers of PSBs. In the case of a monovalent sandwich setup (N_{SSB}, blue curve), the data demonstrates that the maximum switching activity A_{max} eventually saturates when N_{PSB} is higher than 100. This saturation marks the point at which the nanoswitch forward rate becomes sufficiently high that the system is dissociation-limited.

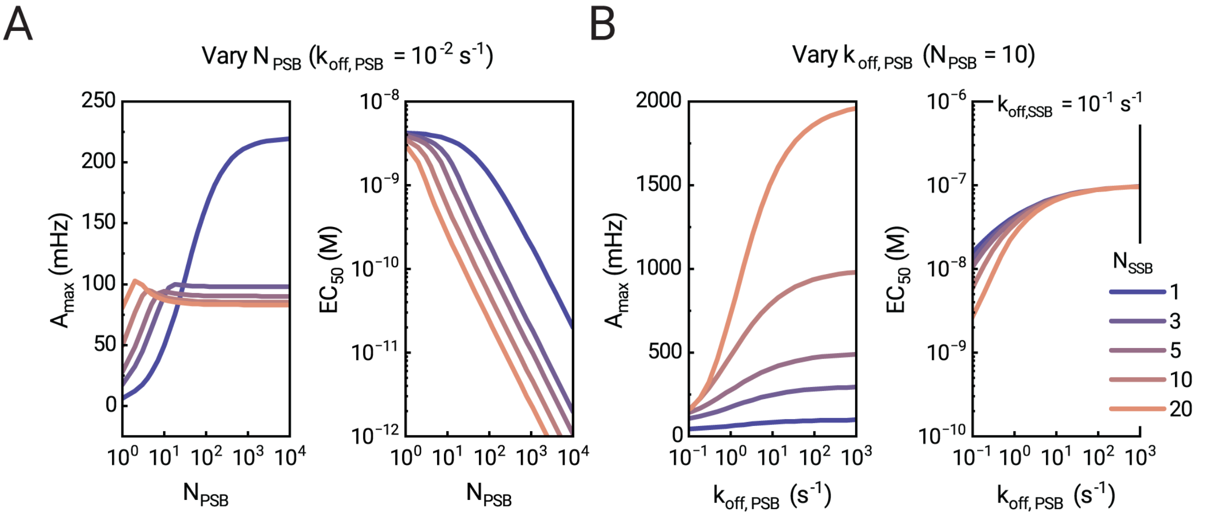

Supplementary Figure 8 | Response of reversible sandwich nanoswitches in limiting regimes. (A) Effect of N_{PSB} on the characteristic response parameters A_{max} (left) and EC_{50} (right). (B) Effect of the probe-side dissociation rate $k_{off,PSB}$ on the characteristic response parameters A_{max} (left) and EC_{50} (right). Source data are provided as a Source Data file.

Supplementary Figure 8B shows how extreme values of the dissociation rate constant of the PSB ($k_{off,PSB}$) affect the sensor response. In practical applications, very fast dissociation rates lead to nanoswitch bound states that would be too short-lived to be physically detectable, depending on the transduction mechanism used in the sensor. A_{max} is seen to saturate for high $k_{off,PSB}$. Here, the bound state lifetimes become infinitesimally short and each sandwich bond almost instantaneously dissociates, and the switching activity is limited by the nanoswitch bond formation process (in turn dictated by the intra-nanoswitch sandwich formation association rate and the analyte time-to-arrival). Under these conditions, A_{max} is purely determined by how long the analyte stays bound to the SSB.

The right panel shows how the EC_{50} scales with $k_{off,PSB}$. At high values, all SSB curves converge toward the same EC_{50}, dictated by $k_{off,SSB}$, because the sensor is in the association-limited regime. Combined with the fact that bound fractions are very low, the EC_{50} scales very weakly with the

number of binder molecules, similar to the association-limited regimes (low N_{PSB}) in Supplementary Figure 7D, right panel.

4.6. Comparison to experimental data

To validate the simulated behavior, we performed measurements with a BPM sensor designed for the continuous monitoring of a single-stranded oligonucleotide (ssDNA) molecule (22 nt). The sensor employs probe-side and surface-side ssDNA binders that have a 9 nt and an 8 nt base pair overlap with the analyte molecule, respectively. In other words, the probe-side binder has a higher affinity for the target than the surface-side binder. Further experimental details can be found in the Materials and Methods, see Supplementary Note 8.

In BPM, both particle probe and surface binder densities can be tuned by changing the incubation concentrations of the binder molecules during sensor fabrication. Here, similarly to the simulations shown in Fig. 3, the numbers of binder molecules were varied on the side with the higher-affinity binders, which in this case is the particle probe side.

The top panel of Supplementary Figure 9A shows measured dose-response curves (DRCs) for four different incubation concentrations of PSB. The DRCs have a sigmoidal shape with a background switching activity when no analyte is present, caused by non-specific binding events and false-positive events related to particle localizations and change-point detection. Increasing the binder densities (i.e., increasing $[PSB]_{incubation}$) shifts the curves to lower analyte concentrations. The maximum amplitude of the curves all lie around the same value (around 140 mHz), indicating that in all cases the sensor is operating in the dissociation-limited regime. The differences in maximum activity values are attributed to inter-sensor variabilities.

The activity signals shown in panel A were determined using a change-point detection algorithm that includes all changes in the motion trajectories[8], including potential higher-order binding transitions. Supplementary Figure 9B shows data from the same experiment, now plotted as the state-classified activity ($Activity_{SC}$). Here, particle binding states were classified based on their observed diffusivity in order to group all higher-order binding states into one category. All curves have bell shapes (complete or incomplete, see solid lines), that relate to the first peak at low analyte concentration discussed in Fig. 2d. As the analyte concentration increases from zero, the activity signal initially increases because of the formation of monovalent bonds between particle and surface (left side of the peak), and subsequently decreases when multivalent bonds dominate the response (right side of the peak). The width at half-maximum of the bell-shaped curves is between one and two concentration decades. These two observations are in agreement with the simulated activity DRCs of the RNM, see Fig. 3 and Supplementary Figure 5. The magnitude of $Activity_{SC}$ is about four times lower compared to the non-state-classified activity, which we attribute to short-lived bound states that are not taken into account. Supplementary Figure 9C shows the data from the same experiment, now plotted with the bound fraction as readout parameter.

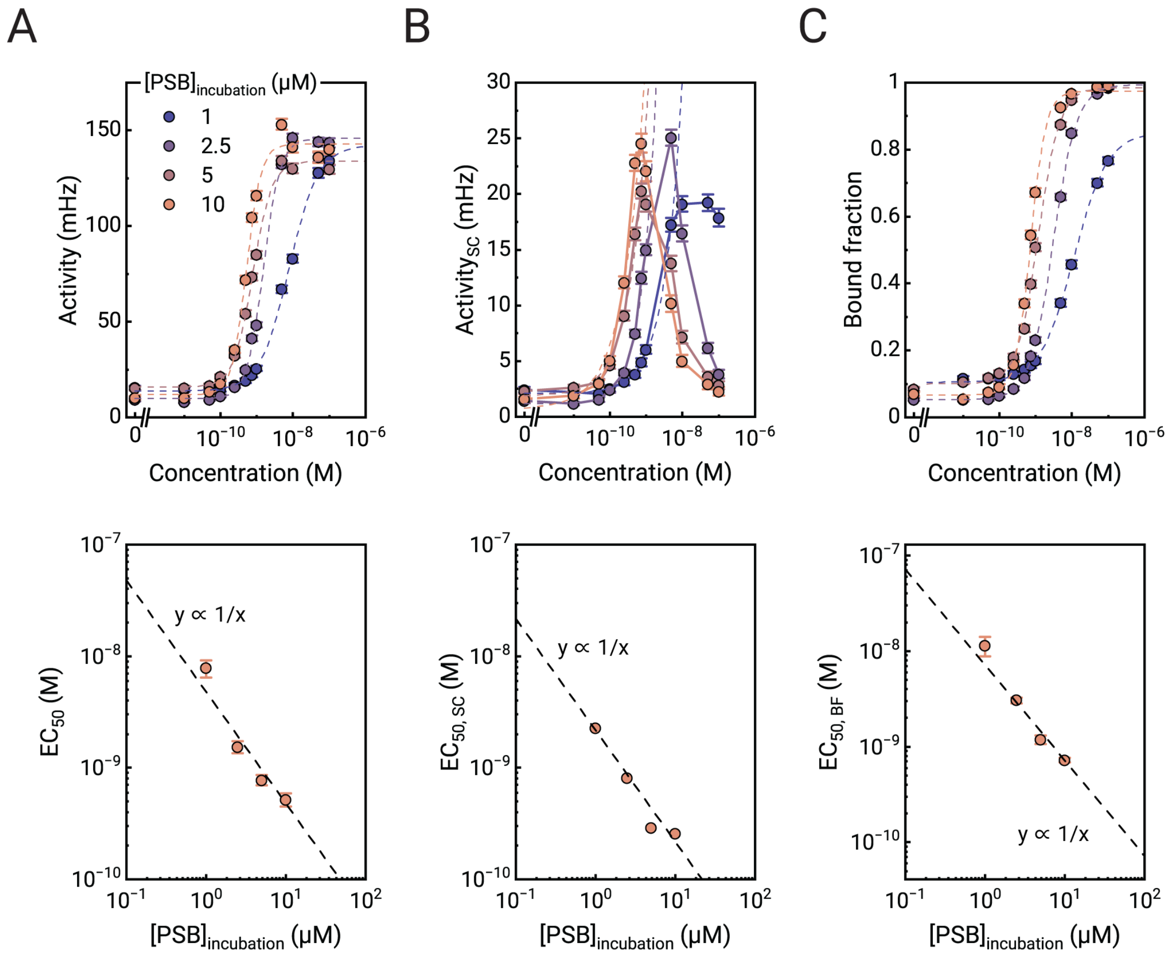

Supplementary Figure 9 | Experimental dose-response curves of sandwich-based ssDNA BPM nanoswitches. (A) Top: activity dose-response curves for different incubation concentrations of binder molecules on the particle probe ($[PSB]_{incubation}$). The incubation concentration on the surface-side was kept constant. The curves were fitted with a 4-parameter logistic fit to extract the EC_{50} ($y = a + (b - a) \cdot x^n/(x^n + EC_{50}{}^n)$). The error bars represent the estimated standard error of the mean of a single measurement, calculated by dividing the standard deviation over the responses of all particles with the square root of the total number of particles. Bottom: Extracted EC_{50} values for different binder incubation concentrations on the particle. The EC_{50} is shown to scale with the reciprocal of $[PSB]_{incubation}$ (black dashed line). The error bars represent the 95% confidence interval of the fit. (B) Top: State-classified activity dose-response curves for the same experiments shown in panel A. The solid lines are guide to the eye. The dashed lines are fits of the form $y = a + (x/c)^n$. Bottom: Extracted EC_{50} values for different $[PSB]_{incubation}$. The EC_{50} values were obtained by taking the half of the highest measured activity value and solving the equation for x. (C) Top: Bound fraction dose-response curves for the same experiments shown in panel A. The curves were fitted with a 4-parameter logistic fit to extract the EC_{50} ($y = a + (b - a) \cdot x^n/(x^n + EC_{50}{}^n)$). Bottom: Extracted EC_{50} values for different $[PSB]_{incubation}$. The error bars represent the 95% confidence interval of the fit. Source data are provided as a Source Data file.

Consistently, all three DRCs (activity, $activity_{SC}$, bound fraction) show shifts towards lower concentration for higher $[PSB]_{incubation}$. For all output parameters, the curves were fitted obtain their EC_{50} values (dashed lines). The bottom panels show how these values depend on incubation concentration. In all cases the EC_{50} is shown to scale reciprocally with $[PSB]_{incubation}$, confirming the behavior seen in Fig. 3 and Supplementary Figure 7. The lowest EC_{50} values are observed for the state-classified activity (panel B), followed by the activity and the bound fraction. This is in agreement with the decoupling between the switching activity and the bound fraction in the regime of multivalent binding, as discussed in Supplementary Note 4.4.

4.7. Cooperativity and anti-cooperativity

In the simulations, the kinetics of bond formation were modelled by assuming that all molecular interactions are independent. This is an approach in which potential cooperative and anti-cooperative effects are neglected. In a sandwich-based nanoswitch with on both sides multiple binders, cooperative as well as anti-cooperative effects can be present in principle, but the magnitudes of the effects and the net results are unknown and difficult to predict. An example of a potential cooperative effect is that the formation of a first sandwich bond brings probe and surface in closer proximity, which might cause an additional sandwich bond to form with a higher effective association rate than the first one. An example of a potential anti-cooperative effect is that the presence of a first sandwich bond hinders the translational and rotational freedom of the probe with respect to the surface, which can cause an additional sandwich bond to form with a lower effective association rate than the first bond if the respective binders are not directly aligned and oriented.

To determine whether cooperative effects play a role in the BPM experiments, we studied the slopes of dose-response curves. For example, the insets in Figs. 2c and 2d in the main text show simulation results using the model with independent interactions. In the low concentration regime, the slopes of the simulated dose-response curves are equal to one, in agreement with the assumption of independent interactions. Experimental data of a ssDNA sandwich-based BPM sensor are shown in Supplementary Fig. 9, and the dose-response data is reproduced in Supplementary Figure 10, plotted for three different readout parameters.

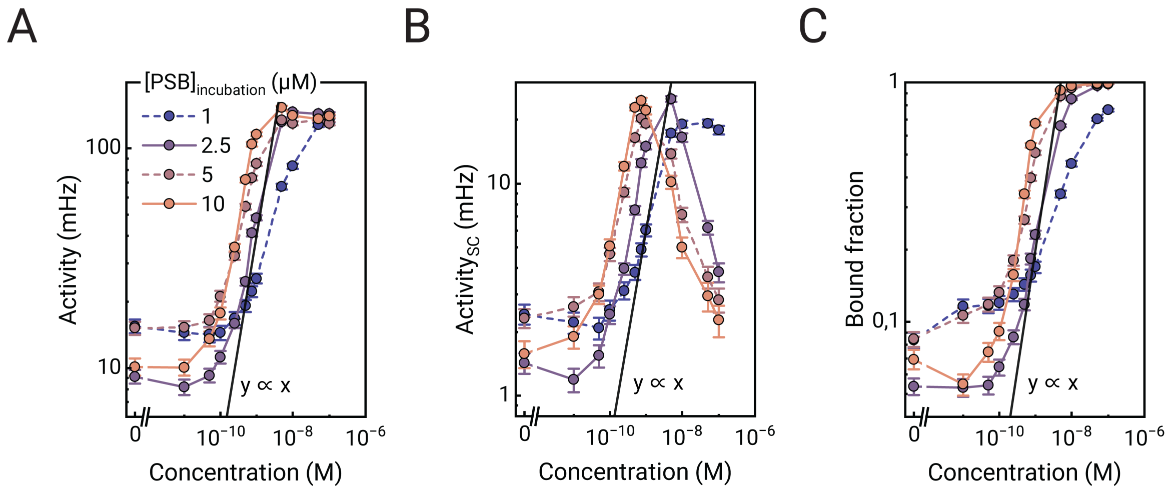

Supplementary Figure 10 | Experimental dose-response curves of sandwich-based ssDNA BPM nanoswitches, plotted on log-log scales. Lines between data points are guides for the eye. The black straight lines indicate a slope with linear dependence, i.e., a slope power equal to one (y ∝ x). (A) Activity dose-response curves. (B) State-classified activity (activity_{SC}) dose-response curves. (C) Bound fraction dose-response curves.

The data are plotted on log-log scales to highlight the slope behavior. In every panel, a black straight line indicates a slope with linear dependence, i.e., a slope power equal to one (y ∝ x). For all readout parameters, the graphs show that experiments with $[\text{PSB}]_{\text{incubation}}$ = 2.5, 5, and 10 μM give slopes close to one. The curves with the lowest density of binders on the particles ($[\text{PSB}]_{\text{incubation}}$ = 1 μM) have slopes that seem to be smaller than one for the activity and bound fraction readout parameters, but less clearly for the activity_{SC}. These data indicate that potentially a weak anti-cooperative effect is present for the lowest binder density on the particles, and no net (anti-)cooperative effects are seen for higher binder densities on the particles. However, further work is needed to study the slopes of the dose-response curves and clarify their origins.

For now, we conclude that the current modelling approach sufficiently represents the observed experimental behavior.

4.8. Sensor response with fixed and Poisson-distributed numbers of binders per particle

In BPM, the numbers of binder molecules are distributed due to the stochastic coupling of the binder molecules to particle and surface[6,7]. This Supplementary Note discusses the consequences of having distributions in the number of binder molecules.

Supplementary Figure 11 shows simulated response curves of two types of sensors: nanoswitches having all the same numbers of binders N_B (blue curve) and nanoswitches having Poisson-distributed numbers of binder molecules with mean value equal to N_B (orange curve, shaded area corresponds to the standard deviation over all nanoswitch responses).

Simulations were done for N_B ranging from 1 to 30 binders on the probe side and surface side. The sensors with a fixed number of binder molecules show higher maximum activity values than sensors with Poisson-distributed numbers of binders. This is especially clear for intermediate values for N_B, showing around 10% lower values for Poisson-distributed sensors. The difference is caused by a subpopulation of nanoswitches having zero binders on the probe and/or the surface side, causing these nanoswitches to have zero switching activity. For higher N_B, the responses of both sensors become more similar, as the likelihood decreases that a nanoswitch has zero binders. Moreover, the data show that the spread decreases for higher N_B (shaded area), since the coefficient of variation of N_B scales with $1/N_B^{1/2}$ according to Poisson statistics.

Therefore, in the regime of low binder densities, where the variation in the number of binders per nanoswitch is high, a sensor with homogeneously distributed binders per nanoswitch would be more sensitive than heterogeneously distributed binders per nanoswitch, as these on average would have higher switching activities. For higher binder densities, the difference in sensitivities between homogeneously and heterogeneously distributed binders per nanoswitch becomes smaller.

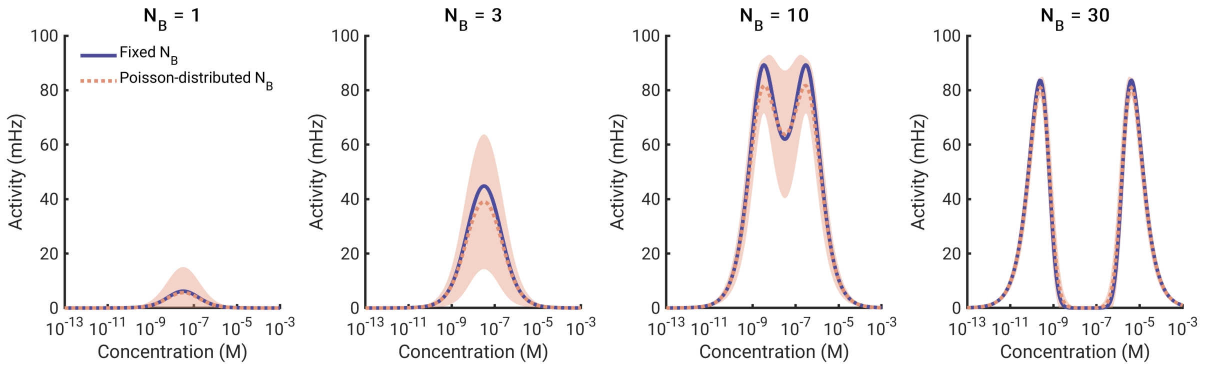

Supplementary Figure 11 | Sensor responses with fixed and Poisson-distributed numbers of binders per nanoswitch. The response for the sensor with Poisson-distributed numbers of binders N_B was calculated by averaging the responses of 10.000 nanoswitches with the analytical model, where each nanoswitch was assigned a different number of binders on either surface according to a Poisson distribution with mean N_B. N_{PSB} and N_{SSB} were both equal to N_B, as indicated above each panel. The orange shaded area corresponds to the spread of the Poisson-distributed dataset calculated using the mean ± the standard deviation of all 10.000 nanoswitch responses. Source data are provided as a Source Data file.

5. Rate-based nanoswitch model – Monte-Carlo implementation

This Supplementary Note describes the Monte Carlo model used to generate time traces of binding events in reversible nanoswitches, in order to calculate the switching activity and bound fractions of nanoswitches.

5.1. Model description

The Monte Carlo model considers a nanoswitch consisting of two parts, with N_{PSB} probe-side binders and N_{SSB} surface-side binders that interact with analyte molecules in solution (with concentration $[A]$) with their respective kinetic rate parameters k_{off} and k_{on}. Every molecular binding interaction is modelled as a Poisson point process with exponentially distributed waiting times. The waiting times related to analyte binding and unbinding of a single affinity binder are sampled from an exponential distribution with mean waiting times:

$$\tau_{binding} = \frac{1}{k_{on} \cdot [A]} \tag{5.1}$$

and

$$\tau_{unbinding} = \frac{1}{k_{off}}. \tag{5.2}$$

After an analyte is bound, the binder can then interact with all unbound binders on the other side according to a mean waiting time:

$$\tau_{sandwich} = \frac{1}{k_{on}^{*}}. \tag{5.3}$$

Following a sandwich formation event, a sandwich unbinding waiting time is also sampled for the opposite binder (from $\tau_{unbinding}$), such that each sandwich bond has two unbinding waiting times: one for the first bond and one for the second bond.

5.2. Simulation steps

A single simulation run consists of simulating a binding time trace of a single nanoswitch for a single analyte concentration. This involves the following steps:

1. Prior to starting the simulation, a number of parameters are defined, which are given in Supplementary Table 3.
2. A nanoswitch is generated with a number of binders given by N_{PSB} and N_{SSB}. N_{PSB} and N_{SSB} denote the number of binders per nanoswitch. These can be either a fixed number for all particles or a random number of binders generated according to a Poisson distribution.
3. A time matrix with size $(N_{PSB} + 2) \cdot (N_{SSB} + 2)$ is generated, that can contain all $N_{PSB} \cdot N_{SSB}$ sandwich formation waiting times, and analyte binding and analyte unbinding waiting times. In the initial step, only analyte binding waiting times are generated. The simulation starts at $t_{run} = 0$, which tracks the passed in-simulation time.
4. Per iteration, the shortest waiting time within the time matrix, related to a specific binding interaction, is selected and subtracted from all waiting times within the time matrix. This can lead to the following events for an individual binder:
    a. After binding of an analyte molecule to an affinity binder, sandwich formation waiting times are generated for all free affinity binders that are on the opposite

surface. Moreover, an analyte unbinding waiting time is generated for the newly formed binder-analyte complex.

b. After the formation of a sandwich bond, an analyte unbinding waiting time is generated for the second affinity binder that had just bound.

c. After unbinding of an analyte molecule, both analyte binding waiting times and sandwich formation waiting times (if analytes are present that happen to be bound on the opposite surface) are generated. If the affinity binder was part of a sandwich bond, new sandwich formation waiting times are generated for the remaining binder-analyte complex.

5. The subtracted waiting time is added to t_{meas}. For every iteration, the number of formed sandwich bonds is counted and recorded per t_{meas}.
6. If t_{meas} exceeds the total measurement time t_{total}, the simulation run ends and a new run is started with the same N_{PSB} and N_{SSB} and the next concentration in array A.
7. After finishing all simulation runs, the data is post-processed to obtain the switching activity and bound fraction per nanoswitch. The initial 1000 s in the simulation runs of Fig. 4a-c, and the initial 300 s in the simulation runs of Fig. 4d and e, were discarded to prevent artefacts from equilibration effects (see Supplementary Note 5.3).

Supplementary Table 3 | Input parameters for the Monte Carlo implementation of the RNM.

	Parameter	Description
**Sensing parameters**	$N_{nanoswitches}$	Number of simulated nanoswitches, defines the number of simulated time traces
	t_{total}	Total simulated measurement time
**Nanoswitch parameters**	N_{PSB}	Number of probe-side binders
	N_{SSB}	Number of surface-side binders
	$k_{on, PSB}$	Association rate constant of probe-side binders
	$k_{off, PSB}$	Dissociation rate constant of probe-side binders
	$k_{on, SSB}$	Association rate constant of surface-side binders
	$k_{off, SSB}$	Dissociation rate constant of surface-side binders
	k^{*}_{on}	Intra-nanoswitch sandwich bond formation rate

5.3. Simulation output

Supplementary Figure 12 shows example time traces generated by the model for different analyte concentrations, with similar simulation conditions as shown in Fig. 4. Two properties of the nanoswitch are highlighted: the number of formed sandwich bonds between probe and surface (top panels, red curves) and the observed state of the nanoswitch (bottom panels, grey curves) that follow from the sandwich bonds formed. The nanoswitch is in a bound state if at least one PSB-SSB sandwich bond is formed.

Supplementary Figure 13 shows an extensive example of intra-nanoswitch interactions. Highlighted are the observed state of the nanoswitch (green), the time in which a binder is bound to an analyte molecule (black line), the time in which a binder has formed a sandwich bond (grey thick bars) and whether this was initiated by an analyte molecule bound on a PSB (blue) or SSB (orange). The simulation conditions shown in Table 1 were used. Since the affinity is higher on the probe side than the surface side, many PSB-bound analyte molecules generate more switching events than SSB-bound analyte molecules.

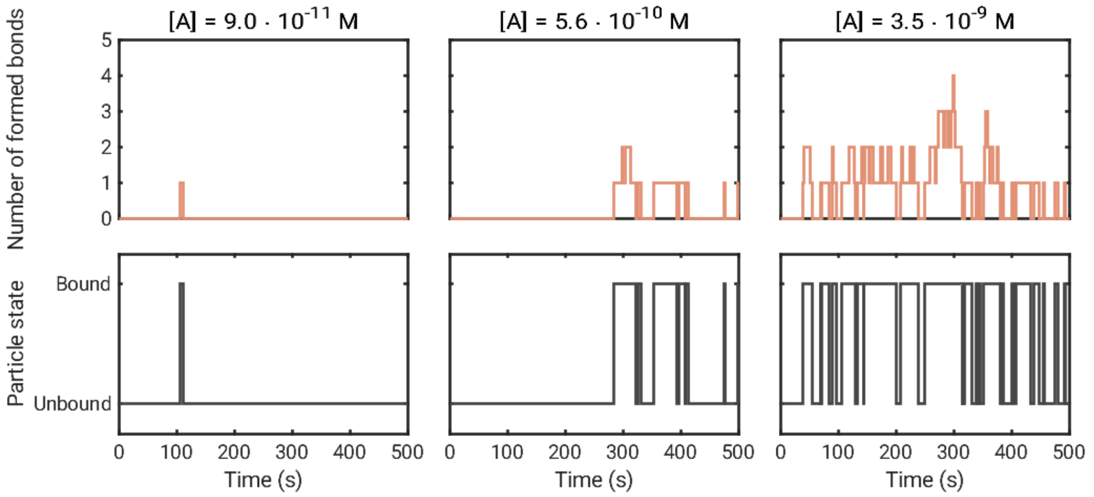

Supplementary Figure 12 | Examples of simulated time traces for different analyte concentrations. The same simulation conditions are used as shown in Fig. 4a (see also Table 1). Shown are the number of formed sandwich bonds in the top panels and the nanoswitch binding state in the bottom panels.

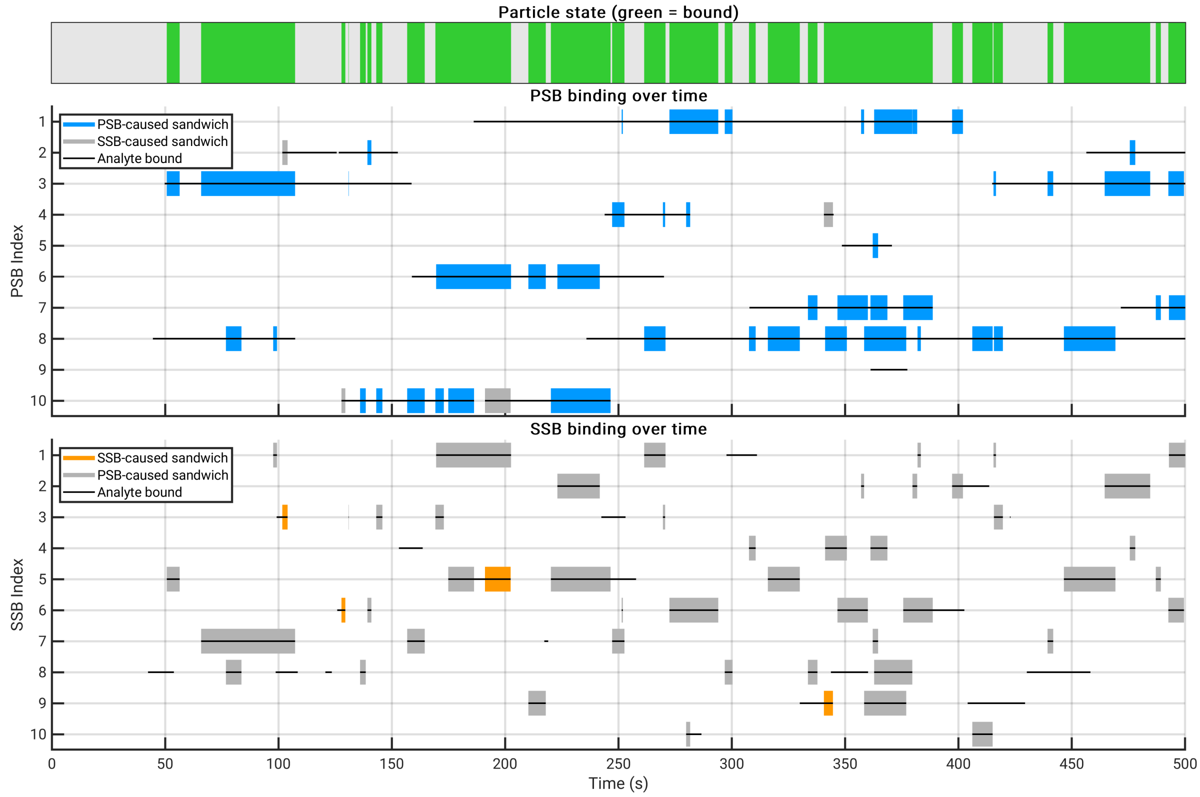

Supplementary Figure 13 | Example simulation run of a single particle nanoswitch. The nanoswitch bound state is indicated in green, analyte binding is indicated with the black lines. Sandwich bond formation is indicated with the thick bars, caused by PSB-bound analyte (blue) or SSB-bound analyte (orange). The bars are grey when the analyte was originally bound on the binder on the opposite side.

Supplementary Figure 14 shows the simulated response-time profiles using data from Fig. 4a. The activity values were calculated using a 30 s integration window moved every 10 s. The data is fitted with an exponential association curve of the form $y = A \cdot (1 - e^{-x/\tau})$, resulting in τ values of 123.3 ± 4.1 s ([A] = 0.09 nM), 84.9 ± 1.9 s ([A] = 0.56 nM, at the EC_{50}) and 25.1 ± 0.5 s ([A] = 3.5 nM, at A_{max}). The latter value is substantially faster due to signal saturation effects at high concentrations.

The results in Fig. 4 of the body text refer to equilibrium parameters. For extracting such equilibrium parameters, the initial non-equilibrium part of the simulation run (the initial 300 s in Supplementary Figure 14) were discarded to avoid any non-equilibrium related contributions in the output parameters.

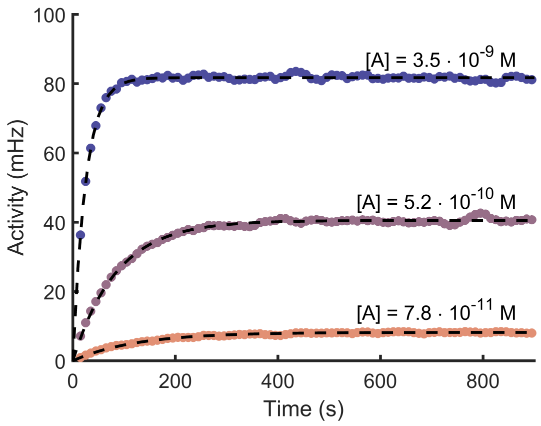

Supplementary Figure 14 | Simulated response-time profiles. The data was obtained from a single run of the same data set analyzed in Figure 4a-c. The data is fitted with a curve of the form $y = A \cdot (1 - e^{-x/\tau})$ (black dashed lines), resulting in τ values of 123.3 ± 4.1 s ([A] = 3.5 nM), 84.9 ± 1.9 s ([A] = 0.56 nM) and 25.1 ± 0.5 s ([A] = 0.09 nM) (reported as the parameter estimate ± the fitting errors based on a 95% confidence interval). Source data are provided as a Source Data file.

6. Sensor imprecision

6.1. Expression for the concentration imprecision

Assume a dose-response relationship with a 4-parameter logistic fit that expresses the signal S as function of the concentration C:

$$S(C) = S_{max} + \frac{S_{min} - S_{max}}{1 + \left(\frac{C}{EC_{50}}\right)^n}, \tag{6.1}$$

where S_{max} is the amplitude of the signal, S_{min} the background signal, EC_{50} the halfway concentration, and *n* the coefficient that describes the steepness of the curve.

The derivative of this function dS/dC is given by

$$\frac{dS}{dC} = \frac{(S_{max} - S_{min}) \cdot n \cdot C^{n-1}}{{EC_{50}}^n \cdot \left(1 + \left(\frac{C}{EC_{50}}\right)^n\right)^2}. \tag{6.2}$$

We are interested to find an expression for the coefficient of variation of the concentration determination CV_C, which is given by:

$$CV_C = \frac{\sigma_C}{C}. \tag{6.3}$$

The standard deviation of the concentration determination σ_C equals the standard deviation of the signal σ_S divided by the absolute value of the slope of the dose-response curve:

$$\sigma_C = \frac{\sigma_S}{\left|\frac{dS}{dC}\right|}. \tag{6.4}$$

Combining equations (6.2)-(6.4), we find:

$$CV_C = \frac{\sigma_S \cdot {EC_{50}}^n \cdot \left(1 + \left(\frac{C}{{EC_{50}}^n}\right)^n\right)^2}{(S_{max} - S_{min}) \cdot n \cdot C^n}. \tag{6.5}$$

At low concentrations (C << EC_{50}), equation (6.5) reduces to

$$CV_C = \frac{1}{n} \cdot \frac{\sigma_S}{S_{max} - S_{min}} \cdot \left(\frac{EC_{50}}{C}\right)^n. \tag{6.6}$$

The equation shows that the CV_C depends on the steepness *n* of the dose-response curve, on the signal variation σ_S with respect to the signal dynamic range $S_{max} - S_{min}$, and on the analyte concentration with respect to the EC_{50}.

6.2. Uncertainty on the measurement imprecision

This Supplementary Note describes how the uncertainty can be estimated on calculated CV values. We define the outcome value of a measurement *i* as x_i. In a single-molecule sensor, x_i is the average value of the signals produced by all probes in the sensor. When calculating the average value of a series of measurements, i.e., the average of a sampling distribution $\bar{x} = \frac{1}{N}\sum_i x_i$, an estimation is made of the true value of the mean μ of the sensor, i.e., $\bar{x} \approx \mu$. The same applies to the standard deviation of a sampling distribution (in other words, the standard error of the

mean), i.e., $s = \sqrt{\frac{1}{N-1}\sum_i (x_i - \bar{x})^2}$, is an estimation of the true standard deviation (or the population standard deviation) σ, so that $s \approx \sigma$. Throughout this work, we have purposefully referred to the estimates of the true values, $\bar{x}$ and s, as μ and σ, respectively, since readers are more familiar with those symbols.

Both $\bar{x}$ and s are estimates based on a finite number of samples and are therefore subject to uncertainties caused by limited statistics. The relative uncertainty of parameter s is approximately given by:

$$\frac{s_s}{s} \approx \frac{1}{\sqrt{2(N-1)}}, \tag{6.7}$$

where s_s represents the standard deviation calculated over the distribution of measured standard deviations[9]. Supplementary Figure 15 plots equation (6.7) for the number of samples, showing that the relative uncertainty s_s/s decreases as the number of samples, or runs, increases. In this work, to balance precision with the duration of the simulations, we opted to simulate at most 15 runs, resulting in an uncertainty of approximately 19% on the calculated CV_S values.

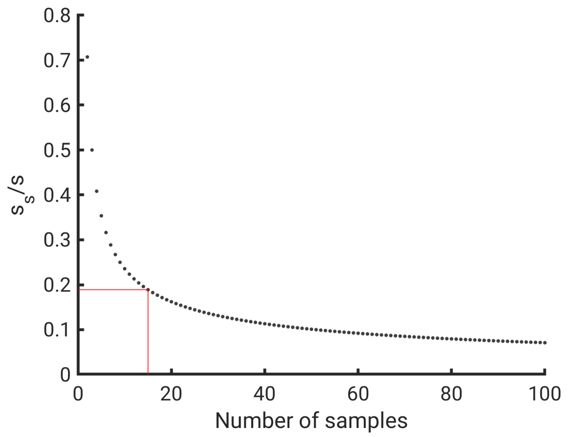

Supplementary Figure 15 | The relative uncertainty of parameter *s* as a function of the number of samples. The data points are calculated using equation (6.7). The red lines indicate the number of samples used in this work (15) to determine imprecisions and the corresponding uncertainty. Source data are provided as a Source Data file.

We investigated the distributions of s_s/s for different simulation conditions in the Monte Carlo model, see Supplementary Figure 16. The distributions were obtained for different N_{PSB} at a fixed N_{SSB} of 10 and an analyte concentration [A] of 61.6 pM. s_s is the sample standard deviation of a single sampling distribution containing 15 mean activity values obtained by sampling 100 particles per measurement run. This procedure was repeated 100 times to create a distribution of s_s. The relative width of this distribution corresponds to the uncertainty in estimating the CV. The distributions of s_s/s appear to be Gaussian, so the relative uncertainty could be estimated by fitting the data with a Gaussian curve. The obtained widths σ lie between 17 and 21%, confirming the expected statistical behavior of the simulation.

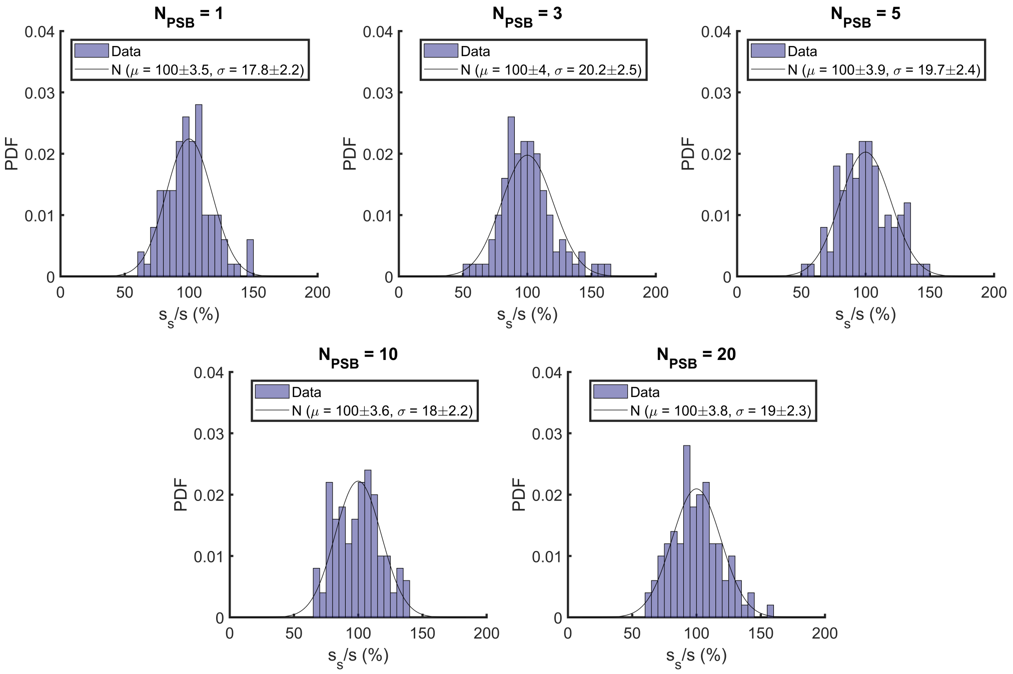

Supplementary Figure 16 | Distributions of the relative uncertainty of parameter *s* (s_s/s). The data set, with simulation conditions [A] = 61.6 pM and N_{SSB} = 10, are comprised of 15 runs containing 10.000 nanoswitches per run. s_s was obtained by taking the standard deviation of a distribution of 15 mean activity values, where each mean activity value was calculated by averaging the activity of 100 nanoswitches randomly sampled from the 10.000 nanoswitches within a run. This resampling procedure was repeated N = 100 times, yielding 100 values for s_s. The histograms are displayed as a probability density function and fitted with a Gaussian function. The fit parameters in the legend are reported as the parameter estimate ± the fitting errors based on a 95% confidence interval. Source data are provided as a Source Data file.

6.3. Time dependencies in two-step reactions

Figure 4c demonstrated that the Poisson behavior of sandwich nanoswitches changes at short timescales. In this Supplementary Note, we explore this in more detail and discuss how different temporal regimes influence the obtained measurement response.

Supplementary Figure 17 shows simulated lifetime distributions of the nanoswitch unbound and nanoswitch bound state, obtained from the same data shown in Fig. 4a-c. Analyzing these state lifetimes infers information regarding the kinetic regime of the sensor. For example, the right panel shows the nanoswitch bound-state lifetimes. In the regime of monovalent sandwich bond formation, the bound-state lifetimes should reflect the nanoswitch dissociation rate k_d (equation (4.1)). Indeed, fitting the cumulative distribution function (CDF) of the lowest simulated concentration ([A] = $9.0 \cdot 10^{-11}$ M, orange) with a single-exponential function yields a characteristic lifetime of approximately 9.4 s, corresponding to a dissociation rate of 1/9.4 ≈ 0.1 s^{-1}. The data shows that the distribution tends to longer lifetimes as the analyte concentration increases, which is due to the growing presence of multivalent sandwich bonds, increasing the nanoswitch bound-state lifetimes.

The simulated unbound-state lifetimes on the left show a trend that scales inversely with the concentration: lower concentrations lead to longer unbound-state lifetimes. Moreover, the single-exponential nature of the curve disappears for lower concentrations. This is very clear for the low concentration curve (orange) that shows two kinetic regimes; one characterized by short-lived states related to repeated switching when an analyte is bound to a nanoswitch for a longer

period of time, and another characterized by long-lived states that relate to nanoswitches without any bound analyte molecule. This means that the sandwich binding process does not behave like a simple one-step Poisson process at low concentrations, because sandwich formation events are strongly correlated and depend on the previous states of the system (i.e., the process is not memoryless). The consequence is that the distribution of events per nanoswitch is burst-like: within a same time window, nanoswitches show either many events or no events.

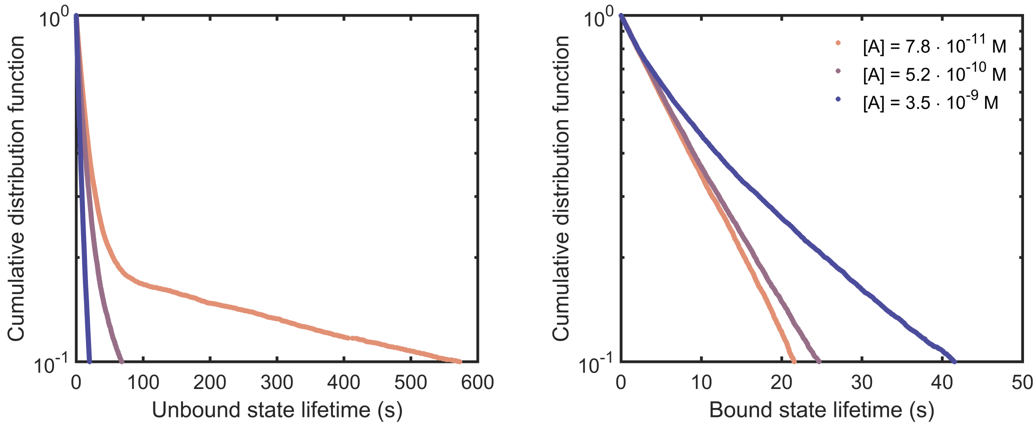

Supplementary Figure 17 | Analysis of simulated particle state lifetimes. The unbound state lifetimes are shown in the left panel, the bound state lifetimes are shown in the right panel. The lifetimes were extracted from the particle unbound and bound states in the data of Fig. 4a-c. Source data are provided as a Source Data file.

To illustrate the burst-like behavior, we set up a simplified Monte Carlo simulation based on the following reaction pathway:

$$B_1 + A \underset{k_d^{(1)}}{\overset{k_a^{(1)}}{\rightleftarrows}} B_1A + B_2 \underset{k_d^{(2)}}{\overset{k_a^{(2)}}{\rightleftarrows}} B_1AB_2 \tag{6.8}$$

and an irreversible dissociation step:

$$B_1AB_2 \overset{k_d^{(1)}}{\rightarrow} B_1 + A + B_2. \tag{6.9}$$

This reaction scheme resembles the sandwich reaction pathway where A is the analyte molecule in solution, B_1 the immobilized binder with a relatively high affinity, B_2 the secondary binder with a relatively low affinity, and k_a and k_d denote effective, concentration-independent on- and off-rates related to their respective binder molecules. An important model assumption is that the sandwich complex cannot reform when A and B_1 dissociate, because the interaction of A and B_2 is short-lived with respect to B_1A. Thus, once A binds to B_1, an irreversible dissociation time is sampled from an exponential distribution with rate $k_d^{(1)}$, and the dissociation of complex B_1AB_2 is a kinetic competition between the residual dissociation time from the original B_1A complex and the dissociation of the secondary binder B_2 with rate $k_d^{(2)}$.

Supplementary Figure 18 shows calculated CV_S values from simulations with three different values of $k_a^{(1)}$ (10^0, 10^{-2} and 10^{-4} s^{-1}). The switching activity was obtained by counting all transitions from and to complex B_1AB_2, the used parameter values are shown in Supplementary Table 4. The CV_S curves show the same profiles as seen in Fig. 4c. Specifically, when the analyte association rate $k_a^{(1)}$ is fast with respect to the characteristic timescale of switching ($\tau_{switch} = 1/k_a^{(2)} + 1/k_d^{(2)} = 20$

s), the curve follows the expected $1/\sqrt{t_{sampling}}$ scaling. When $k_a^{(1)}$ is slow compared to this timescale, the scaling breaks down for sampling windows shorter than the slowest dissociation time ($\tau_{eq} \approx 1/k_d^{(1)} = 100$ s). In this regime, too few transitions take place within the sampling window to accurately capture the underlying kinetics. When measurement times are sufficiently long, the Poisson-like scaling is restored. Now, enough switching events are accumulated to smoothen out temporal correlations and the kinetic rates are properly sampled, leading to a memoryless system.

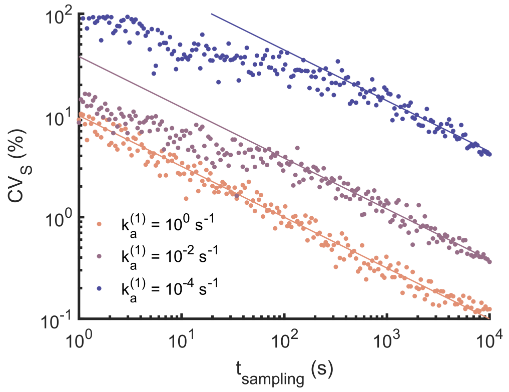

Supplementary Figure 18 | Time dependencies in two-step reaction pathways. The simplified reaction scheme in equations (6.8) and (6.9) is simulated for different values of $k_a^{(1)}$, which represents the effective association rate of an analyte molecule to the primary binder molecule. The lines are fits of the form $y = a/x^{1/2}$. Source data are provided as a Source Data file.

Supplementary Table 4 | Input parameters for the simplified Monte Carlo simulation shown in Supplementary Figure 18.

Parameter	Value	Description
$k_d^{(1)}$	10^{-2} s^{-1}	Effective dissociation rate between A and B_1
$k_a^{(2)}$	10^{-1} s^{-1}	Effective association rate between A and B_2
$k_d^{(2)}$	10^{-1} s^{-1}	Effective dissociation rate between A and B_2
$N_{particles}$	10^3	Number of particles
$t_{simulation}$	$1.1 \cdot 10^4$ s	Total simulation time
$t_{discard}$	10^3 s	Initial simulation time that is discarded from the simulation
N_{runs}	15	Number of trials

6.4. Analysis of signal imprecision (CV_S) in BPM

In Fig. 4, we analyzed scaling laws concerning the signal imprecision as a function of sensor acquisition parameters. In this Supplementary Note, we analyze the experimental signal precision in a BPM sensor to validate the scaling laws and to investigate to what extent the sensor is Poisson-limited.

A BPM experiment was performed using the ssDNA sandwich sensor where the sensor was supplied with a concentration near the EC_{50} of the system ([A] = 750 pM, same experimental conditions where $[PSB]_{incubation}$ = 10 μM in Supplementary Figure 9). The sample was repeatedly measured for 10 times, with 15 minutes per measurement. The data is shown in Supplementary Figure 19A (open circles), plotted as the raw, non-state classified activity (as discussed in Supplementary Note 4.6).

The raw experimental data in panel A shows a decreasing trend over the duration of the experiment, from ~105 mHz to ~80 mHz. This decrease is related to long-term sensor changes such as losses of binder molecules and non-specific interactions[7,10]. The signal imprecision of the sensor (CV_S) relates to the short-term fluctuations of the signal. To be able to quantity the CV_S, the long-term signal changes were removed using a linear fit, as indicated in panel A. The raw data (open symbols) was fitted to a line of the form $y = A_0 - k_{decay}t$, to produce corrected data of the form $A_{corr}(t) = A(t) + k_{decay}t$ (closed symbols).

The corrected data in panel A were used to quantify the signal imprecision, see panels B and C. The CV_S vs. $N_{particles}$ curve in panel B scales as $1/x^{1/2}$, in agreement with Poisson statistics. The CV_S vs. $t_{sampling}$ curve scales as $1/x^{1/2}$ for t < 10 s and levels off for longer times. This indicates that Poisson statistics dominate the variations at short times and that other sources of variation dominate on time scales larger than 10-100 s.

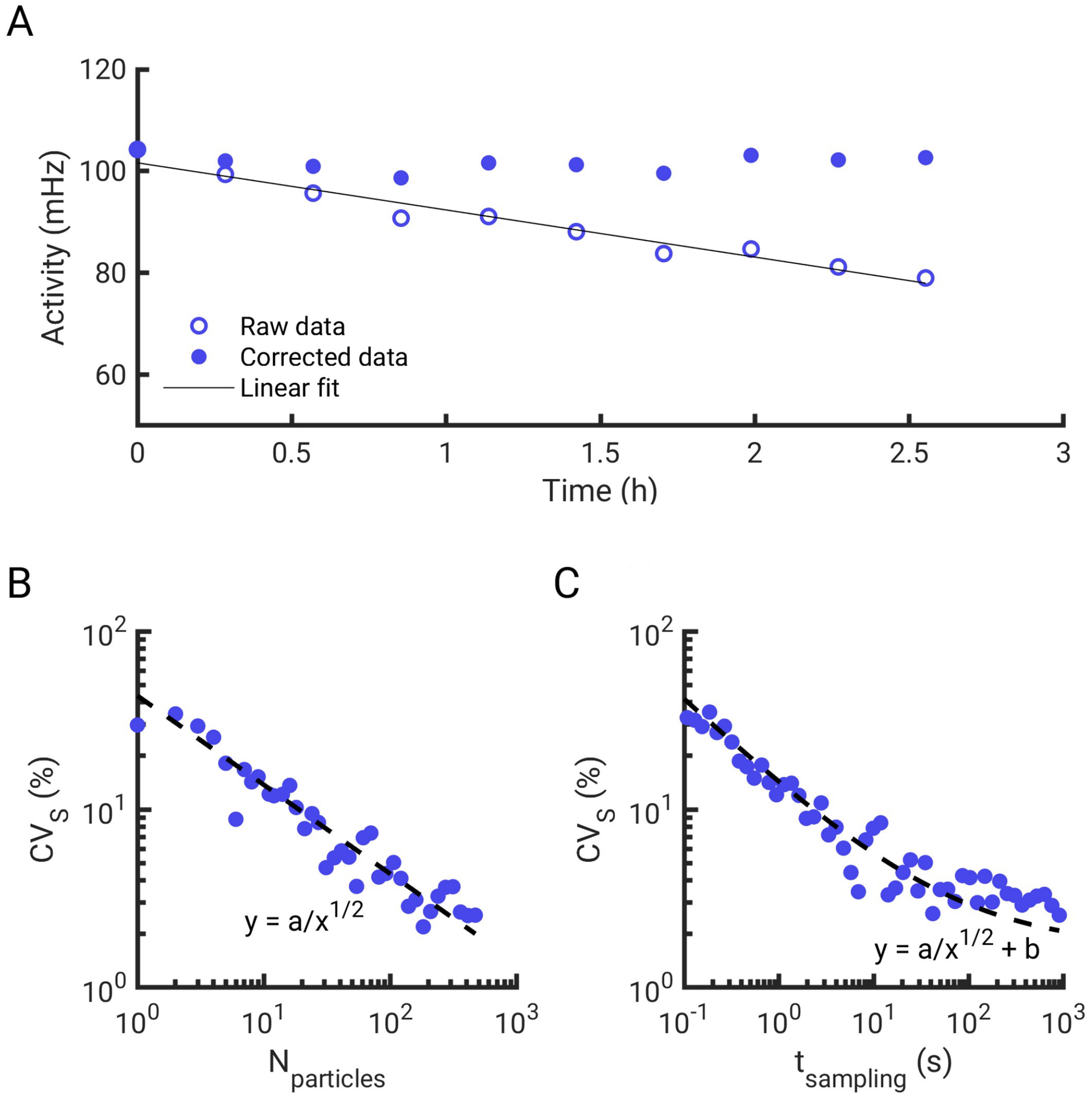

Supplementary Figure 19 | Analysis of signal imprecision (CV_S) in BPM. (A) Measured switching activity. The blue open data points represent the raw experimental data, the blue filled data points show the data after normalization using a linear fit (black line). (B) CV_S as function of the number of particles. The data are fitted with a curve of the form $y = a/x^{1/2}$. (C) CV_S as function of the sampling time. The data are fitted with a curve of the form $y = a/x^{1/2} + b$. Source data are provided as a Source Data file.

To further understand time-dependent effects on the signal imprecision, we analyzed each individual 15 min measurement and divided them into 10 blocks of 90 s to minimize temporal drift. Supplementary Figure 20 shows the measurements from Supplementary Figure 19 separated into 10 blocks plotted over time (left) and as histograms (right). The distributions of activity values appear narrow for all blocks except the first. The data in the first block (t = 0) shows an upward trend as a function of time, caused by the kinetics of the biomolecular reaction, which explains the broad spread of activity values.

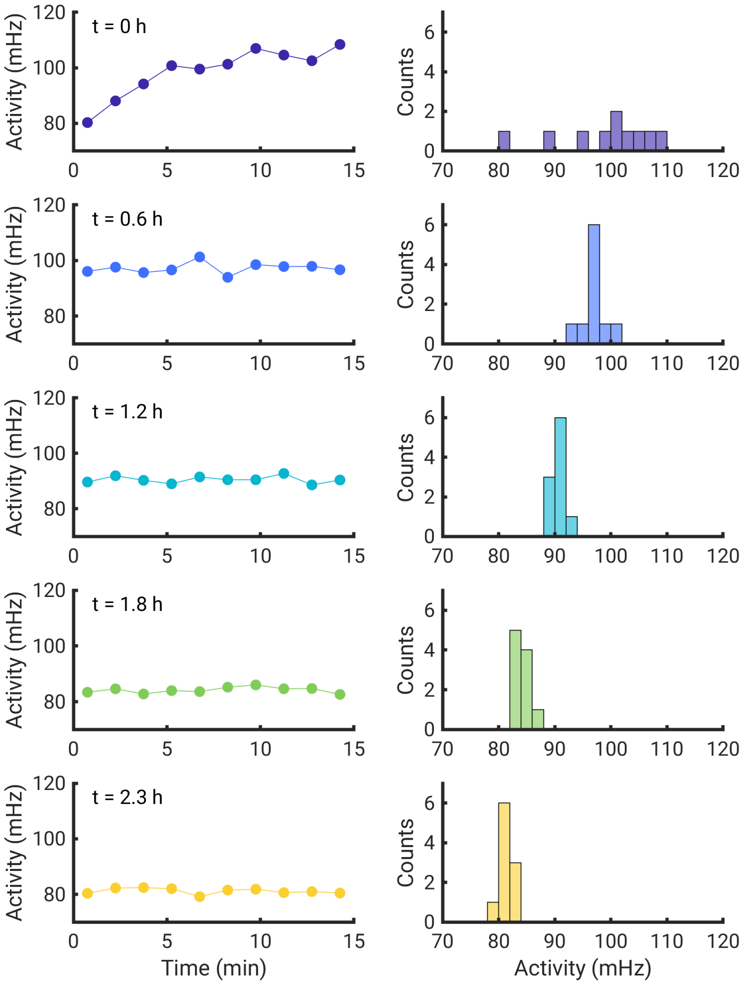

Supplementary Figure 20 | Distribution of activity values per measurement. Selected measurements in Supplementary Figure 19 were divided into 10 blocks, and the switching activity was recalculated for each block. The left plots show the activity as function of the time within a single measurement (15 min in total). The right plots show the data plotted as a histogram. Source data are provided as a Source Data file.

Supplementary Figure 21 shows the CV_S characteristics calculated from each measurements using the 10 blocks. All curves follow the expected $1/x^{1/2}$ scaling, indicating that the BPM sensor operates in the Poisson-limited regime. The t = 0 curve deviates at long time scales, which is attributed to the non-equilibrium of the sensor.

In conclusion, the analysis of experimental BPM data shows that the BPM signal imprecision curves as a function of the number of particles and as a function of the signal sampling time are dominated by Poisson statistics. Deviations are seen on long time scales, which is attributed to other sources of variability in the sensor.

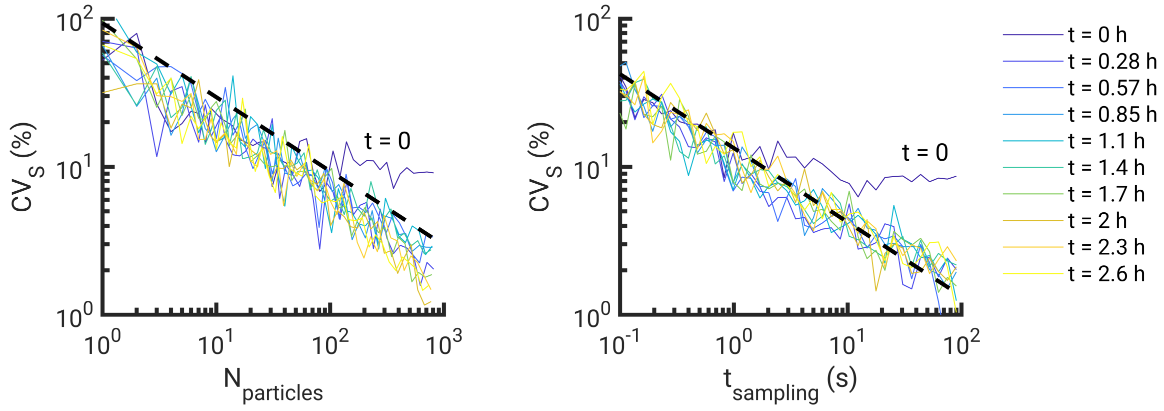

Supplementary Figure 21 | Analysis of signal imprecision (CV_S) in BPM experiments. The colors of the curves correspond to the colors used in Supplementary Figure 20. Left: CV_S as function of the number of particles. The 10 curves correspond to the 10 data points measured in Supplementary Figure 19. The dashed black line indicates the $y \propto 1/x^{1/2}$ scaling. The deviating line in dark blue corresponds to the first measurement in the experiment (t = 0). Right: CV_S as function of the sampling time. The 10 curves correspond to the 10 data points measured in Supplementary Figure 19. The dashed black line indicates the $y \propto 1/x^{1/2}$ scaling. The deviating line in dark blue corresponds to the first measurement in the experiment (t = 0). Source data are provided as a Source Data file.

6.5. Determination of LOQs

The Monte Carlo modelling was used to numerically obtain the standard deviation of the sensor response as function of the analyte concentration, see Supplementary Figure 22A. The noisiness of the determined values relates to the uncertainty in determining the imprecision, caused by limited statistics, as explained in Supplementary Note 6.2. In order to clarify how the LOQ scales with different design parameters, the imprecision curves were fitted and the fits were used in subsequent calculations, as explained in this Supplementary Note.

For concentrations below the EC_{50}, the standard deviation scales with the square root of the concentration. This is in accordance with Poisson statistics, since the standard deviation of a Poisson-distributed variable scales with the square root of its mean, and the sensor signal scales linearly with low concentrations. Because the analysis focuses on the sensor behavior at low analyte concentrations, the trend can be approximated with a fit of the form $y = a \cdot x^{1/2}$.

To use equation (6.5) for calculating the concentration imprecision CV_C, we used a 4-parameter logistic fit (equation (6.1)) and dose-response data generated by the analytical model. The binder numbers were Poisson-distributed, therefore the dose–response curve of the total sensor was obtained by averaging the individual responses of all single nanoswitches, each with their own combination of PSBs and SSBs given by a Poisson distribution.

Supplementary Figure 22B shows the nanoswitch data calculated using the Monte Carlo model (symbols), calculated analytically (colored lines), and fitted using equation (6.1) (black lines). The fits are good for concentrations below the EC_{50}, but not for higher concentrations, due to the high dose hook effect, see Fig. 2d.

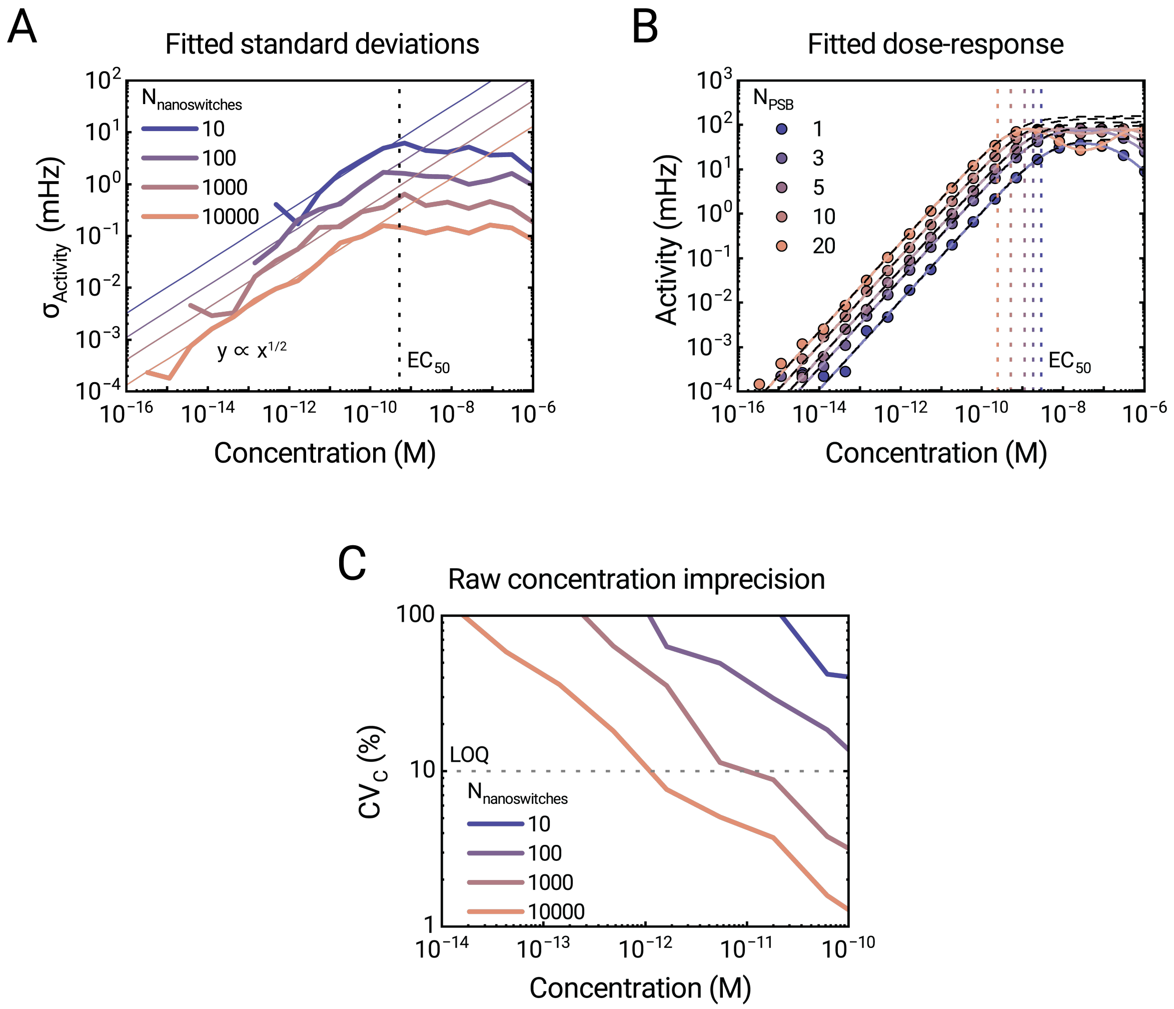

Supplementary Figure 22 | Determination of LOQs. (A) The thick lines show the simulated standard deviations obtained via Monte Carlo, the thin lines are fits of the form $y = a \cdot x^{1/2}$. The data originates from the same dataset used in Fig. 4d and e. (B) Nanoswitch dose-response curves. The circles are the data simulated via Monte Carlo, the colored lines are the data obtained via the analytical model, the black dashed lines are the 4-parameter logistic fits, the colored dotted lines are the EC_{50} values obtained from the analytical model. (C) Raw CV_C profiles, obtained from the raw standard deviations in panel A, and using equation (6.5). Source data are provided as a Source Data file.

The CV_C was obtained by projecting the signal imprecision on the concentration axis, as explained in Fig. 4a and equation (6.5). The resulting CV_C profiles are shown in Fig. 4d of the main text. Linear interpolation was then used to find the concentration at which the CV_C equals 10%, corresponding to the limit of quantification. Supplementary Figure 22C shows the CV_C profiles calculated using the raw simulated standard deviations shown in panel A. The lines appear to be spaced evenly apart, in agreement with the data shown in Fig. 4d in the main text.

7. Nanoswitch including non-specific interactions

The RNM described in the body text describes sandwich-based nanoswitches with specific biomolecular interactions. In addition to specific interactions, the biosensors can exhibit non-specific interactions, which give background signals and have noise contributions. This Supplementary Note describes a preliminary approach to include non-specific interactions in the model. Non-specific interactions are modelled as a two-state Poisson process that operates in parallel to the specific interactions. We study how the resulting concentration-independent switching signals affect the observed sensor response, the measurement precision, and the limit of quantification.

7.1. Modelling non-specific binding in reversible nanoswitches

Non-specific interactions are generic physicochemical forces such as hydrophobic interactions, hydrogen bonds, electrostatic and van der Waals forces that are not part of a specific inter-molecular interaction. Non-specific interactions can be weak and reversible, but can also be strong and non-reversible[11]. In biosensors, non-specific interactions cannot be avoided, but they can be suppressed by using anti-fouling coatings and blocking steps, for example.

Since non-specific interactions are heterogeneous and often not well-characterized, we describe these as general binding and unbinding process that operate in parallel to specific interactions. We model the non-specific binding (NSB) as a two-state Poisson process (unbound and bound) with respective kinetic rate constants, see Supplementary Figure 23A. The interactions only affect the nanoswitch binding state, meaning that specific interactions of the analyte with binder molecules and sandwich bond formations can still take place. The dwell times of the states are dictated by the effective non-specific association rate constant $k^*_{\mathrm{on,NSB}}$ and dissociation rate constant $k^*_{\mathrm{off,NSB}}$, both with unit s^{-1}.

Supplementary Figure 23B and 23C show dose-response curves for different kinetic rate constants, simulated via Monte Carlo. We assumed $k^*_{\mathrm{off,NSB}}$ to be constant. The values for $k^*_{\mathrm{on,NSB}}$ were chosen to achieve activity values that match experimental BPM data (background activity in the range ~10-20 mHz). The dynamic range of the sensor is reduced when NSB events are frequent, due to an increase in the background switching activity and a decrease in the maximum signal value, see Supplementary Figure 23C. The latter effect occurs because specific nanoswitch interactions are masked when the nanoswitch is non-specifically bound, lowering the number of observed specific switching events.

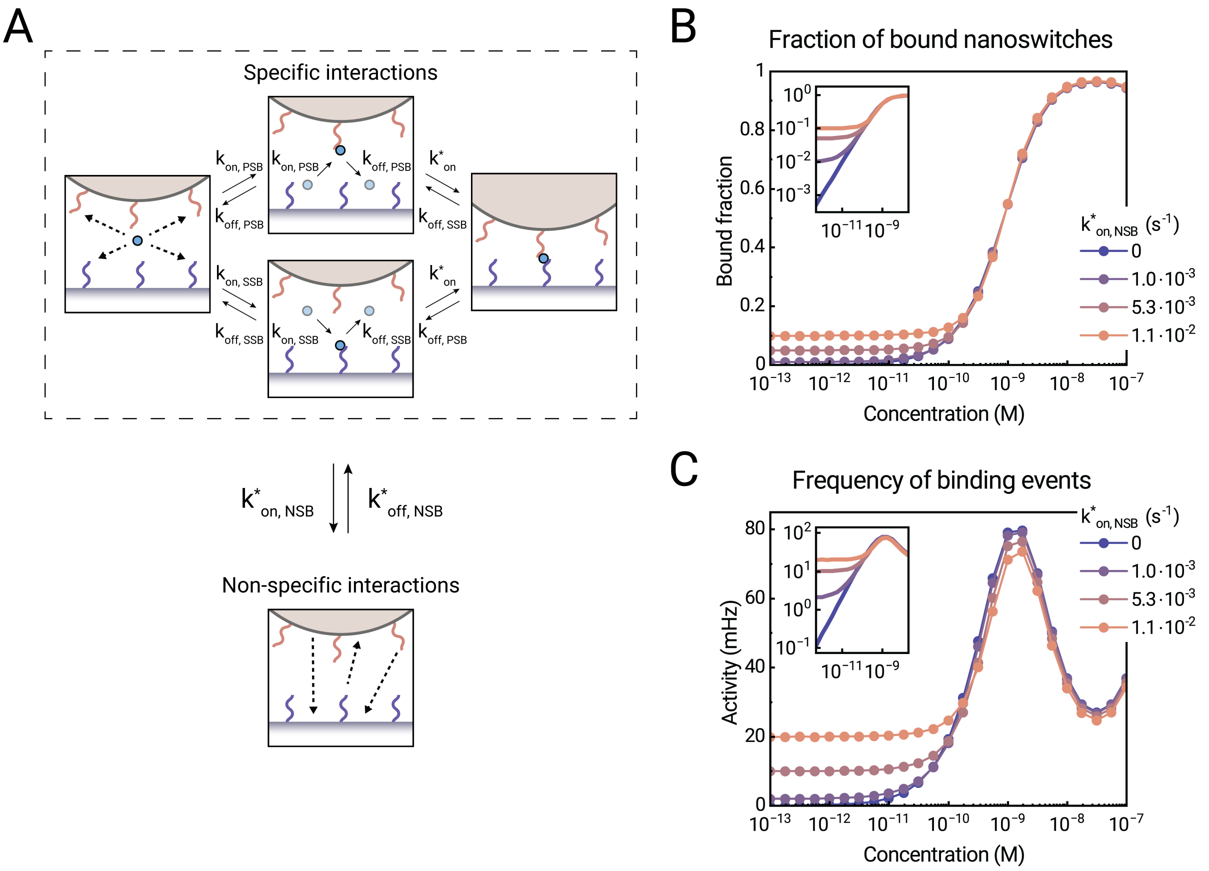

Supplementary Figure 23 | Modelling non-specific binding in reversible nanoswitches. (A) Reaction pathways in the simulation model. In parallel to the analyte reaction pathways, the particle probe can non-specifically interact with the surface with non-specific binding rate constants $k^*_{on,NSB}$ and $k^*_{on,SSB}$. The analyte binding events can still take place when a nanoswitch is non-specifically bound. (B) Response of the sandwich-based nanoswitch based on particle probe binding probability, simulated via Monte Carlo. The data was simulated for N_{PSB} = 10, N_{SSB} = 20, $k_{off,NSB}$ = 0.1 s^{-1}, and the rate constants shown in Table 1, and is an average of n = 15 simulation runs. The inset shows the same data plotted on a log-log scale. (C) Response of the sandwich-based nanoswitch based on observed binding and unbinding events, simulated via Monte Carlo. The data follows from the same dataset shown in panel B. The inset shows the same data plotted on a log-log scale. Source data are provided as a Source Data file.

7.2. Measurement imprecision and LOQ

Supplementary Figure 24A and 24B show how the presence of NSB events affects the signal imprecision, simulated for four different values of $k^*_{on,NSB}$, evaluated at a concentration near the EC_{50} (~320 pM). The CV_S scales as $1/\sqrt{N_{nanoswitches}}$ for all values of $k^*_{on,NSB}$. Fluctuations caused by NSB are independent across particles and therefore average out, leaving the $1/\sqrt{N_{nanoswitches}}$ scaling intact.

In contrast, when simulated as a function of the sampling time, the curves deviate from the expected $1/\sqrt{t_{sampling}}$ scaling as NSB events become more frequent, instead showing a slope shallower than -1/2 in the log-log graph, see Supplementary Fig. 24B. The lower slope is caused by the non-specifically bound states, during which specific binding events cannot contribute to the signal. The fraction of time during which the nanoswitch is free from NSB is given by:

$$f_{free\ from\ NSB} = \frac{k^*_{off,NSB}}{k^*_{on,NSB} + k^*_{off,NSB}}, \tag{7.1}$$

meaning that (1) the number of specific events grows with $f_{free} \cdot t_{sampling}$ and (2) events become more correlated (in addition to what was previously discussed in Supplementary Note 6.3), since specific events can only occur when the particle is not non-specifically bound. Only when $t_{sampling}$ is very large, enough statistics can be collected for the $1/t_{sampling}^{1/2}$ scaling to be restored.

Supplementary Figure 24C shows how the CV_C scales with concentration. The CV_C values were obtained from the raw σ_S values and equation (6.5). In the absence of NSB, the CV_C scales as $1/\sqrt{C}$, see Fig. 4d and the blue line in Supplementary Figure 24C. By introducing NSB, the scaling changes to $CV_C \propto 1/C$. In conditions where σ_S is dominated by Poisson noise from NSB events, the σ_S parameter becomes independent of the analyte concentration; in that limit, equation (6.6) indicates that $CV_C \propto \sigma_S/C \propto 1/C$.

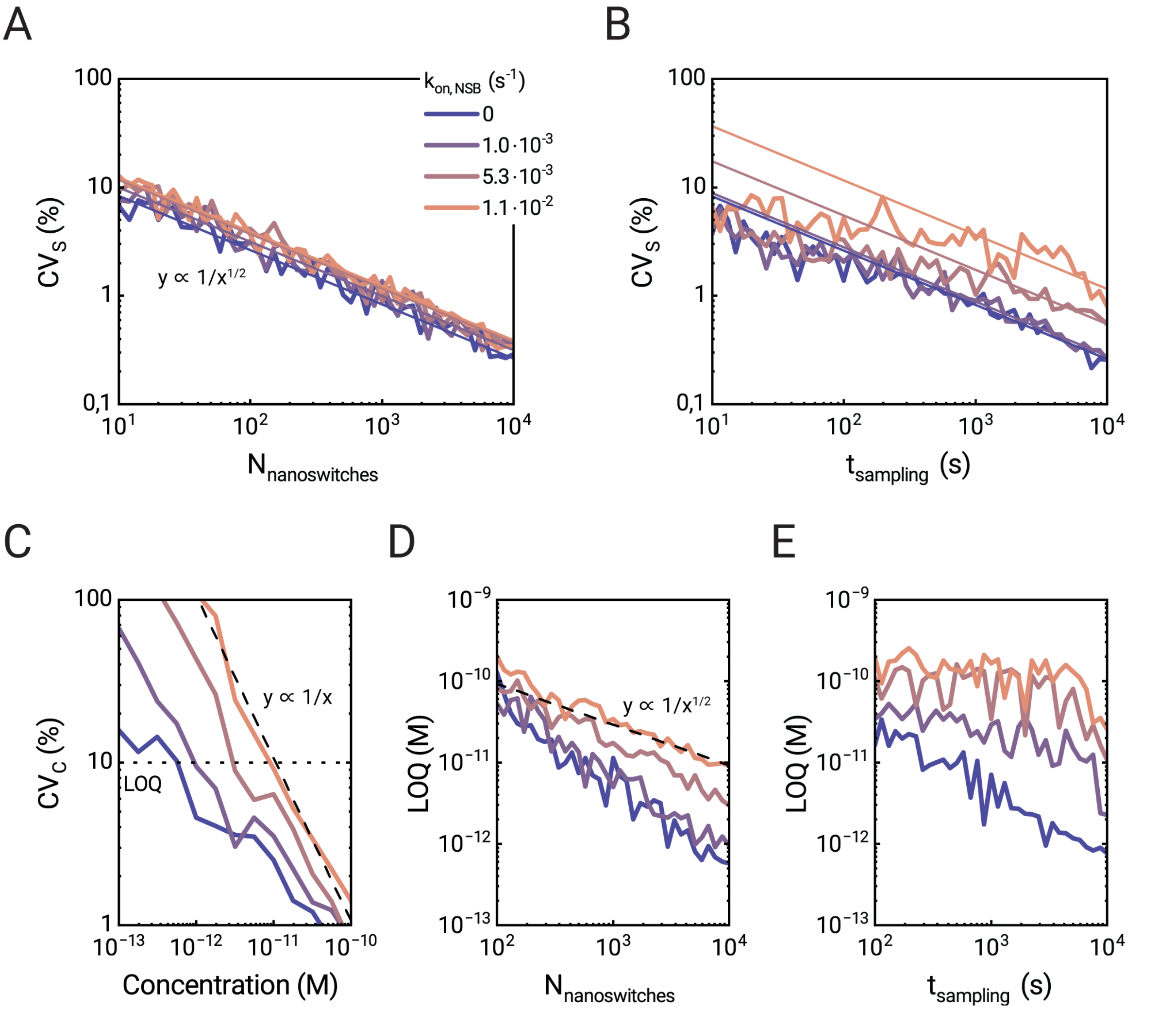

Supplementary Figure 24 | Measurement noise and sensor precision with non-specific binding. (A) Signal imprecision as function of the number of nanoswitches. The lines correspond to different values of $k_{on,NSB}$. The data was simulated for an analyte concentration of [A] = $3.2 \cdot 10^{-10}$ M, N_{PSB} = 20, N_{SSB} = 20, $t_{sampling}$ = 15 min, $k_{off,NSB}$ = 0.1 s^{-1} and the same kinetic rate constants shown in Table 1, and was obtained from n = 15 simulation runs. (B) Signal imprecision as function of the sampling time. The data was simulated for $N_{nanoswitches}$ = 1.000, all other simulation conditions are identical to panel A. (C) Concentration imprecision as function of the concentration, for different values of $k_{on,NSB}$. All simulation conditions are identical to panel A. (D) Limit of quantification as function of the number of nanoswitches. All simulation conditions are identical to panel A. (E) Limit of quantification as function of the sampling time. All simulation conditions are identical to panel B. Source data are provided as a Source Data file.

The modified scaling of σ_S has consequences for the scaling of the limit of quantification (LOQ), as shown in Supplementary Box 1 and Supplementary Figure 24D for $N_{nanoswitches}$. When σ_S is dominated by non-specific interactions, the LOQ scales with $1/N_{nanoswitches}^{1/2}$ instead of $1/N_{nanoswitches}$, leading to an increase by about an order of magnitude when $k^{*}_{on,NSB}$ is high (~$1.1 \cdot 10^{-2}$ s). Supplementary Figure 24E shows how the scaling of the LOQ with $t_{sampling}$ is affected by the modified σ_S scaling. The LOQ hardly depends on $t_{sampling}$ when $k^{*}_{on,NSB}$ is high, due to the combined effect of the burst-like distribution of events at low $t_{sampling}$ and the fast scaling of CV_C with the concentration. Tuning $N_{nanoswitches}$ remains a practical method to reduce the LOQ, because particles are independent and averaging more nanoswitches suppresses noise from stochastic sources such as NSB. The scaling laws are summarized in Supplementary Table 5.

Supplementary Box 1 | Scaling laws of the LOQ.

Assume a sensor with $N_{nanoswitches}$. The sensor signal S is expressed as the average number of switching events per nanoswitch per unit time. The total number of switching events recorded in a time period of $t_{sampling}$ over all nanoswitches is N_{events}. This gives for the sensor signal:

$$S = \frac{N_{events}}{N_{nanoswitches} \cdot t_{sampling}}.$$

Assume that the $N_{nanoswitches}$ is deterministic and accurately known. In contrast, the N_{events} is a stochastic parameter with intrinsic variability. Under Poisson statistics, the variability of N_{events} equals $N_{events}^{1/2}$. Therefore, the variability of the sensor signal σ_S is:

$$\sigma_S = \frac{\sqrt{N_{events}}}{N_{nanoswitches} \cdot t_{sampling}}.$$

N_{events} depends on $N_{nanoswitches}$ and on the analyte concentration C. In case of independent nanoswitches, N_{events} scales linearly with $N_{nanoswitches}$. In case of a linear dose-response relationship, which is the case in affinity-based sensing when concentrations are low, N_{events} scales linearly with C. With these inputs, we find:

$$\sigma_S \propto \frac{\sqrt{N_{events}}}{N_{nanoswitches} \cdot} \propto \frac{\sqrt{N_{nanoswitches} \cdot C}}{N_{nanoswitches}} = \sqrt{\frac{C}{N_{nanoswitches}}}.$$

Combined with Eq. (6.6), we find the scaling behavior of the concentration imprecision:

$$CV_C \propto \frac{\sigma_S}{C} = \frac{1}{\sqrt{N_{nanoswitches} \cdot C}}.$$

The LOQ corresponds to the concentration where the CV_C has a fixed value, typically 10%. Given the previous expression for the CV_C, a constant CV_C value means that the product $N_{nanoswitches} \cdot$ LOQ is constant. Therefore, the LOQ scales with $1/N_{nanoswitches}$.

In case the signal variability is dominated by non-specific interactions at low concentrations, then N_{evemts} is independent of the analyte concentration, but still scales with $N_{nanoswitches}$:

$$\sigma_S \propto \frac{\sqrt{N_{nanoswitches}}}{N_{nanoswitches} \cdot} = \frac{1}{\sqrt{N_{nanoswitches}}} \implies CV_C \propto \frac{1}{C\sqrt{N_{nanoswitches}}}.$$

A constant CV_C value then means that $N_{nanoswitches}^{1/2} \cdot$ LOQ is constant, meaning that the LOQ now scales with $1/N_{nanoswitches}^{1/2}$.

A similar derivation can be done for the scaling of the LOQ with the measurement time $t_{sampling}$, since N_{events} scales linearly with $t_{sampling}$ for a one-step Poisson process.

The results show that the LOQ can be reduced by suppressing NSB. The simulations of a nanoswitch with $k^*_{on,NSB}$ = 10^{-3} s^{-1} yield NSB-induced background activity signals of 2 mHz, as shown in Supplementary Fig. 20C. A sensor consisting of 10^4 of such nanoswitches has an LOQ of about 1 picomolar, as shown in Supplementary Figure 24D. This establishes a clear target for future NSB-suppressing strategies, such as the development of low-fouling and blocking layers, aiming to achieve reversible nanoswitch sensors for the continuous monitoring of low-picomolar biomarker concentrations.

Supplementary Table 5 | Summary of scaling laws.

Input parameter	Output parameter	Without NSB	With NSB
Analyte concentration	σ_S	$x^{1/2}$	-
	CV_C	$1/x^{1/2}$	1/x
$N_{nanoswitches}$	CV_S	$1/x^{1/2}$	$1/x^{1/2}$
	LOQ	1/x	$1/x^{1/2}$
$t_{sampling}$	CV_S	$1/x^{1/2}$	-
	LOQ	1/x	-

Supplementary References

1. Visser, E. W. A., Yan, J., Van IJzendoorn, L. J. & Prins, M. W. J. Continuous biomarker monitoring by particle mobility sensing with single molecule resolution. *Nat. Commun.* **9**, (2018).

2. Yan, J., Van Smeden, L., Merkx, M., Zijlstra, P. & Prins, M. W. J. Continuous Small-Molecule Monitoring with a Digital Single-Particle Switch. *ACS Sens.* **5**, 1168–1176 (2020).

3. Bergkamp, M. H., Cajigas, S., van IJzendoorn, L. J. & Prins, M. W. J. High-Throughput Single-Molecule Sensors: How Can the Signals Be Analyzed in Real Time for Achieving Real-Time Continuous Biosensing? *ACS Sens.* **8**, 2271–2281 (2023).

4. Lubken, R. M., Bergkamp, M. H., de Jong, A. M. & Prins, M. W. J. Sensing Methodology for the Rapid Monitoring of Biomolecules at Low Concentrations over Long Time Spans. *ACS Sens.* **6**, 4471–4481 (2021).

5. Lubken, R. M., De Jong, A. M. & Prins, M. W. J. Multiplexed Continuous Biosensing by Single-Molecule Encoded Nanoswitches. *Nano Lett.* **20**, 2296–2302 (2020).

6. Lubken, R. M., De Jong, A. M. & Prins, M. W. J. How Reactivity Variability of Biofunctionalized Particles Is Determined by Superpositional Heterogeneities. *ACS Nano* **15**, 1331–1341 (2021).

7. Vu, C., Yan, J., de Jong, A. M. & Prins, M. W. J. How Highly Heterogeneous Sensors with Single-Molecule Resolution can Result in Robust Continuous Monitoring Over Long Time Spans. *Advanced Science* **12**, 2412181 (2025).

8. Bergkamp, M. H., Van IJzendoorn, L. J. & Prins, M. W. J. Real-Time detection of state transitions in stochastic signals from biological systems. *ACS Omega* **6**, 17726–17733 (2021).

9. Joint Committee for Guides in Metrology (JCGM). *Evaluation of Measurement Data - Guide to the Expression of Uncertainty in Measurement*. (2008). doi:https://doi.org/10.59161/JCGM100-2008E.

10. Cajigas, S., De Jong, A. M., Yan, J. & Prins, M. W. J. Molecular Origins of Long-Term Changes in a Competitive Continuous Biosensor with Single-Molecule Resolution. *ACS Sens.* **9**, 3520–3530 (2024).

11. Frutiger, A. *et al.* Nonspecific Binding - Fundamental Concepts and Consequences for Biosensing Applications. *Chem. Rev.* **121**, 8095–8160 (2021).
