# Supplementary material for: Single-molecule readout of reversible nanoswitches enables continuous monitoring of low biomarker concentrations
Source: Nat Commun. 2026 Apr 18;17:5412. doi: 10.1038/s41467-026-71690-8 (PMC13280497; doi:10.1038/s41467-026-71690-8)
Supplement: Supplementary file 3 — Transparent Peer Review file [file 41467_2026_71690_MOESM3_ESM.pdf]

nature portfolio

Peer Review File

Single-molecule readout of reversible nanoswitches enables continuous monitoring of low biomarker concentrations

Corresponding Author: Professor Menno Prins

This file contains all reviewer reports in order by version, followed by all author rebuttals in order by version.

Version 0:

Reviewer comments:

Reviewer #1

(Remarks to the Author)
In this manuscript, the authors present a theoretical framework for understanding continuous sensors with single binding event resolution in single binder, sandwich pair, and multivalent sandwich formats to illuminate how such sensors can achieve low pM level detection rapid equilibration on the order of 10's of minutes. This work addresses a longstanding challenge in continuous affinity-based molecular sensors in which high affinity receptors are limited by slow associated off-rates and slow equilibration in sensors based on bulk ensemble readouts.

Specifically, the authors present a framework that enables the use of lower affinity (faster equilibrating) receptors in a sensor with single binding event resolution to circumvent the challenges of bulk sensors by utilizing the information of single molecule level binding events. The authors examine the effects of the number of probe- and surface-side binders and binder off-rate on sensor performance in terms of equilibrium fraction bound and activity (related to number of binding and unbinding events) using a geometric model that can generalize to any number of binders. The authors take this analysis further by examining how the previously mentioned factors as well the number of nanoswitches observed, the sampling time, and concentration of target molecule studied inform the coefficient of variance (CV) and limit of quantification (LOQ) of the sensor's response.

Overall, this work is well-grounded in first principles, clearly written and offers an insightful approach for low-abundance continuous molecular monitoring using single molecule resolution to address many of the challenges faced by traditional bulk readout. Although further experimental validation with actual binders would have made the work more compelling, I feel that the current theoretical insights offer sufficient value for publication after minor revisions (below).

Issues to be resolved:
The bimodal activity response of the multivalent nanoswitch in Figure 2 could be clarified further. Specifically, what is causing the second peak at higher concentrations? Has this behavior been observed experimentally?

The authors show that the signal imprecision scales with the inverse square root of the number of nanoswitches and total sampling time (Fig 4b, c). With single-molecule detection, this is indeed expected from Poisson statistics. However, the LOQ scales with the inverse of these parameters (Fig 4e) instead of the square root. This is quite surprising, since at low concentrations, the signal should scale linearly with the concentration.

The authors describe the process to find the concentration imprecision from the signal imprecision (Fig 4a, SI section 6 etc), but they don't explain why the scaling is different, nor is there an analytical expression that directly shows the inverse scaling. This needs to be addressed analytically and intuitively, rather than just with simulation results.

Errors:
The color scheme for Figure 2 could be altered such that the colors of the PSB (orange) and SSB (blue) are not matching the color of the plot lines to enhance clarity.

Fig 2b – Seems like kon PSB and kon SSB are repeated twice in the two boxes in the middle instead of koff PSB and koff

SSB

Line 349 – “on the surface” is misplaced in the sentence.

SI line 158 – Should be τbinding

(Remarks on code availability)

Reviewer #2

(Remarks to the Author)
The article by Vu and colleagues „Single-molecule readout of reversible nanoswitches will enable continuous monitoring of low-picomolar biomarker concentrations“ provides a model and associated simulations to anticipate the outcome of single-molecule based sensors to measure the concentration of analytes at picomolar concentrations. The manuscript explores the limitation and requirements a potential sensor setup needs to provide in order to facilitate readout in the anticipated concentration regime. With this, fine-tuning of novel biosensors could become more robust, since the necessary requirements could be known a priori, alleviating the need of frequent trail-and-error enhancements. Given that, the scope of the work is appropriate for Nature Communications and would be of interest to broad scientific audience. The manuscript appears structured and the figures are of high quality. The main text is accompanied by an extensive supplemental material explaining the model, providing control simulations and experimental results. The manuscript seems also closely related to a recent publication of the group (https://doi.org/10.1002/anie.202517725).
Nevertheless, in its current state the manuscript still contains several inconsistencies that need to be addressed before the publication and major revisions are recommended. There major critiques:
1. The model presented here assumes that in the multivalent sensor designs all interactions can be treated independently which is most likely not the case in most multivalent sensors (especially with N values as high as the ones simulated here). In the model authors include the association rate (106 M-1s-1) that is much higher than dissociation rate (10-2 – 10-1), therefore, one would expect that the prior binding events would influence the following ones as after the first sandwich bonds are formed the sensor conformation to form the next ones would be more favorable. This would lead to cooperative behavior and sharper dose response curve, which is in fact observed in the experimental data (both in this work, as well as previous work). Authors do not address this aspect sufficiently in the manuscript and treating the binding sites as independent does not simulate a realistic behavior of the sensor (unless this can be disputed with experimental data fitted using the proposed model to account for the observed behavior).
2. Along the same lines, authors claim that previous work and simulations included here challenge the notion that high affinity binders are necessary to effectively capture analytes at low concentrations and propose that low-affinity interactions can achieve continuous detection of analytes at low-picomolar concentrations. This argumentation is a bit misleading conceptually as using a high number of low-affinity interactions do in fact turn a low-affinity sensor into a high-affinity sensor – in this case comparing the apparent affinities of the sensors (K1/2 values) would be more appropriate. What would be the K1/2 values of the designed sensors and how would this compare to a typical sensor with using a high affinity interaction? Also given that, one should also be more transparent regarding reversibility of the sensor – given that all of these interactions add up the dissociation rate (opening) of the sensor might be relatively slow and it might be easier to detect the increase of the analyte concentration rather than decrease (something that is touched upon briefly in the previous work, however, not elaborated enough here or included in the model, discussed in the light of the model). Authors claim that one of the advantages of the multivalent sensors based on the low-affinity interactions is reversibility, however, the reversibility is not experimentally demonstrated and it is not elaborated what extent of reversibility one could in practice achieve if the low-affinity interactions add up to a high-affinity binding. These aspects should be addressed and clarified more critically.
3. While the model makes many generalized conclusions, the provided experimental data to support it is very limited, especially given that the assumptions in the model might not hold the realistic behavior of multivalent sensors (as per points above). Authors should provide more experimental data to support the claims that are made, going beyond one Figure in the SI. For the experimental data provided, it was not even clear if it was fitted with the model proposed and how it supported the model. More experimental data should be provided to support claims on effects of sampling, kinetics of the sensor etc. Moreover, the experimental data should cover the sample space of the simulation (or at least be close to it). The simulations included N from 1 to 10000, while the experimental data was provided for N from 1 to 10. Could the authors use the data published previously to support this model? Also, the dose response of the experimental data should be analyzed to evaluate the degree of cooperativity in given sensor design, comment how much the possible cooperativity effects influence the deviations from the model proposed. Does the model allow to include different association rates for different interactions to account for this cooperativity?
Other points that should be addressed, listed more specifically (in order of appearance, L=Line):
4. L1: Title: Please change the title to convey the simulation/modeling aspect of this publication, as the title is a bit misleading. “Simulating/Modelling single-molecule readout of reversible nanoswitches for continuous monitoring of low-picomolar biological analyte concentrations”.
5. L5: Arthur M. De Jong is missing the affiliation number (included in supplement). Also, no author contribution has been stated (L744 ff.).
6. L63ff: For transparency, one could use “we” since it is recent work from the same group. “Without applying any regeneration strategy” – the analyte was washed out using microfluidics.
7. L74ff: “We demonstrate … biomolecules”. Abstract binders were modeled and DNA hybridization was measured. It appears too general of a statement: either “we simulate” or “we demonstrate” and be specific of the target molecules used.
8. L94: Why choosing 106 binders exactly? Please comment. From an experimental viewpoint, the number of binders per

sensor will vary due to surface inhomogeneities. Can you simulate this by drawing from a population of “number of binders”. How does this influence the ultimate readout and sensitivity? You address this partially in Sup. Fig. 8.
9. L138: Can you comment on the selection of delay times for high and low affinity binders?
10. L140; L184: “respond instantaneously” – In the previous publication, systems needed to equilibrate for readout. Can you comment on this?
11. L193f: study dose-response – maybe “model” dose-response do indicate simulation and differentiate to the experimental data.
12. L221ff: Experimental results are mentioned and it is pointed to Figure 2 which shows a scheme rather than data. Also, experimental data is only in the supplements and no raw images and exemplary readouts are shown.
13. L224f: the model is applicable for other switches – a claim that conceptually holds true but is not yet proven.
14. L240ff, L291, L314: As per points 1 and 2 above, the biggest drawback of the used model and system is missing cooperativity between the binders and the ultimate readout. Omitting it for simplicity is removing a crucial aspect of real-world scenario, as only cooperativity will enable low-affinity binders to allow a “high-affinity” readout as shown in Fig 3a vs d.
15. L249-250, Table 1: why these specific dissociation rates were chosen for the probe-side and the surface-side binders? Was there a rationale that these rates would be different?
16. L258: 10 binders on each side, does this work with the sensor geometry and binding interface?
17. L264: Figure 2 c-d: A single binder does not necessary provide picomolar readout. Multiple binders induce two maxima when it comes to the readout (activity in this case). How do then decide which concentration is measured? For example, in the case of 100 binders and activity of 80 mHz could mean concentration of either 10-4M or 10-10M.
18. L249: “with on the surface only a single binder” – with only a single binder on the surface
19. L354: varying binder numbers tunes sensitivity. However, the number of binders per sensor will usually not be known exactly. How does this affect the readout (see comment to L94)
20. L534: Regarding the experiment: how does temperature influence the concentration and interaction readout as DNA hybridization is temperature dependent.
21. L584: Emphasizing the usefulness of the presented model, however, no data availability statement, code repository and programming language is mentioned in the text.
For the provided supplementary information:
22. L92: As per point 3 above, model designed to probe independent events, however, cooperativity will most likely play a crucial role for the sensor readout. Could the model be revised to include cooperative binding/simulate the effects of cooperative binding?
23. L169: NB0 the number of binder molecules on the sensing surface. Does this include the surface of the bead as well, or only the flat surface of the sensor?
24. L208: Nbinders per side is 30, however once the bead is bound, a fraction of the binders will not be able to perform a sensor readout, since they might be on the volume-facing side of the bead. How one would account for that?
25. L214: intra-nanoswitch binding has not been studied extensively – but has it been studied? Would there be references that could help improve the model and put it in the perspective of other works?
26. L235: Sup. Fig 2C: blue unbound lifetimes are missing datapoints for the first 3 concentrations
27. L332f: Please state the fact of missing cooperative effects more clearly.
28. L463: Figure 6- concerning comment on L208. Is increasing the number of binders on the probe side also considering the geometric constrains of the sensor?
29. L500: Figure 7: Can you add a direct comparison between the model and the experimental data in one graph? Can you infer using you model how many binders were on the respective beads? Can you provide exemplary data images of the beads, the determination of the activity and the respective classification?
30. L707: 100 nanoswitches within 15 simulations, that contained 10 000 nanoswitches. Which is the correct number simulated?
31. L798: Fig 16 A. Why are the datapoints shifted in respect to time, since they were acquired every 15 minutes? B, C red open markings are described in the figure description, yet no markings are seen.
32. L805: temporal drift. Can you comment which kind of drift? Drift due to exposure and processing or drift of the sample itself?
33. L808: Describing the distribution as normally distributed appears stretched with the number of counts and bins.
34. L826: Fig 18. Can you provide a legend for the different colors in the same figure? This would improve its clarity.
35. L832: Adding to this chapter, could you also analyze the previous published system using the antibodies and lactoferrin using your model?
36. L898: Simulations using a background activity between 10-20 mHz would prevent the readout low picomolar analytes (see main figure 2d inset) as the activity itself will be above readout activity. Can you comment on this?
37. L952ff: Draws a conclusion about the nanoswitch sensors that would also fit the main text.
38. L978: Can you comment on the buoyancy of the polystyrene beads?
39. L1006: PSB? Did you mean PBS?
40. L1008: How did you validate the DNA loading on the beads?
41. L1010: PSB again
42. L1012: What is HS buffer?

(Remarks on code availability)
The code works with the examples provided. The functional code is tidy, comments and docstrings are appropriate, therefore the code is well documented.

Reviewer #3

(Remarks to the Author)
I co-reviewed this manuscript with one of the reviewers who provided the listed reports. This is part of the Nature Communications initiative to facilitate training in peer review and to provide appropriate recognition for Early Career Researchers who co-review manuscripts.

(Remarks on code availability)

Version 1:

Reviewer comments:

Reviewer #1

(Remarks to the Author)
The authors have satisfactorily addressed my concerns in this revision. I suggest publication of this manuscript as-is.

(Remarks on code availability)

Reviewer #2

(Remarks to the Author)
The revised article addresses many of the mentioned points mentioned in the initial review and integrated substantial changes to the main text and even more elaborations in the Supplementary information section. Since cooperativity is, as explained by the authors, not in scope of the current approach, I deem the article to be ready for acceptance after minor revisions due to small discrepancies.
1. In the answer to Reviewer 1, 2nd answer, SI Section 7.2 is mentioned, but does not exist in the submitted document. The Box 1 which is mentioned is however present.
2. Within the new Box1: The formular for the signal itself and its variability is the same. Later in the box, the variability is mentioned as the square root of the signal. This might be a typo.
3. On the answer to Reviewer 2, 3rd statement: Sup. Figure 8 is mentioned; however, the reference would be matching Figure 9, lower.
4. Arthur M. De Jong is still missing the affiliation, but has author contribution

(Remarks on code availability)
Was already assesed in the first submission, no problems detected with the code

Reviewer #3

(Remarks to the Author)
I co-reviewed this manuscript with one of the reviewers who provided the listed reports. This is part of the Nature Communications initiative to facilitate training in peer review and to provide appropriate recognition for Early Career Researchers who co-review manuscripts.

(Remarks on code availability)

Open Access This Peer Review File is licensed under a Creative Commons Attribution 4.0 International License, which permits use, sharing, adaptation, distribution and reproduction in any medium or format, as long as you give appropriate credit to the original author(s) and the source, provide a link to the Creative Commons license, and indicate if changes were made.
In cases where reviewers are anonymous, credit should be given to 'Anonymous Referee' and the source.
The images or other third party material in this Peer Review File are included in the article's Creative Commons license, unless indicated otherwise in a credit line to the material. If material is not included in the article's Creative Commons license and your intended use is not permitted by statutory regulation or exceeds the permitted use, you will need to obtain permission directly from the copyright holder.
To view a copy of this license, visit https://creativecommons.org/licenses/by/4.0/

Reviewer #1 (Remarks to the Author)

In this manuscript, the authors present a theoretical framework for understanding continuous sensors with single binding event resolution in single binder, sandwich pair, and multivalent sandwich formats to illuminate how such sensors can achieve low pM level detection rapid equilibration on the order of 10's of minutes. This work addresses a longstanding challenge in continuous affinity-based molecular sensors in which high affinity receptors are limited by slow associated off-rates and slow equilibration in sensors based on bulk ensemble readouts.

Specifically, the authors present a framework that enables the use of lower affinity (faster equilibrating) receptors in a sensor with single binding event resolution to circumvent the challenges of bulk sensors by utilizing the information of single molecule level binding events. The authors examine the effects of the number of probe- and surface-side binders and binder off-rate on sensor performance in terms of equilibrium fraction bound and activity (related to number of binding and unbinding events) using a geometric model that can generalize to any number of binders. The authors take this analysis further by examining how the previously mentioned factors as well the number of nanoswitches observed, the sampling time, and concentration of target molecule studied inform the coefficient of variance (CV) and limit of quantification (LOQ) of the sensor's response.

Overall, this work is well-grounded in first principles, clearly written and offers an insightful approach for low-abundance continuous molecular monitoring using single molecule resolution to address many of the challenges faced by traditional bulk readout. Although further experimental validation with actual binders would have made the work more compelling, I feel that the current theoretical insights offer sufficient value for publication after minor revisions (below).

Issues to be resolved:

The bimodal activity response of the multivalent nanoswitch in Figure 2 could be clarified further. Specifically, what is causing the second peak at higher concentrations? Has this behavior been observed experimentally?

- Answer: We agree that the origin of the two peaks in the simulated dose-response curves in Figure 2 can be further clarified. The peaks are caused by analyte-induced transitions between monovalent and multivalent binding regimes.
  Regarding the first peak (the peak at low concentrations): when the analyte concentration increases starting from zero, the activity signal initially increases because of the formation of monovalent bonds between particle and surface (left side of the first peak), and thereafter decreases because multivalent bonds are formed (right side of the first peak).
  Regarding the second peak: when the analyte concentration increases further, the occupation of both the probe-side and the surface-side binders by analyte increases. The occupation on both sides reduces the probability that molecular sandwich bonds are formed between particle and surface, causing a transition from multivalent binding to monovalent binding, and therefore an increase of the activity signal (left side of the second peak). As the analyte concentration further increases, binders on both sides become fully saturated with analyte, causing a complete loss of sandwich bonds and therefore a decrease of the activity signal to zero (right side of the second peak).
  In BPM experiments, analyte-induced transitions from monovalent to multivalent bonds have been observed (this paper, see Supplementary Figure 7B) as well as

from multivalent to monovalent bonds (Ref. 34, Vu et al. *Adv. Sci.* 2025). Ref. 34 describes a small-molecule competition-based BPM sensor, showing how saturation of binders by analyte reduces the switching activity signal of the sensor.

The present paper shows data of a ssDNA sandwich-based BPM sensor, which has a much higher density of binders on particle and surface than the competition sensor of Ref. 34. In the ssDNA sandwich sensor, analyte-induced transitions from monovalent to multivalent bonds have been observed (the first peak), but not from multivalent to monovalent (the second peak). Since the ssDNA sandwich-based was prepared with a high density of binders on particle and surface, we hypothesize that the second peak is not seen due to a multivalency-induced stabilization of the bound states of the particles. This topic will be further investigated in subsequent research.

- Change: Additional explanations have been added to the Results and to Section 4.4 of the Supplementary Information.

The authors show that the signal imprecision scales with the inverse square root of the number of nanoswitches and total sampling time (Fig 4b, c). With single-molecule detection, this is indeed expected from Poisson statistics. However, the LOQ scales with the inverse of these parameters (Fig 4e) instead of the square root. This is quite surprising, since at low concentrations, the signal should scale linearly with the concentration.

The authors describe the process to find the concentration imprecision from the signal imprecision (Fig 4a, SI section 6 etc), but they don't explain why the scaling is different, nor is there an analytical expression that directly shows the inverse scaling. This needs to be addressed analytically and intuitively, rather than just with simulation results.

- Answer: We agree with the reviewer that this scaling behavior can be explained more clearly.
- Change: We have expanded on this topic in the Results section and in the SI Section 7.2, see Box 1.

Reviewer #2 (Remarks to the Author)

The article by Vu and colleagues "Single-molecule readout of reversible nanoswitches will enable continuous monitoring of low-picomolar biomarker concentrations" provides a model and associated simulations to anticipate the outcome of single-molecule based sensors to measure the concentration of analytes at picomolar concentrations. The manuscript explores the limitation and requirements a potential sensor setup needs to provide in order to facilitate readout in the anticipated concentration regime. With this, fine-tuning of novel biosensors could become more robust, since the necessary requirements could be known a priori, alleviating the need of frequent trail-and-error enhancements. Given that, the scope of the work is appropriate for Nature Communications and would be of interest to broad scientific audience. The manuscript appears structured and the figures are of high quality. The main text is accompanied by an extensive supplemental material explaining the model, providing control simulations and experimental results. The manuscript seems also closely related to a recent publication of the group (https://doi.org/10.1002/anie.202517725).

Nevertheless, in its current state the manuscript still contains several inconsistencies that need to be addressed before the publication and major revisions are recommended. There major critiques:

1. The model presented here assumes that in the multivalent sensor designs all interactions can be treated independently which is most likely not the case in most multivalent sensors (especially with N values as high as the ones simulated here). In the

model authors include the association rate (106 M-1s-1) that is much higher than dissociation rate (10-2 – 10-1), therefore, one would expect that the prior binding events would influence the following ones as after the first sandwich bonds are formed the sensor conformation to form the next ones would be more favorable. This would lead to cooperative behavior and sharper dose response curve, which is in fact observed in the experimental data (both in this work, as well as previous work). Authors do not address this aspect sufficiently in the manuscript and treating the binding sites as independent does not simulate a realistic behavior of the sensor (unless this can be disputed with experimental data fitted using the proposed model to account for the observed behavior).'

- Answer: We agree that the topics of multivalency and cooperativity can be further clarified.

  The main aim of the paper is to study kinetic and stochastic properties of sandwich nanoswitches in the low-concentration regime, in order to study how the lower limit of quantification depends on sensor design parameters (see Fig. 4). The low-concentration regime refers to concentrations below the EC_{50} on the left side of the first peaks in the dose-response curves shown in Figs. 2 and 3. In this regime, bound fractions are smaller than unity, switching activities are well below A_{max}, and interactions between particle and surface are dominated by monovalent bonds. Even though the nanoswitches have high numbers of binders on particle and surface (up to N=100 is studied in the simulations), in the low-concentration regime there is no multivalent binding between particle and surface due to the very low fractional occupation of individual binders by analyte molecules.

  As the analyte concentration is increased above the low-concentration regime, multivalent bonds start to appear between particle and surface, which is illustrated in Supplementary Fig. 4 for example. Above the low-concentration regime, the bound fraction approaches unity and the activity signal shows a peak (the first peak).

  In the simulations, we modelled the kinetics of bond formation by assuming that all molecular interactions are independent. This is an approach in which potential cooperative and anti-cooperative effects are neglected. In a sandwich-based nanoswitch with on both sides multiple binders, cooperative as well as anti-cooperative effects can be present in principle, but the magnitudes of the effects and the net results are unknown and difficult to predict. An example of a potential cooperative effect is that the formation of a first sandwich bond brings probe and surface in closer proximity, which might cause an additional sandwich bond to form with a *higher* effective association rate than the first one. An example of a potential anti-cooperative effect is that the presence of a first sandwich bond hinders the translational and rotational freedom of the probe with respect to the surface, which can cause an additional sandwich bond to form with a *lower* effective association rate than the first bond if the respective binders are not directly aligned and oriented.

  The reviewer refers to slopes of experimental dose-response curves. Slopes equal to one would be in agreement with independent interactions. Slopes higher than one would indicate the presence of net cooperative effects. Experimental dose-response curves presented in earlier papers (Ref. 19 and 24, i.e., Visser et al., *Nat. Commun.* 2021 and Michielsen et al., *Angew. Chem. Int. Ed.* 2025) were fitted using logistic curves with slope powers close to unity. This indicates that those dose-response curves are not dominated by cooperative or anti-cooperative effects, because these would cause slope powers deviating from unity. Furthermore, in another paper (Ref. 34) we studied analyte-induced transitions between monovalent and multivalent binding in a competition-based BPM nanoswitch. Good correspondences were observed between experimental results and simulations using

a model with independent interactions. Therefore, also the study of Ref. 34 did not reveal clear cooperative or anti-cooperative effects.

The insets in Figs. 2c and 2d of the present manuscript show simulation results using the model with independent interactions. In the low concentration regime, the slopes of the simulated dose-response curves are equal to one, in agreement with the assumption of independent interactions.

Experimental data of a ssDNA sandwich-based BPM sensor are shown in Supplementary Fig. 9. The dose-response data is reproduced below, plotted for three different readout parameters:

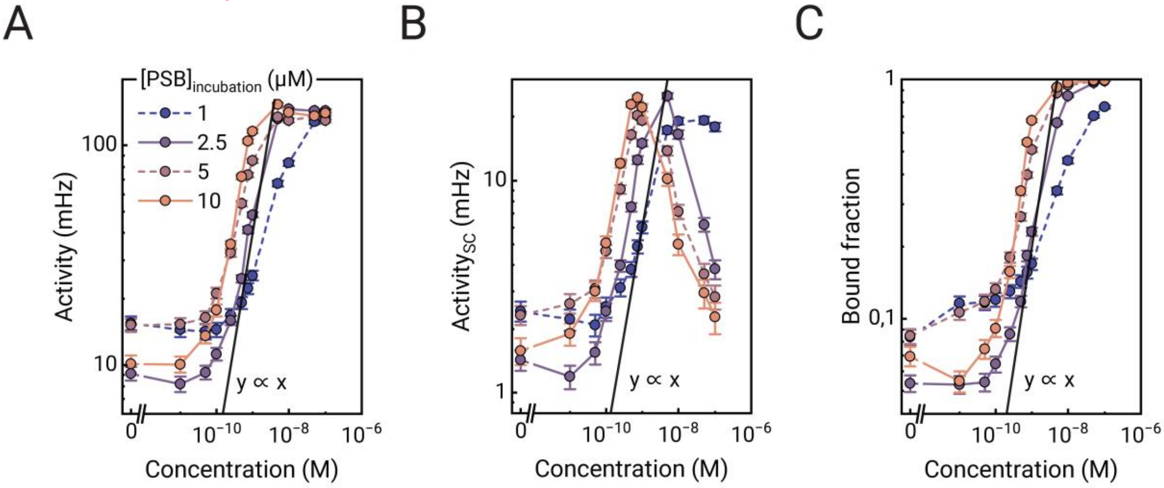

The data are plotted on log-log scales to highlight the slope behavior. In every panel, a black straight line indicates a slope with linear dependence, i.e., a slope power equal to one (y ∝ x). For all readout parameters, the graphs show that experiments with $[PSB]_{incubation}$ = 2.5, 5, and 10 μM give slopes close to one. The curves with the lowest density of binders on the particles ($[PSB]_{incubation}$ = 1 μM) have slopes that seem to be smaller than one for the Activity and Bound Fraction readout parameters, but less clearly for the $Activity_{SC}$ parameter. These data indicate that potentially a weak anti-cooperative effect is present for the lowest binder density on the particles, and no net (anti)cooperative effects are seen for higher binder densities on the particles. However, further work is needed to study the slopes of the dose-response curves and clarify their origins.

In the rate-based simulation model, cooperative and anti-cooperative effects can be implemented in various manners. For example, dependencies can be introduced of the rate parameters k^{*}_{on} and k_{off} on the number of formed sandwich bonds. Increasing k^{*}_{on} with the number of sandwich bonds could reflect a cooperativity, while increasing k_{off} could reflect an anti-cooperativity. However, since tested model descriptions are not yet available, the present paper studies a model with independent interactions. The model leads to predictions which can be tested in subsequent experiments. In parallel, the model will be further developed, e.g. using molecular dynamics or Brownian dynamics simulations.

- Change 1: We added clarifications about the model assumptions in the Discussion and Conclusion sections, and elaborated on the topic of cooperativity in Supplementary Note 4.7.
  Change 2: The model represents a rate-based description of nanoswitches. The present version of the model assumes independent interactions and does not yet include potential cooperative or anti-cooperative effects related to multivalent binding. The aim of the present study is not to study multivalency in detail, but to investigate

the behavior of nanoswitches with multiple binders at low analyte concentrations. Therefore, we propose to name the model ‘Rate-based nanoswitch model’ rather than ‘Rate-based multivalent nanoswitch model’.

2. Along the same lines, authors claim that previous work and simulations included here challenge the notion that high affinity binders are necessary to effectively capture analytes at low concentrations and propose that low-affinity interactions can achieve continuous detection of analytes at low-picomolar concentrations. This argumentation is a bit misleading conceptually as using a high number of low-affinity interactions do in fact turn a low-affinity sensor into a high-affinity sensor – in this case comparing the apparent affinities of the sensors (K1/2 values) would be more appropriate. What would be the K1/2 values of the designed sensors and how would this compare to a typical sensor with using a high affinity interaction? Also given that, one should also be more transparent regarding reversibility of the sensor – given that all of these interactions add up the dissociation rate (opening) of the sensor might be relatively slow and it might be easier to detect the increase of the analyte concentration rather than decrease (something that is touched upon briefly in the previous work, however, not elaborated enough here or included in the model, discussed in the light of the model). Authors claim that one of the advantages of the multivalent sensors based on the low-affinity interactions is reversibility, however, the reversibility is not experimentally demonstrated and it is not elaborated what extent of reversibility one could in practice achieve if the low-affinity interactions add up to a high-affinity binding. These aspects should be addressed and clarified more critically

- Answer: As mentioned in the answer to question 1 of Reviewer #2, the present manuscript focuses on the low concentration regime where switching activities are well below A_{max} and the nanoswitch behavior is dominated by monovalent binding. The nanoswitch sensors achieve a high sensitivity to analyte by the resulting counting statistics of switching events. At low analyte concentrations, switching events occur with high statistics due to the appearance and disappearance of monovalent bonds, caused by the high numbers of binder molecules in the nanoswitch. In the low-concentration regime, the switching statistics are caused by monovalent bonds and *not* by multivalent bonds in the nanoswitch.

  The sensing principle in the regime of monovalent binding can be illustrated by an example. Assume two nanoswitches that are equal but have different numbers of binder molecules on one side: the first nanoswitch has a single binder molecule on one side and the second has 10 binders. Suppose the nanoswitches are exposed to an analyte concentration of [A] = $0.1 \cdot K_D$. With this analyte concentration, the fractional occupancy of the binders by analyte is around 10% in the equilibrium condition, which means that every individual binder is occupied by analyte for about 10% of the time. Thus, the nanoswitch with 1 binder has a time-averaged occupancy of 0.1 analyte molecules, while the nanoswitch with 10 binders has a time-averaged occupancy of 1 analyte molecule. This means that the second nanoswitch is able to generate around 10 times more switching events, which increases its measurement precision and decreases its LOQ. This example shows that the LOQ of a nanoswitch sensor is decreased in the monovalent switching regime by increasing the number of binder molecules, without any need to invoke multivalent bonds.

  To further emphasize that the low quantification limits do not rely on multivalency, we refer to Supplementary Figure 6 (section 4.4 of the SI), that shows the distribution of the number of bonds over all nanoswitches when a nanoswitch sensor reaches its maximal signal response. The mean of the distribution tends to 1 for any set of nanoswitch parameters, meaning that - even at concentrations higher than the EC_{50}

of the sensor – monovalent binding still dominates the nanoswitch response, and the number of higher-order binding states remains low.

Regarding the topic of reversibility, the reviewer is correct that many sandwich bonds can increase the bound-state lifetime. This behavior is simulated in Supplementary Figure 17, in Section 6.3 of the SI. We simulated the bound-state lifetime distributions for an analyte concentration (1) much lower than the EC_{50}, (2) equal to the EC_{50} and (3) equal to the concentration where the maximal response is reached. For concentrations near or lower than the EC_{50}, which is the regime where we focus on for low-concentration detection, the distribution of bound-state lifetimes appears single-exponential with a mean bound-state lifetime corresponding to the binder with the faster k_{off} value. Here, the reversibility of the sensor is determined by the dissociation rate constants of individual molecular bonds and not by multivalent binding. For concentrations above the EC_{50}, the distribution tends to longer bound-state lifetimes due to multivalent binding, increasing the relaxation time of the sensor. However, as exemplified in Supplementary Figure 4, the extent of multivalency is very limited in the linear range of the dose-response curve of the sensor, leading to only about a twofold increase in the characteristic bound-state lifetime of the sensor.

- Change: We added clarifications about sensitivity and reversibility in the Results and in the SI. We added experimental proof of the reversibility of the BPM DNA sandwich sensor, showing six series of increasing and decreasing concentrations, see Supplementary Fig. 3.

3. While the model makes many generalized conclusions, the provided experimental data to support it is very limited, especially given that the assumptions in the model might not hold the realistic behavior of multivalent sensors (as per points above). Authors should provide more experimental data to support the claims that are made, going beyond one Figure in the SI. For the experimental data provided, it was not even clear if it was fitted with the model proposed and how it supported the model. More experimental data should be provided to support claims on effects of sampling, kinetics of the sensor etc. Moreover, the experimental data should cover the sample space of the simulation (or at least be close to it). The simulations included N from 1 to 10000, while the experimental data was provided for N from 1 to 10. Could the authors use the data published previously to support this model? Also, the dose response of the experimental data should be analyzed to evaluate the degree of cooperativity in given sensor design, comment how much the possible cooperativity effects influence the deviations from the model proposed. Does the model allow to include different association rates for different interactions to account for this cooperativity?

- Answer: The experimental data of the ssDNA sandwich-based BPM sensor supports the model predictions that the EC_{50} of the curves scale inversely with the number of binders and that Poisson noise is reduced by increasing the number of nanoswitches and by increasing the measurement time.

  The experimental data in Supplementary Fig. 8 was fitted with a 4-parameter logistic fit (panels A and C) and an inverse fit (panel B) to extract EC_{50} values. These showed the same inverse scaling with the number of binders as observed in the simulations ($y \sim 1/x$) . The data was not fitted with the rate-based model, we apologize for the confusion. The experimental data in Supporting Fig. 21 were fitted with inverse square-root functions ($y \sim 1/x^{1/2}$) to highlight the Poisson nature of the switching statistics. We conclude that the scaling behaviors of the experimental data and the simulations show good agreements.

  The input parameters used in the simulations can be related to the experimental conditions. Concerning the number of nanoswitches, we simulated between N = 1 –

10000. In BPM experiments, we work typically with between 100's and multiple 1000's of particles. We chose 10000 nanoswitches as an upper limit for the simulation because we have shown in previous work that we can simultaneously track 10000 particles in real-time, see Bergkamp et al. (2023) *ACS Sens.*, Ref. 36. In the BPM experiments of Supplementary Fig. 19, around 500 particles were tracked. Concerning the measurement time (t_{meas}), in BPM we typically use measurement durations of a minute to several minutes, but longer durations are definitely possible. As for the number of binders, we currently do not have a clear estimate of the absolute number of binders in the interaction volume between particle and surface. Using calculations based on the areal density of binders per surface and empirical studies on the accessibility of those molecules, we estimate that the number of binders that can participate in sandwich bond formation lies in the order of tens, see the second to last paragraph in the Results.

We have studied to a limited extent how performance characteristics of BPM sensors change with different design parameters. In Buskermolen et al., (2024) *Biosens. Bioelectron.,* a variant of the BPM sensor without the tether (fBPM) and with strong affinity binders was used. The data shows how increasing particle-side binder densities shifts the dynamic range of the sensor to lower concentration regimes. In Ref. 36, Bergkamp et al., (2023), *ACS Sens.*, a competition-based sensor for cortisol was used and scaling laws related to Poisson statistics were investigated. In Ref. 20, Yan et al., (2020), *ACS.Sens.*, a competition-based ssDNA sensor was studied including how signal variations scale with the number of events. More elaborate studies on how performance characteristics depend on design parameters will be performed in future work.

Regarding cooperativity, please see the answer to question 1 of Reviewer#2.

- Change: We address the topic of cooperativity in the Discussion & Conclusions and in the SI, additionally suggesting how this can be incorporated in the current model.

Other points that should be addressed, listed more specifically (in order of appearance, L=Line):

4. L1: Title: Please change the title to convey the simulation/modeling aspect of this publication, as the title is a bit misleading. "Simulating/Modelling single-molecule readout of reversible nanoswitches for continuous monitoring of low-picomolar biological analyte concentrations".

- Answer: We agree that the simulation/modelling aspect can be included in the title. We propose the following title: "Single-molecule readout of reversible nanoswitches enables continuous monitoring of low biomarker concentrations: A theoretical framework".
- Change: The title of the manuscript has been adjusted.

5. L5: Arthur M. De Jong is missing the affiliation number (included in supplement). Also, no author contribution has been stated (L744 ff.).

- Answer: We thank the reviewer for pointing this out.
- Change: We added information.

6. L63ff: For transparency, one could use "we" since it is recent work from the same group. "Without applying any regeneration strategy" – the analyte was washed out using microfluidics.

- Answer: We agree that this can be clarified.

- Change: We changed "without applying any regeneration strategy" to "in other words, with reversibility solely based on passive dissociation".

7. L74ff: "We demonstrate … biomolecules". Abstract binders were modeled and DNA hybridization was measured. It appears too general of a statement: either "we simulate" or "we demonstrate" and be specific of the target molecules used.

- Answer: We agree that this can be clarified.
- Change: We changed the sentence to "We demonstrate using this model that reversible…".

8. L94: Why choosing 106 binders exactly? Please comment. From an experimental viewpoint, the number of binders per sensor will vary due to surface inhomogeneities. Can you simulate this by drawing from a population of "number of binders". How does this influence the ultimate readout and sensitivity? You address this partially in Sup. Fig. 8.

- Answer: The number was chosen based on simulated numbers of binders per nanoswitch in the manuscript (10^1-10^2) multiplied by the maximum number of simulated nanoswitches (10^4).
  The reviewer is correct that the actual number of binders per sensor can vary, depending on the functionalization strategy. That is why in Figure 4 we simulate LOQs based on heterogeneous binder populations per surface (based on a Poisson distribution). In the regime of low binder densities (where the variation in the number of binders per nanoswitch is high), sensors with homogeneously distributed binders per nanoswitch would be more sensitive than heterogeneously distributed binders per nanoswitch, as these on average would have higher switching activities.
- Change: We added explanations in SI 4.7, Supplementary Fig. 8, now Supplementary Fig. 10.

9. L138: Can you comment on the selection of delay times for high and low affinity binders?

- Answer: Typical K_D values of antibodies designed to measure low concentrations (used in for example ELISAs) are in the nanomolar to picomolar range, with k_{off} values in the order of 10^{-3} s^{-1} or slower, given typical k_{on} values of 10^6-10^7 $M^{-1}s^{-1}$. A lower affinity binder would have a faster dissociation rate.
- Change: No change.

10. L140; L184: “respond instantaneously” – In the previous publication, systems needed to equilibrate for readout. Can you comment on this?

- Answer: We agree with the reviewer that this topic can be clarified. Figure 1a focuses on direct binding sensors to illustrate the concept of using a single-molecule readout versus an ensemble readout. The simulation shows that the frequency of association events responds instantaneously to changes in the analyte concentration. This means that a single-molecule biosensor that measures the frequency of association events can have a response time that is independent of the dissociation rate constant of the analyte-binder interaction (other factors such as mass transport, physical transduction, and signal processing will still cause a non-zero biosensor response time).
- Change: We added clarification in the Results and Discussion section.

11. L193f: study dose-response – maybe “model” dose-response do indicate simulation and differentiate to the experimental data.

- Answer: We both model and analyze dose-response characteristics, we believe that “study” captures both of these words.
- Change: No change.

12. L221ff: Experimental results are mentioned and it is pointed to Figure 2 which shows a scheme rather than data. Also, experimental data is only in the supplements and no raw images and exemplary readouts are shown.

- Answer: We meant to refer to Fig. 2a because the schematic is based on the BPM technology. Since the focus of the paper is to explain the theoretical framework and the scaling laws, we decided to put the experimental data in the SI.
- Change: We have clarified the Results section. We added raw microscopy images and an exemplary particle time trace in the SI, see Supplementary Fig. 2.

13. L224f: the model is applicable for other switches – a claim that conceptually holds true but is not yet proven.

- Answer: The rate-based nanoswitch model is designed to be generalizable. We expect that other research groups will use the model to study their nanoswitch systems.
- Change: We changed the wording.

14. L240ff, L291, L314: As per points 1 and 2 above, the biggest drawback of the used model and system is missing cooperativity between the binders and the ultimate readout. Omitting it for simplicity is removing a crucial aspect of real-world scenario, as only cooperativity will enable low-affinity binders to allow a “high-affinity” readout as shown in Fig 3a vs d.

- Answer: See previous answers related to cooperativity and multivalency.
- Change: See previous changes related to cooperativity and multivalency.

15. L249-250, Table 1: why these specific dissociation rates were chosen for the probe-side and the surface-side binders? Was there a rationale that these rates would be different?

- Answer: We chose asymmetrical dissociation rates to reflect differences in affinity when two binders target different epitopes of the same analyte molecule. We studied sensor properties in dependence of the respective values, see Fig. 3d-f.
- Change: No change.

16. L258: 10 binders on each side, does this work with the sensor geometry and binding interface?

- Answer: In BPM sensors, we estimate that the number of binder molecules that can participate in sandwich bond formation is in the order of tens, see the second to last paragraph in the Results.
- Change: No change.

17. L264: Figure 2 c-d: A single binder does not necessary provide picomolar readout. Multiple binders induce two maxima when it comes to the readout (activity in this case). How do then decide which concentration is measured? For example, in the case of 100 binders and activity of 80 mHz could mean concentration of either 10^{-4} M or 10^{-10} M.

- Answer: Similar to the Hook effect in ELISAs, a given signal value can relate to more than one concentration value. In applications, the order of magnitude of the analyte concentration is typically known, which solves the problem. Furthermore, the time profile of the signal can be used to distinguish between conditions, as described in literature. This topic is outside the scope of the present study.
- Change: No change.

18. L249: "with on the surface only a single binder" – with only a single binder on the surface

- Answer: We thank the reviewer for this correction.
- Change: We changed the text.

19. L354: varying binder numbers tunes sensitivity. However, the number of binders per sensor will usually not be known exactly. How does this affect the readout (see comment to L94)

- Answer: Nanoswitches based on DNA-origami allow for direct control over the placements and the number of binders per nanoswitch. Nanoswitches with random binder placements, as in the BPM experiments in this paper, have less control. Still, scaling laws can be studied by changing the incubation concentrations of binders during the functionalization process, see Supplementary Fig. 8. Random binder placements give inter-nanoswitch heterogeneities that have been studied, see Supplementary Fig. 11 and see Vu et al. (2025), *Adv. Sci.*
- Change: No change.

20. L534: Regarding the experiment: how does temperature influence the concentration and interaction readout as DNA hybridization is temperature dependent.

- Answer: Temperature changes can have two effects on a BPM sensor. First, as the reviewer mentioned, the DNA binding kinetics change: higher temperatures lead to shorter bound state lifetimes for both probe-side and surface-side binders, which can shift the dynamic range to higher concentrations and increase the number of events per bound DNA molecule. Second, higher temperatures lead to faster Brownian motion of the probe as it increases the thermal energy of the probe and decreases the viscosity of the aqueous solution. This can lead to faster sandwich bond

formation rates and higher switching activities. These topics are beyond the scope of the present paper.

- Change: No change.

21. L584: Emphasizing the usefulness of the presented model, however, no data availability statement, code repository and programming language is mentioned in the text.

- Answer: We thank the reviewer for pointing this out.
- Change: We have included this in the manuscript.

For the provided supplementary information:

22. L92: As per point 3 above, model designed to probe independent events, however, cooperativity will most likely play a crucial role for the sensor readout. Could the model be revised to include cooperative binding/simulate the effects of cooperative binding?

- Answer: See the answer to question 1 of Reviewer #2.
- Change: See the corresponding changes.

23. L169: NB0 the number of binder molecules on the sensing surface. Does this include the surface of the bead as well, or only the flat surface of the sensor?

- Answer: The calculation includes binders molecules on both probe-side and surface-side, see Supplementary Table 2.
- Change: No change.

24. L208: Nbinders per side is 30, however once the bead is bound, a fraction of the binders will not be able to perform a sensor readout, since they might be on the volume-facing side of the bead. How one would account for that?

- Answer: This section discusses a generic nanoswitch design and the calculations are not tailored to BPM. For BPM, the number of volume-facing binders would also need to be included in $N_{binders}$.
- Change: No change.

25. L214: intra-nanoswitch binding has not been studied extensively – but has it been studied? Would there be references that could help improve the model and put it in the perspective of other works?

- Answer: Sandwich nanoswitches have been demonstrated with transduction based on Förster resonance energy transfer (FRET) (ref. 18 and 37), redox current (ref. 17) and particle motion (ref. 19 and 20). The FRET- and redox-based studies show dose-response curves and time profiles, but not enough information to allow testing of the simulation model.
- Change: No change.

26. L235: Sup. Fig 2C: blue unbound lifetimes are missing datapoints for the first 3 concentrations

- Answer: We apologize for the unclarity. Characteristic unbound-state lifetime values for the lowest analyte concentrations could not be reliably determined due to right-censoring of the states, i.e., the measurement duration was too short with respect to the characteristic lifetimes.
- Change: We have clarified this topic in the SI.

27. L332f: Please state the fact of missing cooperative effects more clearly.

- Answer: See the answer to question 1 of Reviewer#2.
- Change: See the corresponding changes.

28. L463: Figure 6- concerning comment on L208. Is increasing the number of binders on the probe side also considering the geometric constrains of the sensor?

- Answer: Currently the model does not take into account any geometric constraints.
- Change: See previous answers related to cooperativity.

29. L500: Figure 7: Can you add a direct comparison between the model and the experimental data in one graph? Can you infer using you model how many binders were on the respective beads? Can you provide exemplary data images of the beads, the determination of the activity and the respective classification?

- Answer: It is not yet possible to fit the model to the experiments, because too many parameters are unknown. Therefore, the comparisons between experiments and simulations focus on specific aspects: the shapes of the curves, the scaling of EC50, and the scaling signal variability. The model can infer relative amounts of binders on the beads; the exact number of binders depends on the rate parameters, which are not independently known. We plan to expand on this topic in future work. We agree with the reviewer that additional information on BPM data processing is helpful.
- Change: We added details on BPM data processing in the SI, see Supplementary Note 2.1.

30. L707: 100 nanoswitches within 15 simulations, that contained 10 000 nanoswitches. Which is the correct number simulated?

- Answer: We simulated 15 runs of 10000 nanoswitch responses. Each run (each run containing 10000 nanoswitches) was partitioned into 100 sets of 100 nanoswitches. This gave 15 runs that contained 100 sets of 100 nanoswitch responses. For each set, we then calculated the sampling distribution which was comprised of 15 mean responses over the 100 nanoswitch responses. The standard deviation of that sampling distribution corresponds to s_s. This gives 100 values for s_s.
- Change: We have clarified this in the SI.

31. L798: Fig 16 A. Why are the datapoints shifted in respect to time, since they were acquired every 15 minutes? B, C red open markings are described in the figure description, yet no markings are seen.

- Answer: Each measurement lasted for 15 minutes, the consecutive measurement did not start immediately after the previous measurement. We thank the reviewer for pointing out the inaccuracy in the caption: the caption referred to a previous version of the figure.
- Change: We changed the figure caption accordingly.

32. L805: temporal drift. Can you comment which kind of drift? Drift due to exposure and processing or drift of the sample itself?

- Answer: The drift is related to long-term sensor changes such as losses of binder molecules and non-specific interactions (see L785-787, ref. 7, Cajigas et al. *ACS Sens.*, 2024, and ref. 10, Vu et al., *Adv. Sci.*, 2025, in the SI).
- Change: No change.

33. L808: Describing the distribution as normally distributed appears stretched with the number of counts and bins.

- Answer: We agree with the reviewer that the number of counts are low to determine whether the distributions are actually Gaussian.
- Change: We have changed this in the SI.

34. L826: Fig 18. Can you provide a legend for the different colors in the same figure? This would improve its clarity.

- Answer: We thank the reviewer for pointing this out.
- Change: We have added a legend.

35. L832: Adding to this chapter, could you also analyze the previous published system using the antibodies and lactoferrin using your model?

- Answer: The previous system and other BPM sensors could indeed be analyzed using the same methodology. In future work, we plan to use this framework to aid BPM sensor development for other applications.
- Change: No change.

36. L898: Simulations using a background activity between 10-20 mHz would prevent the readout low picomolar analytes (see main figure 2d inset) as the activity itself will be above readout activity. Can you comment on this?

- Answer: Indeed, high background activity values would prevent a low picomolar readout. The challenge for the future is to suppress these non-Poissonian sources of noise in order to have fully Poisson-limited single-molecule sensors.
- Change: No change.

37. L952ff: Draws a conclusion about the nanoswitch sensors that would also fit the main text.

- Answer: We agree with the reviewer that this can be included in the main text.
- Change: We have expanded on this topic in the Discussion and Conclusions.

38. L978: Can you comment on the buoyancy of the polystyrene beads?

- Answer: The beads are comprised of an iron oxide core surrounded by polystyrene. The iron oxide makes the beads more dense than water, meaning that they sediment in aqueous solutions.
- Change: No change.

39. L1006: PSB? Did you mean PBS?

- Answer: PSB here refers to the particle-side binder, not PBS buffer.
- Change: To avoid confusion, we changed ‘PBS’ to ‘PBS buffer’ throughout the manuscript.

40. L1008: How did you validate the DNA loading on the beads?

- Answer: DNA loading was validated by checking whether the DNA-coated particles responded to different ssDNA-target concentrations.
- Change: No change.

41. L1010: PSB again

- Answer: See the previous answer related to PBS/PSB.
- Change: See the previous change related to PBS/PSB.

42. L1012: What is HS buffer?

- Answer: We thank the reviewer for pointing this out. HS buffer refers to PBS buffer supplemented with 0.5 M of NaCl.
- Change: This has been changed accordingly in the manuscript.

Reviewer #1 (Remarks to the Author):

The authors have satisfactorily addressed my concerns in this revision. I suggest publication of this manuscript as-is.

- We thank the reviewer for their feedback.

Reviewer #2 (Remarks to the Author):

The revised article addresses many of the mentioned points mentioned in the initial review and integrated substantial changes to the main text and even more elaborations in the Supplementary information section. Since cooperativity is, as explained by the authors, not in scope of the current approach, I deem the article to be ready for acceptance after minor revisions due to small discrepancies.

1. In the answer to Reviewer 1, 2nd answer, SI Section 7.2 is mentioned, but does not exist in the submitted document. The Box 1 which is mentioned is however present.

- We thank the reviewer for pointing this out, the header and two paragraphs were accidentally deleted during editing. They have now been re-added, there were no changes with respect to the initial submission.

2. Within the new Box1: The formular for the signal itself and its variability is the same. Later in the box, the variability is mentioned as the square root of the signal. This might be a typo.

- We again thank the reviewer for their sharp eye, this is indeed a typo.

3. On the answer to Reviewer 2, 3rd statement: Sup. Figure 8 is mentioned; however, the reference would be matching Figure 9, lower.

- We indeed meant to refer to Supplementary Figure 9.

4. Arthur M. De Jong is still missing the affiliation, but has author contribution

- The affiliation has now been added.
